# Pitfalls in complement analysis: A systematic literature review of assessing complement activation

**DOI:** 10.3389/fimmu.2022.1007102

**Published:** 2022-10-18

**Authors:** Ricardo J. M. G. E. Brandwijk, Marloes A. H. M. Michels, Mara van Rossum, Aline H. de Nooijer, Per H. Nilsson, Wieke C. C. de Bruin, Erik J. M. Toonen

**Affiliations:** ^1^ R&D Department, Hycult Biotechnology b.v., Uden, Netherlands; ^2^ Radboud Institute for Molecular Life Sciences, Department of Pediatric Nephrology, Amalia Children’s Hospital, Radboud University Medical Center, Nijmegen, Netherlands; ^3^ Department of Internal Medicine and Radboud Center for Infectious Diseases, Radboud University Medical Center, Nijmegen, Netherlands; ^4^ Department of Immunology, University of Oslo and Oslo University Hospital Rikshospitalet, Oslo, Norway; ^5^ Linnaeus Centre for Biomaterials Chemistry, Linnaeus University, Kalmar, Sweden; ^6^ Department of Chemistry and Biomedical Sciences, Linnaeus University, Kalmar, Sweden

**Keywords:** complement system, complement diagnostics, systematic review, immunoassays, standardization of measurements, complement activation

## Abstract

**Background:**

The complement system is an essential component of our innate defense and plays a vital role in the pathogenesis of many diseases. Assessment of complement activation is critical in monitoring both disease progression and response to therapy. Complement analysis requires accurate and standardized sampling and assay procedures, which has proven to be challenging.

**Objective:**

We performed a systematic analysis of the current methods used to assess complement components and reviewed whether the identified studies performed their complement measurements according to the recommended practice regarding pre-analytical sample handling and assay technique. Results are supplemented with own data regarding the assessment of key complement biomarkers to illustrate the importance of accurate sampling and measuring of complement components.

**Methods:**

A literature search using the Pubmed/MEDLINE database was performed focusing on studies measuring the key complement components C3, C5 and/or their split products and/or the soluble variant of the terminal C5b-9 complement complex (sTCC) in human blood samples that were published between February 2017 and February 2022. The identified studies were reviewed whether they had used the correct sample type and techniques for their analyses.

**Results:**

A total of 92 out of 376 studies were selected for full-text analysis. Forty-five studies (49%) were identified as using the correct sample type and techniques for their complement analyses, while 25 studies (27%) did not use the correct sample type or technique. For 22 studies (24%), it was not specified which sample type was used.

**Conclusion:**

A substantial part of the reviewed studies did not use the appropriate sample type for assessing complement activation or did not mention which sample type was used. This deviation from the standardized procedure can lead to misinterpretation of complement biomarker levels and hampers proper comparison of complement measurements between studies. Therefore, this study underlines the necessity of general guidelines for accurate and standardized complement analysis

## Introduction

Complement is a key innate immune system and is part of the first line of defense against pathogens. It is recognized as being evolutionary among the oldest pathogen recognition systems, and not only has this system a critical role in killing microbes, it also bridges innate and adaptive immune responses. The realization that an increasing number of human diseases are, at least partly, complement-mediated has driven a renewed interest in complement. Accurate analysis of complement components and complement activation is of utmost importance for diagnosing disease and/or therapy monitoring (1–5). However, reliably determining the complement status of individual patients has proven to be challenging (1, 5). Measured concentrations of complement system proteins vary widely among different laboratories due to non-unified protocols for pre-analytical sample handling, including collection, processing, and storage. Also, laboratories do not always use the correct sample type for the method or assay used. Other reasons for inconsistencies in concentrations are the lack of uniform and recognized calibrators, the use of different techniques, reagents, and antibodies recognizing different epitopes in the antigen. This indicates the high need for standardization of techniques, assays and biomaterials in combination with proper pre-analytical sample handling and storage (2, 3).

This review aimed to provide an overview and general guidelines for measuring complement activation in a reliable and standardized way. We performed a systematic analysis of the literature regarding current methods used to assess complement C3, C5 and/or their split products and/or the soluble terminal complement complex (sTCC; also referred to as sC5b-9 or membrane attack complex, MAC) in human blood. We reviewed whether the identified studies performed their complement measurements according to the recommended practice regarding pre-analytical sample handling and assay technique. Results were supplemented with own data, not included in the systematic review, to illustrate the importance of using the correct biomaterials, methods, and techniques for the assessment of complement components.

## Complement pathway activation

The complement system comprises of approximately 50 soluble and cell surface-bound proteins organized to eliminate structures recognized as dangerous. These danger signals include invading microorganisms, apoptotic and necrotic cells and immune complexes (6, 7). Furthermore, complement can link innate and adaptive immune responses by mediating regulation of T cell and B cell responses (8). More recently, several studies reported a role for intracellular complement (9–11).

**Figure 1:**
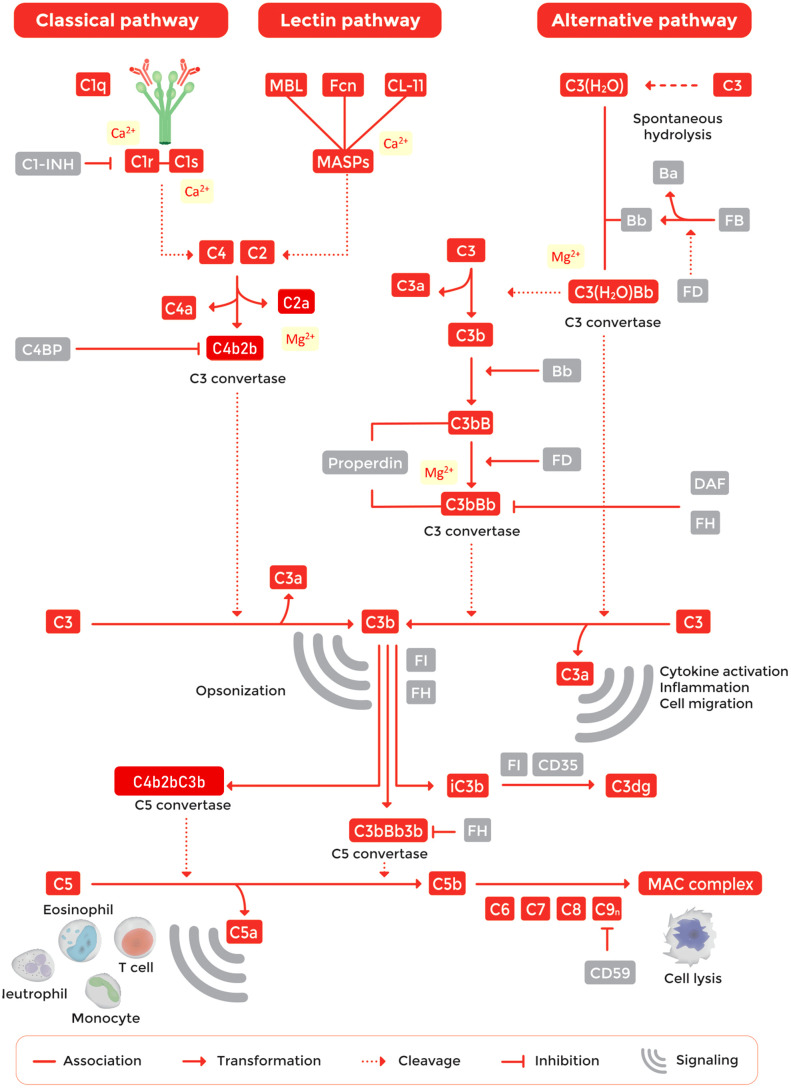
Overview of the complement pathway.

The complement pathway is tightly regulated by a network of proteins to avoid uncontrolled activation (**Figure 1**) (12). The system is organized in three activation pathways: the classical pathway (CP), the lectin pathway (LP) and the alternative pathway (AP). Each pathway is activated by different molecules. The CP is activated by the binding of C1q to antigen-bound-IgM or -IgG hexamers forming immune complexes. The LP is activated by binding of one of the recognition molecules mannose-binding lectin (MBL), collectin 10, 11 or 12, or ficolin-1, -2, or -3 to microorganism-associated molecular patterns (MAMPs) or carbohydrate structures located on damaged cells. The AP is continuously activated at a low level as a result of spontaneous C3 hydrolysis allowing for activation on surfaces that lack proper complement regulation, typically non-host surfaces (12–15). Pathway activation results in the cleavage and activation of complement C3 and C5. Subsequently, this will lead to the generation of active fragments such as C3a, C3b, iC3b, C3dg, C4a, C4b and C5a and the formation of TCC (also known as MAC or C5b-9) (1). While TCC is inserted into pathogen cell membranes, resulting in cell death of sensitive cells, typically Gram-negative bacteria, the other activation fragments bind to their corresponding complement receptor expressed by various cell types. Binding of activation products to these receptors leads to a variety of biological responses such as phagocytosis, cell migration, chemotaxis, cytokine production, cell activation and modulation of pattern recognition receptor (PRR)-induced responses (**Figure 1**) (12, 13, 16–18).

Inadequate functioning of the complement system, e.g. by component deficiencies or exaggerated activation, has been linked to numerous diseases, both rare and common, and the list is growing continuously (**Table 1**) (13, 72–75). An imbalance in complement activation and regulation can lead to life-threatening conditions such as coagulation dysregulation, systemic inflammation and failure of organs such as the eyes, kidneys, skin, brain and vascular system (75–77). Because of the large number of complement-mediated diseases, together with recent results from large-scale genomics and proteomic studies (78, 79), the interest in the complement system has renewed, especially as a promising target for therapeutic intervention. This was demonstrated more than a decade ago by the first complement-specific drug, eculizumab (Soliris, Alexion), targeting the key complement component C5 (29). In recent years, human complement studies have become very important in drug delivery, nanomedicine and biomaterial fields, as elegantly summarized by Moghimi and coworkers (80, 81). Interestingly, assessment of complement within these fields is often performed using incorrect or suboptimal assays and samples that were inappropriate handled regarding collection, storage and pre-analytical preparations (80). As complement is involved in an increasing number of human diseases, standardized measurement of complement components is essential (1–5, 82). However, accurate determination of the complement status of individual patients has proven still to be challenging and needs to be performed in a uniform way.

**Table 1:** Overview of complement-related diseases.

Disease	Mechanism	Recommended analysis	Ref.
Age-related macular degeneration (AMD)	Genetic variants in complement genes *C3*, *CFB CFH*, and *CD46 (MCP)*.	Genetic analysis of the genes *C3, CFB, CFH, CD46*	(19)
Alzheimer disease (AD)	Local generation of C3a and C5a creating an inflammatory environment. Altered levels of C1q, FB, C4, C4a, TCC. Deposition of TCC on neuronal cells may be responsible for neuritic loss.	Analysis of complement components C1q, FB, C4, C4a and TCC	(20, 21)
Amyotrophic lateral sclerosis (ALS)	Dysregulation of complement, complement activation in the spinal cord, C5aR1-C5a signalling during progression suggests that the terminal complement pathway contributes to the pathogenesis of ALS.	Analysis of terminal pathway components, such as TCC	(22)
ANCA-associated vasculitis	Signalling through the C5a-C5a receptor, contributes to the immunopathology of ANCA-associated vasculitis	ELISA for C5a	(23, 24)
Anti-glomerular basement membrane (anti-GMB) disease	Autoantibodies- or immune complexes-induced complement activation	ELISA for anti-C1q antibodies	(25, 26)
Angioedema	Genetic variants in C1-INH (Hereditary angioedema; HAE), autoantibodies against C1-INH (acquired angioedema)	C1-INH, C4, C1q, anti-C1-INH autoantibodies, molecular analysis C1-INH.	(27)
Atypical haemolytic uremic syndrome (aHUS)	Genetic variants in *C3, CFB, CFH, CFI, CD46 (MCP)* genes.	CH50, AH50, functional ELISA for CP, AP, ELISA for C3, FB, C3a, C3d, TCC, FH, FI, anti-FH autoantibodies, molecular analysis *CFH, CFI, C3, CFB, CD46 (MCP)*	(28)
Cancer	Complement-induced inflammation leading to tumour growth and metastasis.	Analysis of terminal pathway components, such as TCC	(29)
Cold agglutinin disease	Cold agglutinin (CA) IgM immune complexes bind C1q and thereby initiates the classical complement pathway	CH50	(30)
Diabetes	Increased levels of complement activation in plasma.	Analysis of terminal pathway components, such as C3d and TCC	(31)
Epilepsy	Deposition of C1q, C3b and TCC in affected brain tissue.	Analysis of components such as C1q, C3, C5 and TCC	(32)
Glomerulopathies (C3G)	Genetic variants in *C3, CFB CFH, CFI* and *CFHR5*; presence of autoantibodies (C3NeF) preventing AP regulation.	CH50, AH50, functional ELISA for CP, AP, ELISA for C3, C4, C3a, C3d, TCC, FH, FI, C3 and C4 nephritic factor, autoantibodies to FH and FB, C3 convertase, molecular analysis of CFH, CFI, C3, FB, MCP (CD46)	(33–35)
Guillain–Barré Syndrome (GBS)	IgG anti-ganglioside antibodies that lead to GBS cause complement-mediated disruption of interactions between Schwann cells and axon. Terminal complement activation and TCC deposition on Schwann cell. Elevated levels C3a and C5a in the CSF of patients	ELISA for C3a, C5a, TCC. Measurement of anti-ganglioside antibodies	(36)
Henoch-Schoenlein purpura [Immunoglobulin A vasculitis (IgAV)]	Most common form of childhood vasculitis, characterized by IgA1-immune deposits, complement factors and neutrophil infiltration, which is accompanied with vascular inflammation. Autoantibodies- or immune complexes-induced complement activation *via* the alternative and lectin pathway. Levels of TCC in the urine might be a useful indicator of renal injury	Analysis of terminal pathway component TCC	(37, 38)
Hypocomplementemic Urticarial Vasculitis Syndrome (HUVS)	Exact pathophysiology unknown. Rare immune complex-mediated small vessel vasculitis characterized by urticaria, hypocomplementemia (low C1q, C3, and C4), and systemic manifestations, and it is also associated with circulating anti-C1q autoantibodies	ELISA for C1q, C3, C4, autoantibodies against C1q	(39, 40)
Huntington’s disease	Autosomal dominant neurodegenerative disorder caused by a cytosine-adenine-guanine (CAG) trinucleotide repeat expansion in the huntingtin gene. Upregulation of complement components C7 and C9 in HD, increased levels of clusterin are associated with disease progression. Upregulation of C5a-C5aR axis.	ELISA for C5a, C7, C9, TCC	(36, 41)
Infection disorders	Pathogens trigger innate immune responses and activate the complement pathway either directly by pathogen-derived antigens or indirectly by molecules released by host cells binding to these antigens.	CH50, AH50, functional ELISA for CP, AP, LP. ELISA for C3, C4, C1q, C3a, C3d, TCC, C5-C9, Properdin, MBL	(42, 43)
Inflammatory bowel disease	Appropriate activation of the intestinal complement system seems to play an important role in the resolution of chronic intestinal inflammation, while over-activation and/or dysregulation may worsen intestinal inflammation. C3 and C4a are suggested as diagnostic markers	ELISA for C3 and C4a	(44, 45)
Kidney ischemia/reperfusion (I/R) injury	Complement molecules influence function of factors such as free radicals, neutrophils, and the products of activated endothelium. Complement activation releases biologically active, proinflammatory products. C4a, C3a, and C5a can induce smooth muscle contraction, increase vascular permeability, and cause the release of histamine. C5a acts on neutrophils, promoting chemotaxis and activation and acts on neutrophils and endothelium to upregulate cell adhesion molecules such as CD11b/CD18 and the intercellular adhesion molecule (ICAM-1). C5b-9 inserts into the membrane of target cells, inducing cell injury and necrosis. C5b-9 also activates neutrophils and endothelium by upregulating adhesion molecules and promoting the release of cell stimulants such as hydrolytic enzymes, reactive oxygen species, arachidonic acid metabolites, and cytokines. In addition, C5b-9 can enhance the procoagulant properties of the endothelium	ELISA for C3/C3 split products, C4/C4 split products, C5/C5a split products, TCC, FH, properdin. C4d staining of kidney biopsies	(46–49)
Kidney transplant injury	Antibody-mediated renal injury; individuals with pre-existing circulating antibodies are at high risk to develop complement-dependent reaction against transplanted kidney; local synthesis of complement components together with loss of regulatory mechanisms for complement activation, especially of C3, C5 and TCC.	ELISA for C3/C3 split products, C4/C4 split products, C5/C5a split products, TCC, FH, properdin. C4d staining of kidney biopsies	(47, 49, 50)
Multiple sclerosis	Inflammatory, demyelinating disease of the central nervous system that leads to variable axonal and neuronal damage. Causes are largely unknown. Depositions of C1q, C3d, and C5b-9 in white matter lesions of MS. Complement proteins, activation products and inhibitors were found at MS plaques. Astrocyte-enriched extracellular vesicles derived from the plasma of MS patients contained high levels of C1q, C3, C3b, iC3b, C5, and C5a. C3a in CSF at baseline assessment of patients with clinically isolated syndrome and newly diagnosed relapsing-remitting MS could be a promising prognostic marker of disease activity Also genetic variants were identified, for instance in MASP1 and C2 to be associated with the disease.	ELISA for C3 and C3 activation products such as C3a	(32, 36)
Myasthenia gravis (MG)	MG is an antibody-mediated autoimmune disease of the postsynaptic neuromuscular junction presenting with a fluctuating degree of weakness in ocular, bulbar, limb, and respiratory muscles. Activation of complement *via* disease-specific autoantibodies to the acetylcholine receptor (AChR). Inhibition of C5 or TCC could be beneficial in refractory MG	ELISA for C5/C5a, TCC	(36, 51)
Myocardial infarction	Complement activation is an important factor for inflammation and injury of ischemic and infarcted myocardial tissue. Lectin pathway main driver in myocardial reperfusion injury. Complement-derived effector molecules as described above are involved in producing the inflammation, tissue injury, and necrosis of the heart tissue during reperfusion	Analysis of terminal pathway component C3/C3 split products, C5/C5 split products and TCC	(52)
Neuromyelitis optica	Neuromyelitis optica spectrum disorder (NMOSD) is an autoimmune inflammatory disease of the central nervous system (CNS), characterized by pathogenic, complement-activating autoantibodies against the main water channel in the CNS, aquaporin 4 (AQP4)	Anti-AQP4 autoantibodies	(53)
Organ transplantation	C3 is activated during transplant and is associated with late allograft damage and rejection. C5a and C3a anaphylatoxins increase the inflammatory response. C5a fragment serves as a chemotactic agent for neutrophils and macrophages, there is also evidence that C3a induced chemotaxis of monocytes and mast cells. In Antibody-mediated rejection (AMR), donor-specific antibodies (DSAs) form immune complexes on the surface of (kidney) allograft endothelium bind C1q complement component, leading to the activation of the classical complement pathway. The C4d fragment formed following this activation, and deposited in the peritubular capillaries of the kidney allograft, is an important biomarker of antibody-mediated rejection	ELISA for C3/C3 split products, C4/C4 split products, C5/C5a split products, TCC, FH, properdin. C4d staining of kidney biopsies	(29, 47, 54)
Parkinson disease (PD)	PD is characterized by dopamine deficiency at the basal ganglia and deposition of a-synuclein that forms Lewy bodies. However, the exact neuropathologic mechanisms remain elusive. Studies regarding the role of complement in PD are controversial. Some reported the involvement of the classical complement pathway by recognizing anti C3d, C4d, C7 and C9 antibodies in substantia nigra or by the aggregation of iC3b and C9 in Lewy bodies of PD patients. Other studies failed to correlate complement activation with cortical Lewy Bodies. Clusterin and complement C1r were decreased compared to controls and have been proposed as useful biomarkers of disease progression	ELISA for clusterin and C1r	(36, 55–58)
Paroxysmal nocturnal haemoglobinuria (PNH)	PNH is a rare disease that presents clinically with a variety of symptoms, the most prevalent of which are hemolytic anemia, hemoglobinuria, and somatic symptoms including fatigue and shortness of breath. In patients with PNH, CD55 and CD59 are lacking, and RBCs undergo excessive complement-mediated hemolysis leading to hemolytic anemia, thrombosis, renal dysfunction, and pulmonary hypertension	CD55, CD59, fluorescently labeled aerolysine (FLAER) test	(59)
Periodontitis	Inflammatory disease in tooth-supporting tissues, induced by bacteria growing in a biofilm on tooth surfaces. Components of the complement system are present in the periodontal tissue leading to local induced complement activation. C1q, factor B, factor Bb, C3, C3a, C3b, C3c, C3d, C4, C5, C5a, C5b and C9 have all been detected in diseased periodontal tissue	ELISA for C1q, factor B, factor Bb, C3, C3a, C3b, C3c, C3d, C4, C5, C5a, C5b and C9	(60)
Polytrauma	Evidence of systemically increased complement activation: C3a, C5a, high C3a/C3 ratio in case of organ dysfunction after trauma. Predominance of alternative pathway activation. C5a is seen to delay the fracture healing by delaying neutrophil apoptosis and delaying recruitment of osteoblast and osteoclast progenitors.	CH50, MBL, C3a, C5a, SC5b-9, C4BP total, C4BP-β, and factor I by ELISA	(61–63)
Refractory generalized myasthenia gravis (gMG). See also myasthenia gravis	gMG is a rare autoimmune disorder characterized by skeletal muscle weakness caused by disrupted neurotransmission at the neuromuscular junction. Autoantibodies attack acetylcholine receptors, which are essential in facilitating muscle contraction and movement.	ELISA for C5/C5a, TCC	(29, 36)
Rheumatoid arthritis	Evidence suggesting complement activation in synovial tissue. Increased levels of C2, C3 and C3a in RA patients	C2, C4b, C5a, FD, MBL, FI, C1q, C3,C3a, C3b C4, FB, FH, properdin, TCC	(64)
Schizophrenia	Genetic variant in *C4A* associated with increased risk. Increased copy number of *C4A*	Genetic variant analysis *C4A*	(65)
Sepsis/multi-organ dysfunction	Hyperinflammatory response after infection (cytokine storm). Increased levels of C3a, C5a and TCC. Excessive complement activation and consumption during septic shock.	ELISA for C3, C3a,C3c, C5, C5a, and TCC (sC5b-9), CH50	(66–68)
Skin diseases	Excessive complement activation, may be caused by autoantibody-induced cytotoxic effects of TCC on epidermal or vascular cells thereby causing inflammation.	Analysis of terminal pathway components, such as TCC	(69)
Systemic lupus erythematosus (SLE)	Genetic variants in *C1Q, C2, C4, CR2, CR3*; impairment of complement-mediated clearance of immune complexes and apoptotic cells. Autoantibodies against C1q.	CH50, functional ELISA for CP. ELISA for C4 (C4a/b), C1q, C3, C3a, C3d, TCC, anti-C1q autoantibodies	(39, 40)
Thrombotic thrombocytopenic purpura (TTP)	Excessive AP activation. Functional deficiency of ADAMTS13 plays a substantial role in the development of TTP. Increased complement activation, increased levels of complement activation markers C4d, C3bBbP and C3a.	C3, Factors H, I, B and total alternative pathway activity together with complement activation fragments (C3a) or complexes (C1rs-INH, C3bBbP, sC5b9) measured by ELISA or RID.	(70)
Trauma (see also polytrauma)	Evidence of systemically increased complement activation: C3a, C5a, high C3a/C3 ratio in case of organ dysfunction after trauma. Predominance of alternative pathway activation. C5a is seen to delay the fracture healing by delaying neutrophil apoptosis and delaying recruitment of osteoblast and osteoclast progenitors.	CH50, MBL, C3a, C5a, SC5b-9, C4BP total, C4BP-β, and factor I by ELISA	(61–63)
Urticarial vasculitis (see also Hypocomplementemic Urticarial Vasculitis Syndrome)	Exact pathophysiology unknown. Rare immune complex-mediated small vessel vasculitis characterized by urticaria, hypocomplementemia (low C1q, C3, and C4), and systemic manifestations, and it is also associated with circulating anti-C1q autoantibodies	ELISA for C1q, C3, C4, autoantibodies against C1q	(39, 40)
Uveitis	Inflammation of the uvea, the pigmented layer that lies between the inner retina and the outer fibrous layer composed of the sclera and cornea. Presence of autoantibodies activating complement.	Human C3a radioimmunoassay Immunoelectrophoresis (IEP)	(71)

To provide insight into the current practice in complement measurements at individual labs, we conducted a systematic literature search in which we reviewed which sample type and techniques were used for the assessment of complement activation in the past five years. The studies were assessed for their quality based on the recommended guidelines for studying complement activation as described below.

## Current guidelines for assessing complement activation

For accurate complement analysis, it is important that the complement activation is blocked at blood sampling since activation *ex-vivo* during sample processing may alter the results of interest (2, 12). Hence, appropriate pre-analytical sample handling is a necessity to avoid erroneous results. For complement analysis, samples may be assessed either for complement function or quantification of individual complement components (2, 3, 83). Both types of analysis require a specific approach regarding the sample type, pre-analytical sample handing and measurements.

### Assessment of complement function

**Figure 2:**
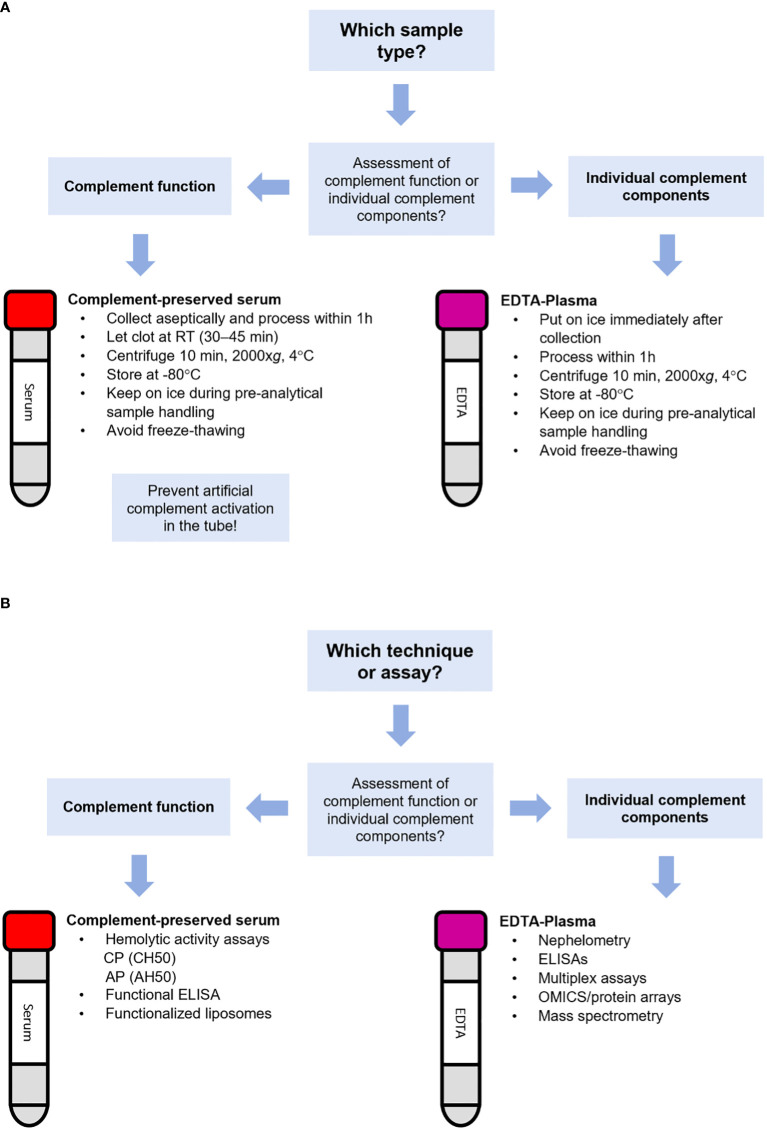
Flowchart for the selection of the appropriate sample type and technique for complement analysis. Flowchart for selection of **(A)** the appropriate sample type or **(B)** the appropriate technique for the assessment of complement.

For assessing the ability of complement activation, serum samples should be used (84). Whole blood should be collected in serum tubes. These collection tubes are available either with or without a clot activator (e.g. silica). Note that some complement analyses may be affected by the type of collection tube, e.g. ficolin 2 is depleted in serum retrieved from tubes with silica, which should especially be taken into account when assessing the lectin pathway (80, 85, 86). Let blood coagulate for approximately 30 min at RT. Please be aware that blood coagulation kinetics are slightly slower in tubes without a clot activator, so ensure complete clot retraction. Optimize if needed. For both type of serum collection tubes, separate the serum fraction by centrifugation (2000x*g*, 10 min) at 4°C. Process serum within 1 h. Complement activation is temperature-dependent so always keep serum on ice to avoid *ex-vivo* complement activation (12, 80, 81, 87, 88). Store serum samples at -80°C since storage at higher temperatures such as -20°C will create a slow freezing rate that allows for complement activation. Mechanical stress (rigorous shaking or vortexing) and repeated cycles of freezing and thawing should be minimized as much as possible since every cycle may increase complement activation (2, 84) (**Figure 2A**). Next to serum, also Hirudin-plasma (or Lepirudin; recombinant Hirudin) can be used for the assessment of complement function as Hirudin blocks coagulation but allows complement activation (when used in low concentrations: 0.2 IU/mL and 2 IU/mL) (89). As complement is also activated by a low pH (<7.1), it is advised to monitor and control it (with appropriate buffers) to avoid erroneous measurements (90).

For studying complement function, it is of utmost importance that the appropriate buffers are used. For assessing CP and LP activation, buffers containing both Ca^2+^ and Mg^2+^ should be used. These cations are required in order to form protein complexes which initiate or mediate progression of complement activation. Ca^2+^ is essential for the activation of CP and LP protease complexes that cleave C4 and C2 (91, 92), whereas Mg^2+^ is needed for the binding of C2 to C4b to form CP/LP C3 convertases (93). For studying AP activation, CP and LP activity must be blocked by adding ethylene glycol tetraacetic acid (EGTA) to chelate Ca^2+^ (**Figure 1**). Additionally, an optimal concentration of Mg^2+^ is required since the binding of Factor B to C3b to form the AP C3 convertase is Mg^2+^-dependent (3, 94). Traditional complement buffers like GVB++ (gelatin veronal buffer) contain 0.15 mM Ca^2+^ and 0.5 mM Mg^2+^. Hirudin or lepirudin do not interfere with divalent cations.

For assessing complement function, several techniques and assays are available (**Figure 2B**). Traditionally, complement function of the CP and AP was assessed using respectively the CH50 and AH50 hemolytic assays. These assays detect reduction, absence and/or inactivity of several components of the complement pathway. The CH50 is based on the capacity of serum to activate complement *in-vitro* and subsequently lyse sheep erythrocytes opsonized with anti-sheep antibodies (usually rabbit IgG) *via* the CP. The AH50 is based on complement-dependent lysis of rabbit erythrocytes, which cannot recruit the soluble human complement regulator Factor H, thereby allowing for AP activation (95). If one or more complement components are absent (e.g. due to a genetic deficiency) or their levels decreased (e.g. due to overactivation leading to consumption), hemolytic activity is decreased. When both assays are performed side-by-side, they can indicate C3 and terminal pathway (TP) deficiencies (C5, C6, C7, C8, and C9; absence of hemolysis in both CH50 and AH50), CP deficiencies (C1, C2, C4; absence of lysis in CH50 only) and AP deficiencies (Factor B, D, H, I, properdin; absence of lysis in AH50 only). Factor H and Factor I deficiency can also result in C3 consumption as a result of uncontrolled AP activation, leading to low CH50.

Complement function can also be assessed using functionalized enzyme-linked immunosorbent assays (ELISA), which are considered more accurate and reproducible and are less prone to variation. This type of assays uses plates coated with either IgM, mannose or lipopolysaccharides (LPS) to examine CP, LP or AP activation respectively. In the presence of the appropriate buffers, complement is activated in the patient’s serum sample (at 37°C). Next, pathway activation is assessed by the detection of C5b-9 in the terminal pathway using a specific conjugate antibody directed to a neo-epitope in C5b-9 (12, 96, 97). These assays require a relatively high sample dilution to prevent cross-pathway interference. However, using a sample dilution that is too high might lead to false negative results. When concentrations for critical complement components needed for complement activation are too low, the pathway will not be activated. As the window for the correct sample dilution is rather small, it is easily missed. Therefore, this high sample dilution may be considered as a drawback when these types of assays are used.

A third type of assay for assessing complement function is the liposome immunoassay. This type of assay consists of liposomes coated with antigens (for instance with dinitrophenyl (DNP)) and containing a reporter molecule (signal-producing molecules). When serum is mixed with the liposomes and an antibody-containing reagent (in this case anti-DNP), the formed immune complex will trigger complement activation resulting in disruption of the liposomes and release of the entrapped reporter molecule from the lysed vesicles. The degree of reporter release corresponds to the degree of complement activation and can easily be measured (98, 99).

### Assessment of individual complement components

For analysis of individual complement components, EDTA-plasma should be used having at least a final concentration of 10 mM (82, 100), especially when measuring activation products. Using EDTA-plasma, any further activation of complement is minimized (but not completely excluded) since EDTA chelates Ca^2+^ and Mg^2+^ thereby blocking the function of the C1 complex and the C3 convertases (4, 83, 100–102). Of note, nafamostat mesylate (0.2 mg/mL) can be used in addition to EDTA to maximize inhibition of artificial complement activation (100). Heparin-plasma should be avoided as heparin interacts with many complement proteins and regulators such as C1, C1q, C1-INH, C2, C4, C4b, C4BP, MASP-1, MASP-2, C3, C3b, FB, FD, FH, properdin, C6, C8, C9 and vitronectin, thereby affecting complement function (84, 103). Low levels of heparin (<2 IU/mL) can activate complement whereas high levels (20 IU/mL) inhibit complement activation (89). If possible, we recommend to also avoid citrate-plasma for the assessment of complement components. Although differences in concentrations for markers such as C4d, Bb, C3a, C5a and sTCC are very small between freshly processed EDTA- and citrate-plasma samples, these markers are more stable in EDTA-plasma when stored longer at either RT or 4°C before processing. Citrate-plasma also tends to be more sensitive for freeze-thawing (83, 104). Serum cannot be used for the assessment of individual complement components, especially activation products. Due to the absence of complement inhibitors, artificial complement activation during collection, shipment, processing and/or pre-analytical sample handling will cause an increase of many complement components making reliable determination of these markers impossible (83, 104).

EDTA-whole blood should be kept on ice and centrifugated at 4°C within 60 minutes after collection. Keep transportation and logistics of samples from the patients to the lab as short as possible and handle samples with care (keep on ice, minimize mechanical stress). Isolated EDTA-plasma should be kept on ice and as soon as possible snap-frozen and placed in -80°C. Shipment of EDTA-plasma for complement analysis should always be done on dry ice. EDTA-plasma for complement marker analysis should be thawed and kept on ice and repeated freeze-thawing should be avoided. Different markers have various sensitivity to freeze-thawing (100). Note that EDTA-plasma should not be used for functional complement testing in the assays described above as these assays detect *in-vitro* complement activation which is inhibited by EDTA (1) (**Figure 2A**).

Several techniques for analysis of complement components are available (**Figure 2B**). In most clinical laboratories, analysis of individual complement components such as C3, C4 and sometimes C5 is performed by nephelometry or turbidimetry. These techniques are widely used in clinical laboratories because they are relatively easily automated and inexpensive. However, a major drawback of nephelometry and turbidimetry is that it cannot distinguish between the native non-activated complement protein and its activated split products. For instance, for C3 it will detect both the intact non-activated C3 and its activated proteolytic fragments C3a, C3b, iC3b, C3c and/or C3d (105). Another drawback of is that these methods use a relatively large sample volume. This is especially a challenge for complement analysis in young children, of whom often only small amounts of sample is available.

ELISA is a technique also widely used for quantification of individual complement components, its activated split products and complement regulators. When using well-defined antibodies, ELISA can quantify complement-derived split products next to the native intact protein, reflecting the actual state of complement activation (2, 3, 105). For measuring the specific complement activation products antibodies recognizing split-product-specific neoepitopes, i.e. neoepitopes that only become exposed after cleavage of the native protein, should be used. In addition, detection of split products generated *via* complement-independent bypass routes is possible (106).

Next to ELISA, a more recent technique being developed for the quantification of individual complement components and their split products is (in most cases bead-based) multiplex assays. Such assay is, in principle, a combination of immunoassays thereby able to analyze multiple analytes at the same time. Next to the advantages of saving time and sample volume, these assays provide a comprehensive overview of the patient’s complement activation status in a single run (107). Currently, the specificity of these commercial bead based multiplex complement assays is still under debate (107, 108) which argues thorough validation of its use in comparison to existing assay formats.

## Methods

### Systematic literature review

Our systematic review procedure was adopted from the Preferred Reporting Items for Systematic Reviews and Meta-Analyses (PRISMA) guidelines (109). Structured literature searches using the Pubmed/MEDLINE database were performed in February 2022. Search terms included the medical subject headings (MeSH) terms for: “Complement C3” OR “Complement C3a” OR “Complement C5” OR “Complement C5a” OR “Complement Membrane Attack Complex” AND “Complement activation” to retrieve all studies assessing these complement components. **Supplementary Dataset 1** lists all keywords captured by the MeSH terms used. Next, titles and abstracts were screened by two independent researchers (ET and RB) to determine if the study met the inclusion criteria (see below). Studies potentially of interest were selected and full text papers were retrieved for in-depth review.

#### Inclusion criteria

All studies that assessed the complement components C3, C5 and/or their split products C3a and C5a and/or sTCC (MAC, sC5b-9) in human blood samples (serum and plasma) that were published in peer-reviewed journals between February 2017 and February 2022 were included.

#### Exclusion criteria

The following studies were excluded: not assessing complement components C3, C5 and/or their split products and/or sTCC in human blood samples, animal studies, *ex-vivo* stimulation studies, reviews or editorials, studies in languages other than English and no full text available. The selection process was performed by two authors (ET and RB), based on titles, abstracts, and subsequently full text papers.

#### Quality assessment

Subsequently, studies that passed the inclusion criteria were categorized according to sample type and techniques used for complement assessment. Studies that were identified as using the correct sample type for their complement measurements (according to the guidelines addressed above and in **Figure 2**) were scored regarding overall quality standards for performing research, using the following criteria (110):

1. Did the study provide sufficient information regarding the experimental design and methods (e.g. appropriate method/approach to investigate study aim, clear which analyses were performed in which experimental groups/samples)?
2. Were the baseline characteristics sex and age checked and matched in the control cohorts used for the study?
3. Was sample collection, storage and handling performed according to the requirements for assessment of complement components as outlined in this paper?
4. Were measurements performed in a blinded fashion?

Using these criteria, the nominated studies were awarded a score ranging from 0 to 4, 1 point per met criterion.

### Assessment of C3, C5, their split products and sTCC in human samples to demonstrate the effect of (in)correct pre-analytical sample handling, antigenicity and selecting the appropriate technique

Three experiments were performed to illustrate the importance of correct pre-analytical sample handling and complement assessment.

In the first experiment, we tested the effect of artificial complement activation in the tube induced by inappropriate pre-analytical sample handling. The components C3c and sTCC were measured in complement-preserved serum samples (n=5) and in plasma samples anticoagulated either with citrate, heparin, EDTA 10 mM (normal EDTA blood collection tube, no extra EDTA added) and EDTA ≥20 mM (normal EDTA blood collection tube with extra EDTA added) (n=5 for each anticoagulant). All samples used for this experiment were derived from healthy controls (HC). Plasma samples were put on ice immediately after collection and processed within 1 h (centrifuged for 10 min., 2000x*g* at 4°C and stored at -80°C until further use). Complement-preserved serum samples from were collected aseptically and processed within 1 h (clotted at room RT 30-45 min., centrifuged for 10 min., 2000x*g* at 4°C and stored at -80°C until further use). Aliquots for all samples were thawed and incubated either at RT or on ice for 10 min, 1 h, 2 h and 16 h before measurements. Subsequently, C3c and sTCC levels were measured by commercially available solid-phase ELISA assays according to the manufacturer’s instructions (Cat# HK368 (C3c), HK328 (sTCC), Hycult Biotech, Uden, The Netherlands). Assays are specific for these markers and do not detect other activation products (e.g. C3b, iC3b, C3d) or components not integrated in the TCC. The levels measured in the samples incubated for 10 min were used as a baseline and were set to 100%. All other timepoints were compared to baseline.

Secondly, we assessed C3 concentrations in EDTA-plasma derived from HC (n=6) using two in-house assay designs. These assays did not differ from each other, except for the capture antibody to test the effect of (differences in) antigenicity between antibodies. Samples were collected and handled as described above. Samples were thawed on ice and kept on ice during pre-analytical sample handling. Two distinct capture antibodies were used, recognizing a different epitope in the C3 protein, i.e., the monoclonal antibody clone 3E7 (cat# HM2286) *versus* the monoclonal antibody 1H8 (cat# HM2287; Hycult biotech, Uden, The Netherlands). Antibody concentrations and all other reagents and buffers (HK355; Hycult biotech, Uden, The Netherlands), as well as the calibrator (human native C3; cat#113, Complement Technology, TX, USA) were the same between the two assay designs and assays were performed by the same operator on the same day. C3 concentrations were measured using both setups in parallel according to manufacturer instructions. Briefly, microtiter wells (Nunc maxisorp cat# 468667, Thermo Fisher Scientific, Waltham, MA, USA) were coated either with clone 3E7 or 1H8 at 4°C overnight (5 µg/ml) in 1x PBS. After blocking with 1x PBS, 1% BSA for 1 – 1.5 hours, wells were washed four times using 1x PBS, 0.05% Tween 20. Calibrator was added to the wells in a 2x serial dilution ranging from 500 to 7.8 ng/ml in dilution buffer (DB; 1x PBS, 0.1% BSA). EDTA-plasma samples were added to the wells in a serial dilution ranging from 1:500 to 1:64000, also in DB. Both calibrator and samples were incubated 1 h at RT. For both assays the same detection antibody was used (monoclonal antibody clone 474; cat# HM2073, biotinylated; Hycult biotech, Uden, The Netherlands). After washing, wells were incubated with detection antibody (0.1 µg/ml in DB) for 1 h at RT. Again, wells were washed and tetramethylbenzidine substrate (TMB; K-blue aqueous; Neogen, Scotland, UK) was added starting an enzymatic reaction that produced a colored product that can be measured. Reaction was stopped after 30 min. by adding oxalic acid and the absorbance at 450 nm was measured.

The third experiment focused on choosing the appropriate marker, accompanied by the correct technique and the added value of quantifying both native complement proteins and their split products in parallel in patients with a severe acute respiratory syndrome coronavirus-2 (SARS-CoV-2) infection. Results were adapted from a previously reported study (66). The complement markers C3, C3a, C3c, C5 and sTCC were assessed in EDTA-plasma samples from patients with PCR-proven or clinically presumed Corona Virus Disease 2019 (COVID-19) admitted to the intensive care unit (ICU) and in HC. Also here, EDTA-plasma samples were collected, processed, and handled as described above. Complement measurements were performed using commercially available solid-phase ELISA assays according to the manufacturer’s instructions [Cat# HK355 (C3), HK354 (C3a), HK368 (C3c), HK390 (C5), HK328 (TCC), Hycult Biotech, Uden, The Netherlands].

### Statistics

Continuous data were presented as the mean ± SEM following criteria for normal distribution. Data were analyzed using a (paired) student t-test, ANOVA or Mann-Whitney test where appropriate (Graphpad Prism 8.4.2, San Diego, CA, USA). A p-value <0.05 was considered significant.

### Ethics statement

The study protocol was approved by the local ethics committee (CMO 2020 6344 and CMO 2016 2963) and performed in accordance with the latest version of the declaration of Helsinki and guidelines for good clinical practice (GCP).

## Results

### Systematic literature review

A systematic literature review was conducted to provide a comprehensive overview of the methods and sample types used for the assessment of complement markers during the period February 2017 – February 2022. Subsequently, we assessed whether the identified studies performed their complement analyses in a reliable manner regarding techniques and sample types. We focused on the key complement markers C3, C5 and their split products C3a and C5a, and sTCC.

**Figure 3:**
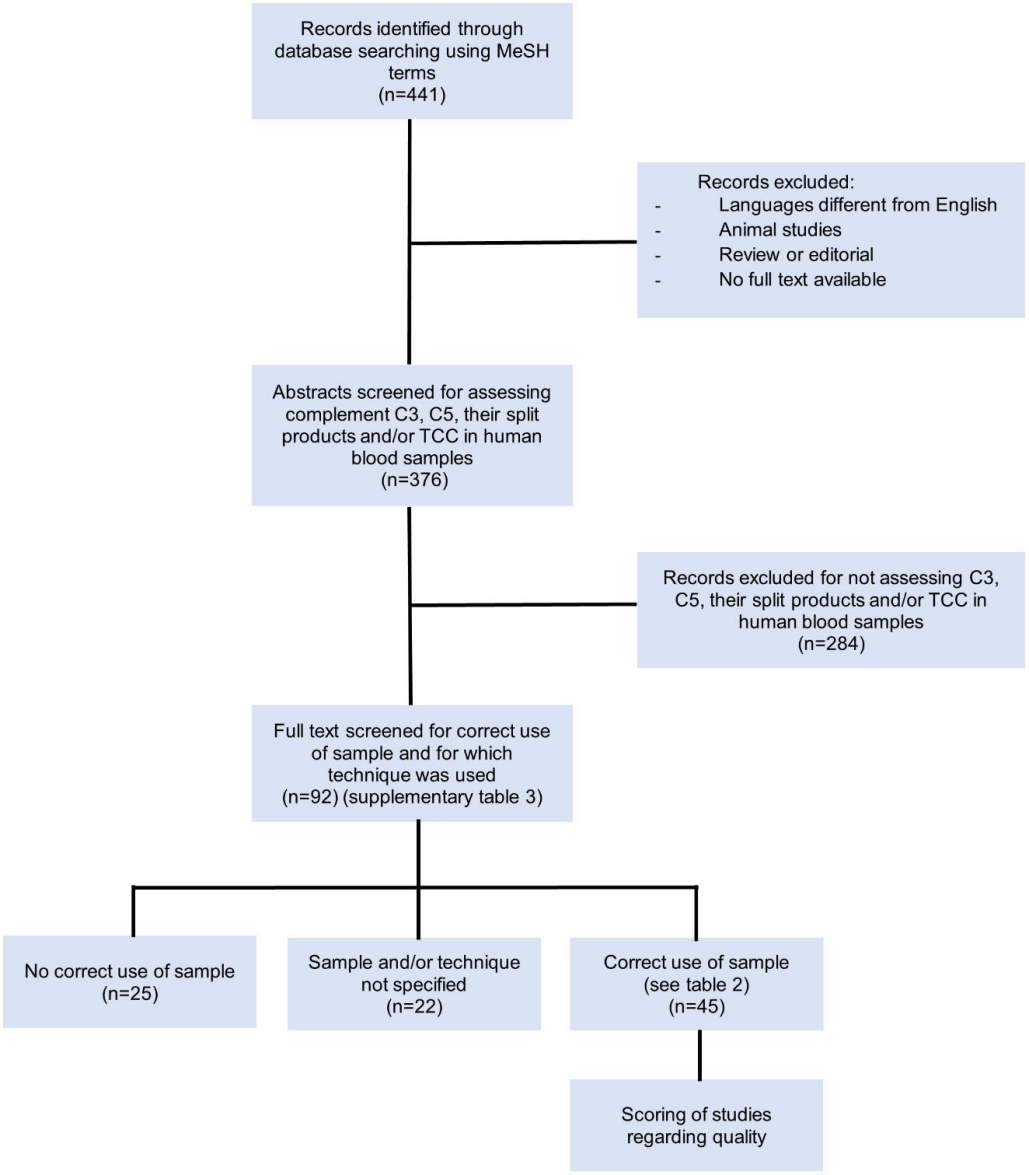
Flowchart of the systematic literature search selection process.

In total, 441 records were identified through database searching using the indicated MeSH terms (**Figure 3**). After excluding non-English papers, animal studies, reviews/editorials and records of which no full text was available, 376 records remained (**Supplementary Dataset 2**). These remaining abstracts were screened based on whether levels of the complement components C3, C5 and/or their split products and/or TCC were assessed in human blood. Studies performing *ex-vivo* stimulation experiments, assessing complement deposition in tissues or using cultured cells were excluded. A total of 92 articles were selected for full text analysis. Study characteristics such as aim of the study, experimental set-up and used techniques were recorded (**Supplementary Table 1**).

**Figure 4:**
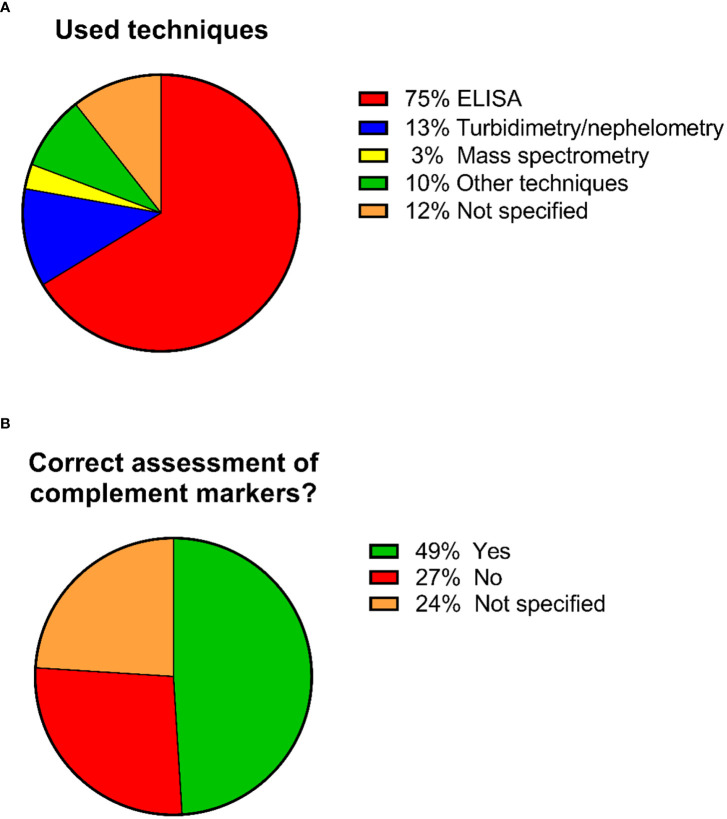
Pie charts showing the percentages regarding the used techniques and whether the correct sample types were used for complement assessment. A total of 92 studies were included in the analysis. **(A)** Percentages regarding the used techniques. Several studies used more than 1 technique for their measurements, hence the number of performed analyses is higher than the total number of the identified studies. **(B)** Percentages of studies that used/did not used the correct sample type for complement assessment.

Regarding the techniques used for measuring complement components in these 92 studies, 69 studies used ELISA (75%), 12 studies (13%) used turbidimetry/nephelometry and three studies (3%) used mass spectrometry. Nine studies (10%) used other techniques and 11 studies (12%) did not specify the technique that was used (**Figure 4**). Several studies used more than one technique for their measurements, hence the number of performed analyses is higher than the total number of the identified studies.

These 92 identified studies were assessed on whether complement measurements were performed correctly and reliably concerning sample type, pre-analytical sample handling and techniques with regard to the complement components being investigated. This resulted in the identification of three distinct groups (**Figure 4**; **Supplementary Table 1**):

1. Studies identified as using the correct sample type for assessing complement activation products (mostly EDTA-plasma) (n=45; 49%) (**Table 2**).
2. Studies identified as not using the correct sample type (mostly serum) (n=25; 27%).
3. Studies that did not specify the sample matrix or the techniques used for measuring complement (n=22; 24%).

**Table 2:** Overview of the 45 selected studies that assessed complement components *ex-vivo* in the past 5 years (2017-2022).

							Scoring criteria					
Author	PMID	Aim of the study	Groups analyzed	Complement markers	Sample matrix	Technique used for analysis	1	2	3	4	Total	Ref.
Witczak BJ	34759922	Investigate posttransplant complement activation in long-term kidney graft and survival in kidney transplant recipients	Kidney transplant recipients	TCC	EDTA-plasma	ELISA	1	1	1	–	3	(98)
Liu M	34671343	Investigate complement proteins as early-pregnancy predictors and potential diagnostic markers of preeclampsia	Healthy and preeclampsia pregnant women before delivery	C3a, C5a	EDTA-plasma	ELISA	1	1	1	–	3	(60)
Pache F	34464830	Investigate C3 and C4 levels in AQP4-IgG+ neuromyelitis optica spectrum disorder, myelin oligodendrocyte glycoprotein antibody-associated disease and multiple sclerosis patients	MOGAD, MS and HC	C3	EDTA-plasma	Immunoturbidimetry assay	1	1	1	–	3	(61)
Michels MAHM	34456924	Assess CP convertase activity in C3G and IC-MPGN (immune complex-mediated membranoproliferative glomerulonephritis) patients	C3G, IC-MPGN patients, HC	C5, TCC	EDTA-plasma	ELISA	1	1	1	–	3	(62)
Nilsson PH	34380648	Characterize human whole blood ex vivo model based on the GPRP peptide for anticoagulation. Examine its utility to assess the effect of thrombin on complement activation	GPRP- and lepirudin-anticoagulated plasma	C3, C5, C5a, TCC	GPRP- and lepirudin-anticoagulated plasma	ELISA	1	1	1	–	3	(102)
Prens LM	34252397	Evaluate systemic complement activation in patients with hidradenitis suppurativa (HS)	HS patients and HC	C3, C5a, TCC	EDTA-plasma	ELISA, Radial immunodiffusion technique	1	1	1	–	3	(63)
Chiu YL	34177889	Investigate alternative pathway activation and disease activity in IgA nephropathy (IgAN)	IgAN and HC	C5a	EDTA-plasma	ELISA	1	1	1	–	3	(64)
Dhooge PPA	34170959	Assess complement activation in Stargardt disease (STGD1) patients	STGD1 patients and controls	C3	EDTA-plasma	ELISA	1	1	1	–	3	(65)
Sinkovits G	33841446	Investigate complement activation in COVID-19	COVID-19 severity groups	C3a, TCC	EDTA-plasma	ELISA	1	1	1	–	3	(66)
Rognes IN	33832430	Describe complement activation in trauma patients from admission to 10 days after injury	Trauma patients	TCC	EDTA-plasma	ELISA	1	1	1	–	3	(67)
Milosevits G	33549818	Study role complement in infusion reactions (IRs) in pediatric patients treated with Abelcet	Pediatric patients treated with Abelcet	C3a	EDTA-plasma	ELISA	1	1	1	–	3	(93)
De Nooijer AH	33038254	Investigate complement activation in COVID-19	COVID-19 and Sepsis patient vs. controls	C3a, C3c, TCC	EDTA-plasma	ELISA	1	1	1	–	3	(59)
Mastellos DC	32961333	Compare eculizumab with compstatin-based C3-targeted drug candidate AMY-101 in COVID-19	COVID-19 patients treated with Eculizumab *vs.* AMY-101	C3, C3a, TCC	EDTA-plasma	ELISA, nephelometric after PEG precipitation (C3dg)	1	1	1	–	3	(92)
Troldborg A	32941885	Characterization of mAb for C3dg and development of time-resolved immunoassay	The SLE patients and controls	C3dg	EDTA-plasma	ELISA	1	1	1	–	3	(100)
Halkjær L	32431705	Establishing an assay for measuring complement activation at C3 level	SLE patients	C3dg	EDTA, citrate, heparin plasma, serum	ELISA	1	1	1	–	3	(99)
Denzinger M	32281172	Activation of complement at the interface of wound dressings	Healthy controls	TCC	EDTA-plasma	Not specified	–	1	1	–	2	(69)
Grinde D	32152940	Evaluate complement in DiGeorge syndrome	DiGeorge syndrome patients	TCC	EDTA-plasma	ELISA	1	1	1	–	3	(70)
Kristensen MK	32082310	Assess complement in necrotizing soft-tissue infection (NSTI) patients	NSTI patients vs. HC	C3, C3bc, TCC	EDTA-plasma	ELISA	1	1	1	–	3	(71)
Chauvet S	32034108	Investigate complement activation in children with acute postinfectious GN	Children with acute postinfectious GN vs. children with C3G and hypocomplementemia	C3, TCC	EDTA-plasma	ELISA	1	1	1	–	3	(72)
Abe T	31917735	Investigate complement in sepsis, including disseminated intravascular coagulation (DIC)	Sepsis patients with and without DIC.	TCC	EDTA-plasma	ELISA	1	1	1	–	3	(73)
Sartain S	31774252	Asses AP activation in Transplant-associated thrombotic microangiopathy (TA-TMA) after hematopoietic stem cell transplantation (HSCT)	HSCT patients with TA-TMA and without TA-TMA	C3a, C5a, TCC	EDTA-plasma	ELISA	1	1	1	–	3	(96)
Tjernberg AR	31691001	Explore complement response to Streptococcus pneumoniae in celiac disease (CD)	CD vs HC	C3a, TCC	EDTA-plasma	ELISA	1	1	1	–	3	(74)
Faria B	31497011	Investigate effect of intravenous iron on complement activation in-vivo	Non-dialysis vs. dialysis patients	TCC	EDTA-plasma	ELISA	1	1	1	–	3	(91)
Scambi C	31479579	Investigate complement activation in women with antiphospholipid syndrome (APS)	APS non‐pregnant patients and pregnant APS women	C5a, TCC	Citrate-plasma	ELISA	1	1	1	–	3	(75)
Schein TN	31461782	Examine TCC levels in HIV patients with poor immune reconstitution	HIV-infected patients and HC	TCC	EDTA-plasma	ELISA	1	1	1	–	3	(76)
Zhang MF	31399080	Asses complement in patients with primary membranous nephropathy (pMN)	Patients with biopsy-proven pMN vs. HC	C3a, C5a, TCC	EDTA-plasma	ELISA	1	1	1	–	3	(77)
Vercauteren KOA	31379459	Assess preanalytical stability of widely used tests to screen complement.	HC	C3d, C3c	EDTA-plasma	ELISA, nephelometric and immunofixation after PEG precipitation	1	1	1	–	3	(104)
Gavriilaki E	31266080	Evaluate complement, endothelial damage, and activation of coagulation in patients with transplant-associated thrombotic microangiopathy (TA-TMA)	Patients with TA-TMA and control HCT recipients without TA-TMA	TCC	EDTA-plasma	ELISA	1	1	1	–	3	(78)
Lynch AM	31203676	Examine complement activation in patients with age-related macular degeneration (AMD), geographic atrophy, and neovascular AMD	AMD patients vs. cataract controls	C3a, TCC	EDTA-plasma	ELISA	1	1	1	–	3	(79)
Elvington M	31019515	Development of an ELISA to quantitate C3(H2O)	Inflammatory-driven diseases vs. HC	C3(H20)	Serum and plasma	ELISA	1	1	1	–	3	(101)
Høiland II	30920726	Investigate TCC and future risk of incident venous thromboembolism (VTE)	VTE patiens vs HC	TCC	EDTA-plasma	ELISA	1	1	1	–	3	(80)
Mansur S	30889724	Study the polyethersulphone (PES) membrane blended with polyurethane (PU) for blood purification applications	Plasma with and without PU	C3a	Citrate-plasma	ELISA	1	1	1	–	3	(103)
Burwick RM	30399106	Evaluate C5b-9 in blood and urine in preeclampsia	Women with preeclampsia with severe features vs. control	TCC	EDTA-plasma	ELISA	1	1	1	–	3	(81)
Rodríguez E	30380547	Investigate complement in AKI pathogenesis.	AKI patients vs. controls	TCC	EDTA-plasma	ELISA	1	1	1	–	3	(82)
Chauvet S	30333829	Investigate complement in patients with C3G with monoclonal immunoglobulin (MIg-C3G)	MIg-C3G patients	C3, TCC	EDTA-plasma	ELISA, nephelometry	1	1	1	–	3	(83)
Bavia L	29908956	Investigate C3d and sC5b9 as biomarkers for myocardial injury	Acute myocardial infarction (AMI) patients vs. controls	C3d, TCC	EDTA-plasma	Rocket immunoelectrophoresis and ELISA	1	1	1	–	3	(84)
Kanni T	29405257	Explore complement activation in hidradenitis suppurativa (HS)	HS patients vs. HC	C5a, TCC	Heparin-plasma	ELISA	1	1	1	–	3	(85)
Siljan WW	29171871	Examine TCC in community-acquired pneumonia (CAP).	CAP patients severity groups	TCC	EDTA-plasma	ELISA	1	1	1	–	3	(86)
Trendelenburg M	29064269	Explore role complement in long-term survival in acute heart failure (AHF)	Patients with AHF vs. HC	C3a, TCC	EDTA-plasma	ELISA	1	1	1	–	3	(87)
Qi J	28801815	Asses complement in thrombotic microangiopathy after allogeneic stem cell transplantation (Transplantation-associated thrombotic microangiopathy; TA-TMA)	TA-TMA patients and patients without TA-TMA)	C3b, TCC	EDTA-plasma	ELISA	1	1	1	–	3	(95)
Togarsimalemath SK	28729035	Describe genetic rearrangements involving multiple CFHR genes leading to a CFHR1-R5 hybrid protein	C3G patients	C3, TCC	EDTA-plasma	ELISA	1	1	1	–	3	(88)
Suffritti C	28707730	Evaluate complement activation in acute episode of congestive heart failure (CHF)	CHF patients	C3, TCC	EDTA-plasma	Radial immunodiffusion and ELISA	1	1	1	–	3	(89)
Grosso G	28669410	Investigate TAFI and TAFIa, complement activation, fibrin clot permeability and fibrinolytic function in antiphospholipid syndrome (APS)	APS patients vs. HC	C5a	Citrate-plasma	ELISA	1	1	1	–	3	(90)
Nilsson PH	28610663	Investigation of a C5a neoepitope exposed on C5 after binding to eculizumab *in-vivo*	Sample pre and post eculizumab treatment	C5a	EDTA-plasma	ELISA	1	1	1	–	3	(94)
Wehling C	27784126	Monitoring of complement activation and eculizumab in complement-mediated renal disorders	aHUS, C3G and ab-mediated renal graft rejection (AMR) patients treated with eculizumab	C3, C3d, C5a, TCC	EDTA-plasma	ELISA	1	1	1	–	3	(97)

#### Studies identified as using the correct sample type for assessing complement activation products

For the 45 studies identified as using the correct sample type (mostly EDTA-plasma), ELISA was the method of choice (n=43; 96%), sometimes supplemented with other techniques such as nephelometry or rocket immuno-electrophoresis (n=6; 7%). In this first group, roughly two categories could be identified: studies investigating complement in health and disease (n=39; 87%) and studies with a technical aim such as development or improvement of new tools and assays (n=6; 13%). Regarding studies investigating complement in health and disease, 31 studies assessed complement markers in patients compared to controls in a wide range of infectious, autoimmune, inflammatory and inflammatory-associated diseases or conditions (66, 111–140). A total of 8 studies focused on complement regulation and activation after treatment in patients (141–148). Regarding the technical studies, 3 studies described the development of new tools or assays (149–151) and 3 studies focused on the development/improvement of new methods or procedures (152–154) (**Table 2**). Next, these 45 studies were scored regarding overall quality standards according to the criteria listed in the methods section. This resulted in a total score ranging from 0 to 4. All studies but one (score=2) reached a good score of 3, indicating that all the criteria were met except that analyses were not performed in a blinded fashion or that this was not mentioned (**Table 2**).

#### Studies identified as not using the correct sample type

A total of 25 studies (27%) were identified as not using the correct sample type for the complement measurements (**Figure 4**; **Supplementary Table 1**). Most of these studies used serum samples for measuring individual complement components and their split products. This group also showed a larger variety in techniques used for complement analysis when compared to the group using the correct sample type. ELISA was used in 13 studies (52%), turbidimetry/nephelometry in 7 studies (28%), mass spectrometry in 2 studies (8%) and 5 studies (20%) used other techniques. A total of 5 studies (20%) did not specify the used techniques. Once again, several studies used more than one technique for their measurements, hence the number of performed analyses is higher than the total number of the identified studies.

#### Studies that did not specify the sample matrix or the techniques used for measuring complement

The last group of 22 studies (24%) did not specify which sample type was used for their measurements. For 6 out of these 22 studies (27%), it is unclear whether serum or plasma was used. The remaining 16 studies (73%) used plasma samples for their measurements but did not specify which anticoagulant tubes were used. Regarding the techniques, 12 studies (55%) reported using ELISA, one study (5%), mass spectrometry and one study (5%) nephelometry. For the remaining eight studies (36%), it is unclear which technology was applied. In addition, little information was provided regarding pre-analytical sample handling and storage (**Figure 4**; **Supplementary Table 1**).

Overall, from the 92 selected studies for in-depth analyses, 45 studies (49%) were identified as using the correct sample type for their complement measurements. A total of 47 studies (51%) did not use the correct sample type or it was not clear which sample type was used.

### Assessment of C3, C5, their split products and sTCC in human samples to demonstrate the effect of (in)correct pre-analytical sample handling, antigenicity and selecting the appropriate technique

**Figure 5:**
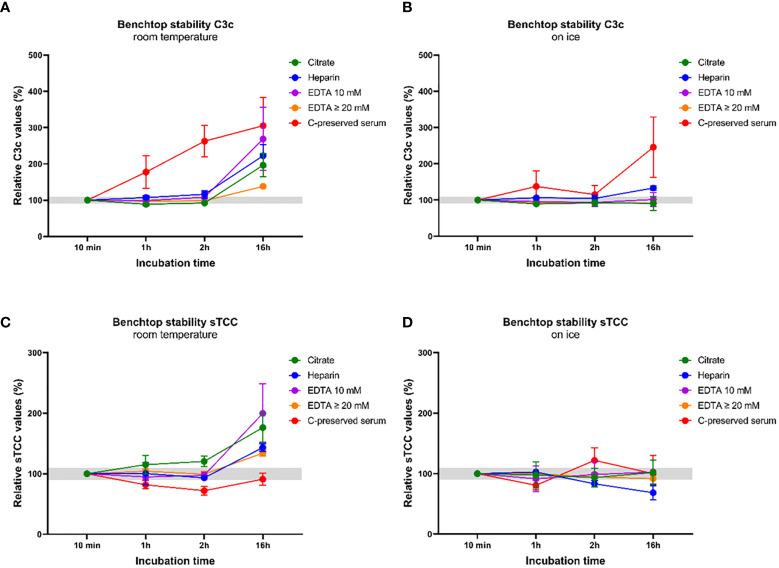
Benchtop stability of the components C3c and soluble TCC in serum samples and in plasma samples with different anticoagulants. C3c and sTCC levels were measured in complement-preserved serum samples (n=5) and in plasma samples anticoagulated either with citrate, heparin, EDTA 10 mM and EDTA ≥20 mM (n=5 for each anticoagulant). All samples were thawed and incubated at RT or on ice for 10 min, 1h, 2h and 16h before measurements. **(A)** C3c levels measured after incubation at RT and **(B)** on ice. **(C)** sTCC levels measured after incubation at RT and **(D)** on ice. Samples were analyzed using a serial dilution ranging from 1:50 to 1:400 and data are represented as mean ± SEM. Deviation from baseline ≤10% is highlighted in grey.

To demonstrate the effect of artificial complement activation in the tube induced by inappropriate pre-analytical sample handling, we measured C3c and sTCC concentrations in complement-preserved serum samples and in plasma samples anticoagulated either with citrate, heparin, EDTA 10 mM and EDTA ≥20 mM. Samples were incubated at RT or on ice for 10 min, 1 h, 2 h and 16 h before measurements. C3c concentrations measured in all plasma samples at RT did not significantly differ from baseline concentrations (10 min) when measured after 1h and 2h. However, both C3c concentrations and the degree of variation between samples increased when plasma samples were measured after 16 h at RT (but this difference did not reach statistical significance due to large variation and low number of samples) (**Figure 5A**). In contrast, a strong increase in C3c concentrations was observed in complement-preserved serum measured after 1 h and 2 h at RT when compared to baseline and the difference reached statistical significance after 2 h (36342 ± 2685 ng/ml *versus* 15201 ± 2760 ng/ml; *p*=0.0005). Relative C3c values in serum increased 2.0 to 5.4-fold after 16 h when compared to baseline (**Figure 5A**). When plasma samples were kept on ice, no differences in C3c concentrations or much variation between samples was observed after 1 h, 2 h and 16 h (**Figure 5B**). Also for complement-preserved serum on ice, C3c concentrations did not differ at 1 h and 2 h but after 16 h relative C3c values ranged from 0.69 (decrease) to 4.3-fold when compared to baseline (**Figure 5B**). For sTCC, levels show the same pattern as observed for C3c. For plasma samples, sTCC concentrations at RT did not differ at 1 h and 2 h but after 16 h, both concentrations and degree of variation between samples increased (again this increase did not reach statistical significance due to large variation and low number of samples) (**Figure 5C**). Of note, sTCC concentrations in complement-preserved serum samples showed a different pattern as observed for C3c. Concentrations tended to be lower at 1 h, 2 h and 16 h at RT but this was not statistically significant (**Figure 5C**). When samples were kept on ice, all plasma samples showed no differences in sTCC concentrations at 1 h, 2 h and 16 h when compared to baseline (**Figure 5D**). Concentrations of sTCC in complement-preserved serum samples on ice tended to fluctuate between timepoints but this did not reach statistical significance (**Figure 5D**). Overall, EDTA-plasma samples showed most stable results regarding C3c and sTCC levels overtime for both RT and on ice. In addition, less variation in concentrations was observed between samples anticoagulated with 20 mM EDTA when compared to 10 mM EDTA (**Figures 5A–D**).

**Figure 6:**
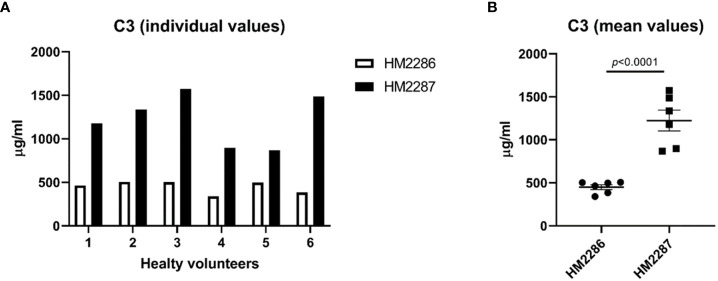
C3 EDTA-plasma concentrations in healthy individuals measured using two different assay designs. EDTA-plasma samples were analyzed in duplicates and data are represented as **(A)** individual values and **(B)** mean ± SEM.

To illustrate the effect of antibodies that differ in antigenicity, we quantified C3 concentrations in EDTA-plasma samples from healthy controls using two ELISA setups capturing C3 at two different epitopes (**Figures 6A, B**). Mean C3 values were 2.7-fold higher in the assay in which HM2287 was used as capturing antibody compared to the assay in which HM2286 was used (1223 ± 120.9 µg/ml *versus* 449 ± 28.6 µg/ml; *p*<0.0001) (**Figure 6B**).

**Figure 7:**
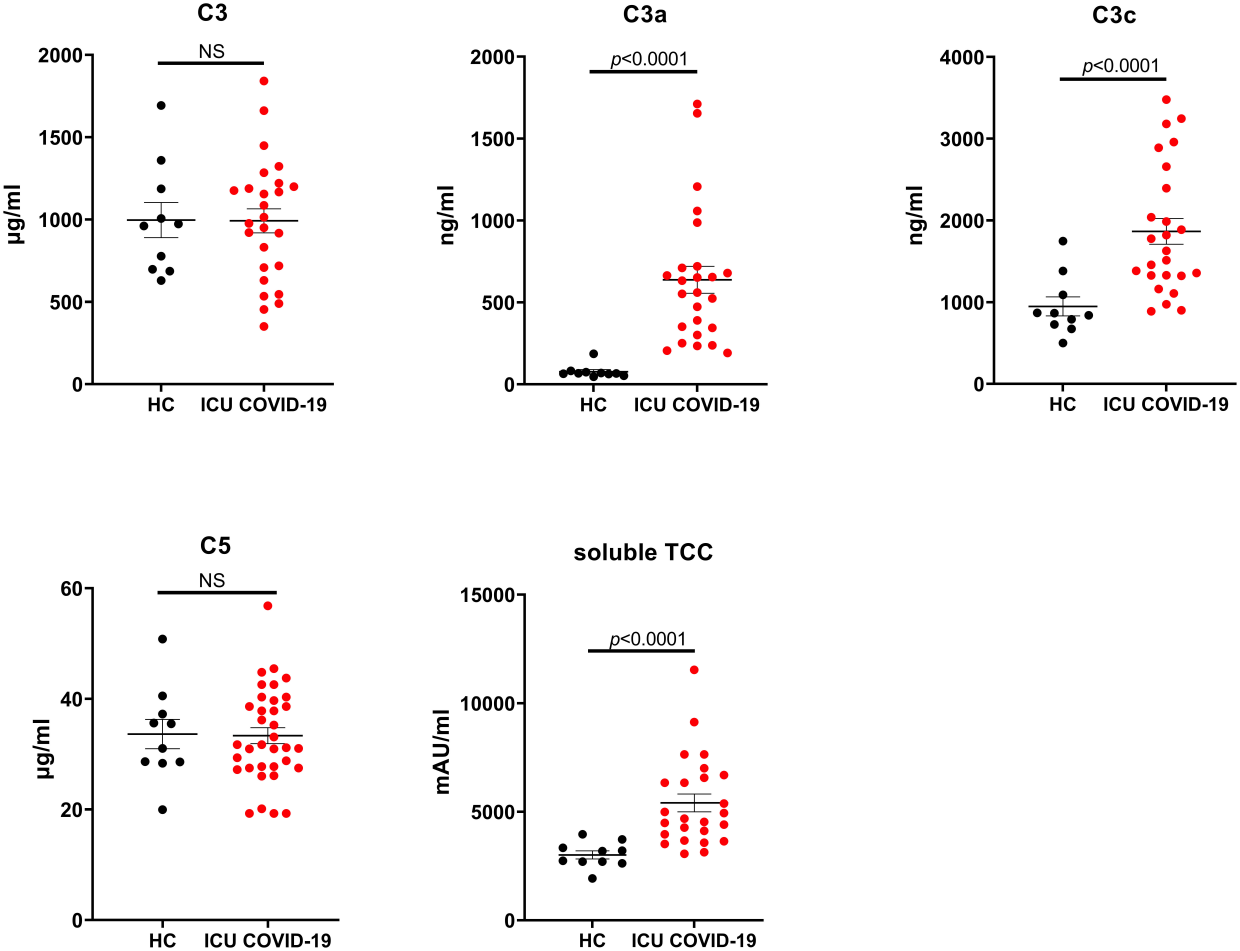
C3 and C5 consumption in COVID-19 patients. EDTA-plasma sample values for the markers C3, C3a, C3c, C5 and soluble TCC. Samples were analyzed using a serial dilution ranging from 1:500 to 1:64000 and data are represented as individual values and mean ± SEM. NS, non-significant; HC, healthy volunteers; ICU, intensive care unit; COVID-19, Corona Virus Disease 2019.

To demonstrate that choosing the correct technique/approach for assessing complement activation is of major importance for correct interpretation of results, we quantified C3, C3a, C3c, C5 and sTCC using ELISA in COVID-19 patients admitted to the ICU. This technique was chosen because of its ability to quantify and distinguish between native complement proteins and their complement-activated split products. Results show that the EDTA-plasma concentrations for the split/activation products C3a, C3c and sTCC were significantly (p<0.0001) increased in ICU patients when compared to HC. In contrast, C3 and C5 concentrations did not differ between these two groups (**Figure 7**) (66).

## Discussion

In this review, we systematically reviewed the available literature in the period 2017-2022 regarding measurements for C3, C5 and/or their split products and/or sTCC in human blood samples and whether these measurements were performed correctly and reliably concerning sample type, pre-analytical sample handling and used techniques and assays.

Out of 376 records, a total of 92 studies were included. From those 92 studies, 45 studies (49%) were identified as using the correct sample type for their measurements. Using our scoring method to assess the overall quality of those 45 studies, most studies showed a total score of 3, indicating good quality. However, it is remarkable that performing a study in a blinded fashion is not standard procedure or is not mentioned. Although beyond the scope of this paper, we would like to stress the importance of basic guidelines and procedures for experiments to assure the quality and integrity of laboratory studies. For instance, ideally samples should be blinded for the operator performing the measurements and experimental groups should be randomly dispersed over the plates. If possible, run all samples on the same plate, especially when samples are paired. This is of course not always possible when the number of samples is too high. In that case, use assays/materials from the same batch to avoid batch-to-batch differences as much as possible. For each separate plate, QC samples with known values should be included on different spots throughout the plate to assess intra- and inter-variability. These procedures for conducting an experiment are often not mentioned.

Twenty-five (27%) studies did not use the correct sample type for their complement measurements. EDTA-plasma is the preferred sample type for measuring individual complement components and their activation products to minimize artificial complement activation. Most of these 25 studies chose serum for their analysis. This does not necessarily mean that the results presented in these 25 studies are unreliable and that these studies are invalid. However, complement components analyses in serum samples can be subjected to artificial complement activation during clotting leading to increased variation in concentrations between samples thereby introducing noise (155). In addition, the identified studies using serum samples did not mention exactly how serum was prepared and what type of collection tubes (with or without clot activator) were used. As also mentioned above, different serum collection tubes may affect complement analyses of the lectin pathway (80, 85). It has been shown that the use of silica as clot activator results in lower ficolin-2 levels and increased ficolin-1 levels (85, 86). In contrast, ficolin-2 depletion does not occur in serum prepared using collection tubes without a clot activator, but the study of Geno and coworkers showed that ficolin-2 inhibitors arise in serum after long-time storage at -80°C (156). These aspects make accurate measurements and identification of differences in concentrations between experimental groups difficult, especially when these differences are subtle. Complement split products are particularly sensitive to false results and measuring them in serum is not valid.

The third group identified consisted of 22 studies (24%) that did not specify which sample type was used for their measurements. Often, these studies also did not report the technology used for their analyses. Also here, this does not necessarily mean that these studies are of lower quality regarding the assessment of complement. However, for these studies it is very difficult to objectively assess and value the results as it is not clear how these results were obtained. Providing information regarding samples, collection tubes, shipment conditions, pre-analytical sample handling and storage, used techniques and methods is key, not only for assessing complement activation but for performing research in general. Providing this information is not only the responsibility of the authors but also of the reviewers during the peer-review process.

### Limitations of the systematic literature review

A systematic review is a powerful analytical tool to collect and analyze data in an objective and standardized way. However, as with all methods, there are limitations. One limitation of our study is that the research question did not include complement components other than the complement markers C3, C5, their split products and TCC. These markers were selected as these are key components of the complement system and, to date, the only approved complement-specific drug targets C5. Another drawback of our study is that systematic searches by using keywords is limited. If MeSH keywords do not match or are not mentioned in titles and/or abstracts, these studies are missed. Next to these ‘practical’ limitations, we are aware that our systematic search does not fully represent pre-analytical sample handling and complement assessment in a daily diagnostic setting. Complement measurements performed solely for diagnostic purposes are not reported in the Pubmed/MEDLINE database and are therefore missed.

### Need for appropriate pre-analytical sample handling and standardized measurement of complement markers

As mentioned in the introduction, there is a high need for appropriate pre-analytical sample handling, storage of samples and standardization of complement testing to be able to compare results between studies and laboratories. Most of the twenty-five studies identified as not using not the correct sample type for their measurements, used serum samples to measure complement activation products.

To provide insight in the effect of artificial complement activation of serum samples in the tube, we measured C3c and sTCC levels in both serum and plasma samples incubated at RT or on ice for several hours. The results clearly show that in serum samples, both C3c levels and variation in levels between samples immediately increases when samples are ‘left on the bench’ at RT for a couple of hours. For plasma samples, this effect is completely absent for the first few hours and only after 16 h, also here some increase in variation and levels is observed. These effects in plasma after 16 h are expected as it is known that these anticoagulants minimize complement activation but not inhibit it completely (1). Incubating samples on ice most certainly slows down artificial complement activation, as the effects on C3c levels in serum are less pronounced when compared to RT. However, also here it is clear that levels and variation between samples changes over time, thereby obscuring the measurements. More or less the same effects were seen for the sTCC measurements, but it was not expected that levels would decrease in serum samples after incubation at RT. An explanation for this is currently lacking, although when also observing the serum sTCC levels incubated on ice, it is clear that levels varied a lot, making measurements difficult to interpret, especially with these low numbers. Regarding the different plasma samples analyzed in this experiment, results show that the concentrations for both C3c and sTCC remained stable for 2 h at RT and on ice in EDTA-plasma. Although concentrations and variations between samples shifted a little after 1 and 2 h in citrate and heparin plasma, they remained relatively stable when compared to baseline. However, it is well-known from literature that EDTA is more effective in inhibiting complement activation when compared to citrate and heparin (83, 157, 158). This holds true not only for benchtop stability but also after freeze-thawing of samples (83), although the magnitude of effects varies between the different complement components. Overall, these results re-confirm that EDTA-plasma is the most stable sample type for measuring complement activation products. Moreover, our results showed that levels in EDTA-plasma samples with extra EDTA (≥20 mM instead of 10 mM) were more stable when compared to plasma samples with no extra EDTA (normal EDTA collection tube). This might argue for the need of adding extra EDTA when samples are collected for the measurement of complement activation products. However, a larger study is needed to confirm these initial results.

Of note, our guidelines for sample handling are more strict than the actual limits that we (and others) have measured in our experimental set-up with healthy control samples (83). However, importantly, conditions in clinical practice are less controlled than in planned experimental settings of blood collection, as was the case in this study. In addition, samples in which there is already (low-rate) complement activation, *in vitro* activation may have faster kinetics, making them even more prone to false results after inappropriate handling.

To illustrate the effect of poorly standardized testing, we measured C3 levels in ETDA-plasma from HCs using two assay setups. Both assays were exact the same except that two distinct capture antibodies were used, recognizing different epitopes in the C3 protein. Recognizing alternative epitopes also means that binding affinity for the C3 antigen differs between the two antibodies. Variation in binding affinity will result in different amounts of antigen that can be detected in an assay. Indeed, results showed a 2.7-fold change in mean C3 levels between the two assay setups in this small cohort of healthy individuals. These results show that using two distinct antibodies, recognizing different epitopes of C3, leads to difficulties in assessing and comparing data and may have consequences for interpretation. Differences can be explained by the conformational changes of C3 during activation (159). Together with accessibility, small changes in conformational epitopes can lead to changes in affinity and antibody kinetics when applied in assays. This indicates the need for assays using antibodies that recognize the same epitope within the antigen being measured, especially in the light that antibody-based assays are among the most-preferred methods for complement assessment. Next to that, assays need to be calibrated against uniform protein standards in order to produce results that can be compared between different testing facilities.

Furthermore, our results show that selecting the correct markers, accompanied by the appropriate technique, is of high importance for measuring complement, especially in a diagnostic setting. Our measurements in COVID-19 patients, using dedicated ELISAs for both the native proteins as well as the split products, clearly showed that complement is activated in COVID-19. Levels for the complement activation markers C3a, C3c and sTCC were increased in patients when compared to controls whereas no differences in levels for the native proteins C3 and C5 were detected. Measuring only C3 and C5 plasma concentrations (and not the split products), as performed in most peripheral hospitals handling routine protocols, would have led to the incorrect conclusion that complement is not activated in these COVID-19 patients. Using nephelometry to measure complement activation would, in this case, not suffice as this technique will not distinguish between native complement proteins and its split products and therefore measure a mixture of C3 and its activation products C3a, C3b, iC3b, C3c and C3d. This would have obscured the outcome. Also using ELISAs for only the unprocessed markers C3 and C5 would not suffice is this case. The combination of measurement of native and activated complement fragments would yield more and earlier insights in unexpected protein levels, deficiencies and could aid to predict the remaining complement-activating potential or aid in staging of complement during disease.

These inconsistencies indicate the high need for well-characterized and standardized assays. This need for standardization and well-defined and characterized assays is well-known within the field. It is therefore that in 2009, the Sub-Committee for the Standardization and Quality Assessment of Complement Measurements (the ‘Complement EQA Group’) was formally recognized and became part of the IUIS (International Union of Immunological Societies) Quality Assessment and Standardization Committee (https://iuis.org/committees/qas/) (2, 82). Next to that, the Complement EQA Group is a standing committee also of the International Complement Society (ICS). The purpose of the EQA group is to facilitate standardized diagnostic complement analysis by developing calibrator materials and test recommendations (2, 82). Initial steps have been made as a serum standard to be used as calibrator for quantitative and functional complement analysis in normal, non-activated human serum (Standard 1; ICS#1) was prepared and evaluated. Next to ‘Standard 1’, also a complement activated standard (Standard 2; ICS#2) was prepared to be used as calibrator in assays for quantifying complement activation products. Both calibrators, together with single patient pathological samples were sent around to participating complement diagnostics laboratories for quality assessment rounds (or ring trials); an inter-laboratory test that allows to evaluate the performance of testing laboratories. Using the calibrators ICS#1 and #2, several complement factors were quantified in parallel in all participating laboratories. The between-laboratory coefficient of variation (CV%) ranged between 0.07 and 0.99 (2, 3, 82). Creating such calibrators with defined amounts of the individual complement components for complement assessment in a diagnostic setting will most certainly improve the quality and consistency of the test results and much progress has been made regarding this. However, as also stated by the Complement EQA Group, other assay components should be used in a standardized matter. For instance, recommendations for sample matrices and advice/descriptions regarding the preferred epitopes in antibody-based assays would most likely improve standardized complement measurements. Next, the EQA Group could provide concentration ranges for complement markers within diseases to be used as reference values for laboratories. This will provide each testing facility the opportunity to set up its own assay without infringing reproducibility in test results between different laboratories.

## Conclusions and future perspective

As the results of our systematic literature review have illustrated, 49% of the identified studies investigating complement activation uses the appropriate sample type for their analysis while 27% of the studies did not. For 24% of the studies, it was not clear what sample type or technique was used. Next, we provided examples that using inappropriate sample types and techniques might lead to misinterpretation of results. Complement biology is an emerging field given the prominent pathogenic involvement in many diseases and its potential for tailored therapeutic intervention. As a result, assessment of complement activation and regulation will become more important in the near future for both research and the daily diagnostic setting. Therefore, it is of utmost importance that complement analysis is performed in a robust and reliable manner. This holds not only for the assessment itself, but also for sample collection, shipment, pre-analytical sample handling, and storage. We underline the importance of awareness regarding these matters and that they may influence the outcome of complement studies.

For the future, we recommend that a uniform protocol is used for standardized sample collection, shipment, pre-analytical handling and storage for both the assessment of complement function as the measurements of individual complement components. Next to that, a more detailed description of the study design is needed regarding used methods and materials (e.g., type of collection tubes). For functional testing, complement-preserved serum (prepared as indicated in **Figure 2**) should be used. To assess individual complement components, EDTA-plasma must be used to minimize artificial complement activation. A first set-up for this uniform protocol is outlined in **Figure 2**. The assessment itself should be performed using standardized assays to produce results that are robust and comparable between diagnostic laboratories. Regarding the assays, standardized calibrators and reagents must be used. Antibody-based assays should preferably use antibodies recognizing the same epitope to minimize the variation in kinetics of the antibodies for the antigen. Standardization of complement analysis is organized and coordinated by the Sub-Committee for the Standardization and Quality Assessment of Complement Measurements (the ‘Complement EQA Group’). We believe that this group should be strongly embedded within and supported by the complement society so that they can have a leading role in providing clear directives for complement measurements.

## Data availability statement

The original contributions presented in the study are included in the article/**Supplementary Material**. Further inquiries can be directed to the corresponding author.

## Author contributions

ET, RB and WB designed and supervised the study. ET, MR and AN performed the experiments. ET analyzed the data and wrote the manuscript. RB, MM, AN, PN and WB critically reviewed the manuscript. All authors discussed and interpreted the results, critically read the article and approved the final version.

## Funding

ET received funding from European Union’s Horizon 2020 research and innovation programme under grant agreement No. 899163 “Screening of inFlammation to enable personalized Medicine” (SciFiMed, https://scifimed.eu/) and under grant agreement No. 860044 “COmplement Regulation and Variations in Opportunistic infectionS” (CORVOS; EU-H2020-MSCA-ITN-EJD; https://www.corvos.eu). RB received funding from the Dutch Kidney foundation (DKF) / Top Consortia for Knowledge and Innovation’s (TKI) & Life Sciences & Health (LSH) joint project “Improved Risk Stratification prior to Kidney Transplantation by Discriminating Pathogenic from Clinically Irrelevant Donor Epitope Specific HLA antibodies in relation to complement activation” (STRIDE.COM) under grant agreement No. DKF PPS09 / TKI-LSH19014-H026.

## Acknowledgments

We thank Alexander Hoischen for his help and advice during this study.

## Conflict of interest

RB, MR, WB and ET are employees of Hycult Biotech b.v.

The remaining authors declare that the research was conducted in the absence of any commercial or financial relationships that could be construed as a potential conflict of interest.

## Publisher’s note

All claims expressed in this article are solely those of the authors and do not necessarily represent those of their affiliated organizations, or those of the publisher, the editors and the reviewers. Any product that may be evaluated in this article, or claim that may be made by its manufacturer, is not guaranteed or endorsed by the publisher.
