# Supplementary material for: Pitfalls in complement analysis: A systematic literature review of assessing complement activation
Source: Front Immunol. 2022 Oct 18;13:1007102. doi: 10.3389/fimmu.2022.1007102 (PMC9623276; doi:10.3389/fimmu.2022.1007102)
Supplement: Supplementary file 1 [file DataSheet_1.docx]

Supplementary Material

# Supplementary Data

**Supplementary dataset 1.**

Keywords captured by the medical subject headings (MeSH) terms used in the systematic literature search

**Complement C3**

- C3, Complement
- Complement 3
- Complement Component 3
- Component 3, Complement
- C3 Complement
- Complement, C3
- Complement C3 Precursor
- C3 Precursor, Complement
- Precursor, Complement C3
- Pro-C3
- Pro C3
- Precursor-Complement 3
- Precursor Complement 3
- Pro-Complement 3
- Pro Complement 3
- C3 Precursor
- Precursor, C3

**Complement C3a**

- C3a, Complement
- Complement 3a
- Complement Component 3a
- Component 3a, Complement
- C3a Complement
- Complement, C3a

**Complement C5**

- C5, Complement
- C5 Complement
- Complement, C5
- Complement 5
- Complement Component 5
- Component 5, Complement
- Complement C5, Precursor
- C5, Precursor Complement
- Precursor Complement C5
- Pro-C5
- Pro C5
- Pro-complement 5
- Pro complement 5
- Precursor C5
- C5, Precursor

**Complement C5a**

- C5a, Complement
- C5a Complement
- Complement, C5a
- Complement Component 5a
- Component 5a, Complement
- Complement 5a

**Complement Membrane Attack Complex**

- C5b-8-poly-C9
- C5b 8 poly C9
- Terminal Complement Complex, TCC
- Complement Complex, Terminal
- Complex, Terminal Complement
- Complement Complex C5b-9
- Complement Complex C5b 9
- Cytolytic Terminal Complement Complex
- Membrane Attack Complex, MAC
- C 5b-9
- C5b-9

**Complement activation**

- Complement Pathway, Alternative
- Complement Pathway, Classical
- Complement Pathway, Mannose-Binding Lectin
- Activation, Complement
- Activations, Complement
- Complement Activations

**Supplementary dataset 2.** Total overview of the 376 records that were identified in the systematic literature search.

1: Fox CR, Parks GD. Complement Inhibitors Vitronectin and Clusterin Are

Recruited from Human Serum to the Surface of Coronavirus OC43-Infected Lung

Cells through Antibody-Dependent Mechanisms. Viruses. 2021 Dec 24;14(1):29. doi:

10.3390/v14010029. PMID: 35062233; PMCID: PMC8780186.

2: Huber S, Massri M, Grasse M, Fleischer V, Kellnerová S, Harpf V, Knabl L,

Knabl L Sr, Heiner T, Kummann M, Neurauter M, Rambach G, Speth C, Würzner R.

Systemic Inflammation and Complement Activation Parameters Predict Clinical

Outcome of Severe SARS-CoV-2 Infections. Viruses. 2021 Nov 26;13(12):2376. doi:

10.3390/v13122376. PMID: 34960645; PMCID: PMC8707937.

3: Witczak BJ, Pischke SE, Reisæter AV, Midtvedt K, Ludviksen JK, Heldal K,

Jenssen T, Hartmann A, Åsberg A, Mollnes TE. Elevated Terminal C5b-9 Complement

Complex 10 Weeks Post Kidney Transplantation Was Associated With Reduced Long-

Term Patient and Kidney Graft Survival. Front Immunol. 2021 Oct 25;12:738927.

doi: 10.3389/fimmu.2021.738927. PMID: 34759922; PMCID: PMC8573334.

4: Liu M, Luo X, Xu Q, Yu H, Gao L, Zhou R, Wang T. Adipsin of the Alternative

Complement Pathway Is a Potential Predictor for Preeclampsia in Early Pregnancy.

Front Immunol. 2021 Oct 4;12:702385. doi: 10.3389/fimmu.2021.702385. PMID:

34671343; PMCID: PMC8521101.

5: Cugno M, Macor P, Giordano M, Manfredi M, Griffini S, Grovetti E, De Maso L,

Mellone S, Valenti L, Prati D, Bonato S, Comi G, Artoni A, Meroni PL, Peyvandi

F. Consumption of complement in a 26-year-old woman with severe thrombotic

thrombocytopenia after ChAdOx1 nCov-19 vaccination. J Autoimmun. 2021

Nov;124:102728. doi: 10.1016/j.jaut.2021.102728. Epub 2021 Sep 27. PMID:

34592707.

6: Kim DM, Kim Y, Seo JW, Lee J, Park U, Ha NY, Koh J, Park H, Lee JW, Ro HJ,

Yun NR, Kim DY, Yoon SH, Na YS, Moon DS, Lim SC, Kim CM, Jeon K, Kang JG, Jang

NY, Jeong H, Kim J, Cheon S, Sohn KM, Moon JY, Kym S, Han SR, Lee MS, Kim HJ,

Park WY, Choi JY, Shin HW, Kim HY, Cho CH, Jeon YK, Kim YS, Cho NH. Enhanced

eosinophil-mediated inflammation associated with antibody and complement-

dependent pneumonic insults in critical COVID-19. Cell Rep. 2021 Oct

5;37(1):109798. doi: 10.1016/j.celrep.2021.109798. Epub 2021 Sep 20. PMID:

34587481; PMCID: PMC8450316.

7: Garam N, Cserhalmi M, Prohászka Z, Szilágyi Á, Veszeli N, Szabó E, Uzonyi B,

Iliás A, Aigner C, Schmidt A, Gaggl M, Sunder-Plassmann G, Bajcsi D, Brunner J,

Dumfarth A, Cejka D, Flaschberger S, Flögelova H, Haris Á, Hartmann Á, Heilos A,

Mueller T, Rusai K, Arbeiter K, Hofer J, Jakab D, Sinkó M, Szigeti E, Bereczki

C, Janko V, Kelen K, Reusz GS, Szabó AJ, Klenk N, Kóbor K, Kojc N,

Knechtelsdorfer M, Laganovic M, Lungu AC, Meglic A, Rus R, Kersnik Levart T,

Macioniene E, Miglinas M, Pawłowska A, Stompór T, Podracka L, Rudnicki M, Mayer

G, Rysava R, Reiterova J, Saraga M, Seeman T, Zieg J, Sládková E, Stajic N,

Szabó T, Capitanescu A, Stancu S, Tisljar M, Galesic K, Tislér A, Vainumäe I,

Windpessl M, Zaoral T, Zlatanova G, Józsi M, Csuka D. FHR-5 Serum Levels and

<i>CFHR5</i> Genetic Variations in Patients With Immune Complex-Mediated

Membranoproliferative Glomerulonephritis and C3-Glomerulopathy. Front Immunol.

2021 Sep 10;12:720183. doi: 10.3389/fimmu.2021.720183. PMID: 34566977; PMCID:

PMC8461307.

8: Loeven MA, Maciej-Hulme ML, Yanginlar C, Hubers MC, Kellenbach E, de Graaf M,

van Kuppevelt TH, Wetzels J, Rabelink TJ, Smith RJH, van der Vlag J. Selective

Binding of Heparin/Heparan Sulfate Oligosaccharides to Factor H and Factor

H-Related Proteins: Therapeutic Potential for C3 Glomerulopathies. Front

Immunol. 2021 Aug 18;12:676662. doi: 10.3389/fimmu.2021.676662. PMID: 34489931;

PMCID: PMC8416517.

9: Li Y, Wang G, Griffin L, Banda NK, Saba LM, Groman EV, Scheinman R, Moghimi

SM, Simberg D. Complement opsonization of nanoparticles: Differences between

humans and preclinical species. J Control Release. 2021 Oct 10;338:548-556. doi:

10.1016/j.jconrel.2021.08.048. Epub 2021 Sep 2. PMID: 34481928; PMCID:

PMC8552414.

10: Pache F, Ringelstein M, Aktas O, Kleiter I, Jarius S, Siebert N, Bellmann-

Strobl J, Paul F, Ruprecht K. C3 and C4 complement levels in AQP4-IgG-positive

NMOSD and in MOGAD. J Neuroimmunol. 2021 Nov 15;360:577699. doi:

10.1016/j.jneuroim.2021.577699. Epub 2021 Aug 24. PMID: 34464830.

11: Michels MAHM, van de Kar NCAJ, van Kraaij SAW, Sarlea SA, Gracchi V, Engels

FAPT, Dorresteijn EM, van der Deure J, Duineveld C, Wetzels JFM, van den Heuvel

LPWJ, Volokhina EB. Different Aspects of Classical Pathway Overactivation in

Patients With C3 Glomerulopathy and Immune Complex-Mediated

Membranoproliferative Glomerulonephritis. Front Immunol. 2021 Aug 11;12:715704.

doi: 10.3389/fimmu.2021.715704. PMID: 34456924; PMCID: PMC8386118.

12: Nilsson PH, Johnson C, Quach QH, Macpherson A, Durrant O, Pischke SE, Fure

H, Landsem A, Bergseth G, Schjalm C, Haugaard-Kedström LM, Huber-Lang M, van den

Elsen J, Brekke OL, Mollnes TE. A Conformational Change of Complement C5 Is

Required for Thrombin-Mediated Cleavage, Revealed by a Novel Ex Vivo Human Whole

Blood Model Preserving Full Thrombin Activity. J Immunol. 2021 Sep

15;207(6):1641-1651. doi: 10.4049/jimmunol.2001471. Epub 2021 Aug 11. PMID:

34380648; PMCID: PMC8428748.

13: Chinchilla B, Fernandez-Godino R. AMD-Like Substrate Causes Epithelial

Mesenchymal Transition in iPSC-Derived Retinal Pigment Epithelial Cells Wild

Type but Not <i>C3</i>-Knockout. Int J Mol Sci. 2021 Jul 30;22(15):8183. doi:

10.3390/ijms22158183. PMID: 34360950; PMCID: PMC8348968.

14: Prens LM, Ardon CB, van Straalen KR, van der Zee HH, Seelen MAJ, Laman JD,

Prens EP, Horváth B, Damman J. No Evident Systemic Terminal Complement Pathway

Activation in Hidradenitis Suppurativa. J Invest Dermatol. 2021

Dec;141(12):2966-2969.e1. doi: 10.1016/j.jid.2021.03.037. Epub 2021 Jul 9. PMID:

34252397.

15: Pollack S, Eisenstein I, Mory A, Paperna T, Ofir A, Baris-Feldman H, Weiss

K, Veszeli N, Csuka D, Shemer R, Glaser F, Prohászka Z, Magen D. A Novel

Homozygous In-Frame Deletion in Complement Factor 3 Underlies Early-Onset

Autosomal Recessive Atypical Hemolytic Uremic Syndrome - Case Report. Front

Immunol. 2021 Jun 24;12:608604. doi: 10.3389/fimmu.2021.608604. PMID: 34248927;

PMCID: PMC8264753.

16: Chen YY, Han SS, Cao Y, Yu XJ, Zhu L, Luo JC, Song WC, Yu F, Mao YH, Zhao

MH. von Willebrand factor variants in C3 glomerulopathy: A Chinese cohort study.

Clin Immunol. 2021 Aug;229:108794. doi: 10.1016/j.clim.2021.108794. Epub 2021

Jul 8. PMID: 34245915.

17: Lam LKM, Reilly JP, Rux AH, Murphy SJ, Kuri-Cervantes L, Weisman AR, Ittner

CAG, Pampena MB, Betts MR, Wherry EJ, Song WC, Lambris JD, Meyer NJ, Cines DB,

Mangalmurti NS. Erythrocytes identify complement activation in patients with

COVID-19. Am J Physiol Lung Cell Mol Physiol. 2021 Aug 1;321(2):L485-L489. doi:

10.1152/ajplung.00231.2021. Epub 2021 Jul 7. PMID: 34231390; PMCID: PMC8384475.

18: Gyapon-Quast F, Goicoechea de Jorge E, Malik T, Wu N, Yu J, Chai W, Feizi T,

Liu Y, Pickering MC. Defining the Glycosaminoglycan Interactions of Complement

Factor H-Related Protein 5. J Immunol. 2021 Jul 15;207(2):534-541. doi:

10.4049/jimmunol.2000072. Epub 2021 Jun 30. PMID: 34193601; PMCID: PMC8313009.

19: Khandelwal S, Barnes A, Rauova L, Sarkar A, Rux AH, Yarovoi SV, Zaitsev SS,

Lambris JD, Myoung SS, Johnson A, Lee GM, Duarte M, Poncz M, Arepally GM, Cines

DB. Complement mediates binding and procoagulant effects of ultralarge HIT

immune complexes. Blood. 2021 Nov 25;138(21):2106-2116. doi:

10.1182/blood.2020009487. PMID: 34189574; PMCID: PMC8617432.

20: Aradottir SS, Kristoffersson AC, Roumenina LT, Bjerre A, Kashioulis P,

Palsson R, Karpman D. Factor D Inhibition Blocks Complement Activation Induced

by Mutant Factor B Associated With Atypical Hemolytic Uremic Syndrome and

Membranoproliferative Glomerulonephritis. Front Immunol. 2021 Jun 10;12:690821.

doi: 10.3389/fimmu.2021.690821. PMID: 34177949; PMCID: PMC8222914.

21: Chiu YL, Lin WC, Shu KH, Fang YW, Chang FC, Chou YH, Wu CF, Chiang WC, Lin

SL, Chen YM, Wu MS. Alternative Complement Pathway Is Activated and Associated

with Galactose-Deficient IgA<sub>1</sub> Antibody in IgA Nephropathy Patients.

Front Immunol. 2021 Jun 10;12:638309. doi: 10.3389/fimmu.2021.638309. PMID:

34177889; PMCID: PMC8223746.

22: Dhooge PPA, Runhart EH, Li CHZ, de Kat Angelino CM, Hoyng CB, van der Molen

RG, den Hollander AI. Systemic complement activation levels in Stargardt

disease. PLoS One. 2021 Jun 25;16(6):e0253716. doi:

10.1371/journal.pone.0253716. PMID: 34170959; PMCID: PMC8232401.

23: Chinchilla B, Foltopoulou P, Fernandez-Godino R. Tick-over-mediated

complement activation is sufficient to cause basal deposit formation in cell-

based models of macular degeneration. J Pathol. 2021 Oct;255(2):120-131. doi:

10.1002/path.5747. Epub 2021 Jul 27. PMID: 34155630.

24: Sultan EY, Rizk DE, Kenawy HI, Hassan R. A small fragment of factor B as a

potential inhibitor of complement alternative pathway activity. Immunobiology.

2021 Jul;226(4):152106. doi: 10.1016/j.imbio.2021.152106. Epub 2021 Jun 16.

PMID: 34147816.

25: Min XY, Liu CF, Cao B, Zhang T, Yang X, Ma N, Wang N, Li K. Human

CD3<sup>+</sup>CD56<sup>+</sup>NKT-like cells express a range of complement

receptors and C3 activation has negative effects on these cell activity and

effector function. Hum Immunol. 2021 Sep;82(9):625-633. doi:

10.1016/j.humimm.2021.06.001. Epub 2021 Jun 13. PMID: 34134908.

26: Cumpelik A, Heja D, Hu Y, Varano G, Ordikhani F, Roberto MP, He Z, Homann D,

Lira SA, Dominguez-Sola D, Heeger PS. Dynamic regulation of B cell complement

signaling is integral to germinal center responses. Nat Immunol. 2021

Jun;22(6):757-768. doi: 10.1038/s41590-021-00926-0. Epub 2021 May 24. PMID:

34031614; PMCID: PMC8297556.

27: Garland DL, Pierce EA, Fernandez-Godino R. Complement C5 is not critical for

the formation of sub-RPE deposits in Efemp1 mutant mice. Sci Rep. 2021 May

17;11(1):10416. doi: 10.1038/s41598-021-89978-8. PMID: 34001980; PMCID:

PMC8128922.

28: Khan A, Shang N, Petukhova L, Zhang J, Shen Y, Hebbring SJ, Moncrieffe H,

Kottyan LC, Namjou-Khales B, Knevel R, Raychaudhuri S, Karlson EW, Harley JB,

Stanaway IB, Crosslin D, Denny JC, Elkind MSV, Gharavi AG, Hripcsak G, Weng C,

Kiryluk K. Medical Records-Based Genetic Studies of the Complement System. J Am

Soc Nephrol. 2021 Aug;32(8):2031-2047. doi: 10.1681/ASN.2020091371. Epub 2021

May 3. PMID: 33941608; PMCID: PMC8455263.

29: Silva de França F, Villas-Boas IM, Cogliati B, Woodruff TM, Reis EDS,

Lambris JD, Tambourgi DV. C5a-C5aR1 Axis Activation Drives Envenomation

Immunopathology by the Snake <i>Naja annulifera</i>. Front Immunol. 2021 Apr

15;12:652242. doi: 10.3389/fimmu.2021.652242. PMID: 33936074; PMCID: PMC8082402.

30: Qiu WQ, Luo S, Ma SA, Saminathan P, Li H, Gunnersen JM, Gelbard HA, Hammond

JW. The Sez6 Family Inhibits Complement by Facilitating Factor I Cleavage of C3b

and Accelerating the Decay of C3 Convertases. Front Immunol. 2021 Apr

15;12:607641. doi: 10.3389/fimmu.2021.607641. PMID: 33936031; PMCID: PMC8081827.

31: Matsuyama T, Tomimatsu T, Mimura K, Yagi K, Kawanishi Y, Kakigano A,

Nakamura H, Endo M, Kimura T. Complement activation by an angiogenic imbalance

leads to systemic vascular endothelial dysfunction: A new proposal for the

pathophysiology of preeclampsia. J Reprod Immunol. 2021 Jun;145:103322. doi:

10.1016/j.jri.2021.103322. Epub 2021 Apr 15. PMID: 33887508.

32: Posch W, Vosper J, Noureen A, Zaderer V, Witting C, Bertacchi G, Gstir R,

Filipek PA, Bonn GK, Huber LA, Bellmann-Weiler R, Lass-Flörl C, Wilflingseder D.

C5aR inhibition of nonimmune cells suppresses inflammation and maintains

epithelial integrity in SARS-CoV-2-infected primary human airway epithelia. J

Allergy Clin Immunol. 2021 Jun;147(6):2083-2097.e6. doi:

10.1016/j.jaci.2021.03.038. Epub 2021 Apr 20. PMID: 33852936; PMCID: PMC8056780.

33: Barragán AF, Quintero-Muñoz E, Quintero-Muñoz D, Rodriguez-Segura PV. C3

Glomerulopathy, a pathology with scarce evidence. A case report. G Ital Nefrol.

2021 Apr 14;38(2):2021-vol2. PMID: 33852227.

34: Radanova M, Roumenina LT, Vasilev V. Detection of Anti-C3b Autoantibodies by

ELISA. Methods Mol Biol. 2021;2227:133-139. doi: 10.1007/978-1-0716-1016-9_13.

PMID: 33847938.

35: Boudhabhay I, Grunenwald A, Roumenina LT. Complement C3 Deposition on

Endothelial Cells Revealed by Flow Cytometry. Methods Mol Biol.

2021;2227:97-105. doi: 10.1007/978-1-0716-1016-9_9. PMID: 33847934.

36: Michels MAHM, van de Kar NCAJ, Volokhina EB, van den Heuvel BLPWJ.

Functional Hemolytic Test for Complement Alternative Pathway Convertase

Activity. Methods Mol Biol. 2021;2227:83-96. doi: 10.1007/978-1-0716-1016-9_8.

PMID: 33847933.

37: Troldborg A, Jensenius JC. C3dg Quantification by PEG Precipitation and or

TRIFMA. Methods Mol Biol. 2021;2227:43-49. doi: 10.1007/978-1-0716-1016-9_4.

PMID: 33847929.

38: Gavriilaki E, Asteris PG, Touloumenidou T, Koravou EE, Koutra M, Papayanni

PG, Karali V, Papalexandri A, Varelas C, Chatzopoulou F, Chatzidimitriou M,

Chatzidimitriou D, Veleni A, Grigoriadis S, Rapti E, Chloros D, Kioumis I,

Kaimakamis E, Bitzani M, Boumpas D, Tsantes A, Sotiropoulos D, Sakellari I,

Kalantzis IG, Parastatidis ST, Koopialipoor M, Cavaleri L, Armaghani DJ,

Papadopoulou A, Brodsky RA, Kokoris S, Anagnostopoulos A. Genetic justification

of severe COVID-19 using a rigorous algorithm. Clin Immunol. 2021

May;226:108726. doi: 10.1016/j.clim.2021.108726. Epub 2021 Apr 13. PMID:

33845193; PMCID: PMC8043057.

39: Sinkovits G, Mező B, Réti M, Müller V, Iványi Z, Gál J, Gopcsa L, Reményi P,

Szathmáry B, Lakatos B, Szlávik J, Bobek I, Prohászka ZZ, Förhécz Z, Csuka D,

Hurler L, Kajdácsi E, Cervenak L, Kiszel P, Masszi T, Vályi-Nagy I, Prohászka Z.

Complement Overactivation and Consumption Predicts In-Hospital Mortality in

SARS-CoV-2 Infection. Front Immunol. 2021 Mar 25;12:663187. doi:

10.3389/fimmu.2021.663187. PMID: 33841446; PMCID: PMC8027327.

40: Rognes IN, Pischke SE, Ottestad W, Røislien J, Berg JP, Johnson C, Eken T,

Mollnes TE. Increased complement activation 3 to 6 h after trauma is a predictor

of prolonged mechanical ventilation and multiple organ dysfunction syndrome: a

prospective observational study. Mol Med. 2021 Apr 8;27(1):35. doi:

10.1186/s10020-021-00286-3. PMID: 33832430; PMCID: PMC8028580.

41: Yan B, Freiwald T, Chauss D, Wang L, West E, Mirabelli C, Zhang CJ, Nichols

EM, Malik N, Gregory R, Bantscheff M, Ghidelli-Disse S, Kolev M, Frum T, Spence

JR, Sexton JZ, Alysandratos KD, Kotton DN, Pittaluga S, Bibby J, Niyonzima N,

Olson MR, Kordasti S, Portilla D, Wobus CE, Laurence A, Lionakis MS, Kemper C,

Afzali B, Kazemian M. SARS-CoV-2 drives JAK1/2-dependent local complement

hyperactivation. Sci Immunol. 2021 Apr 7;6(58):eabg0833. doi:

10.1126/sciimmunol.abg0833. PMID: 33827897; PMCID: PMC8139422.

42: Puy C, Pang J, Reitsma SE, Lorentz CU, Tucker EI, Gailani D, Gruber A, Lupu

F, McCarty OJT. Cross-Talk between the Complement Pathway and the Contact

Activation System of Coagulation: Activated Factor XI Neutralizes Complement

Factor H. J Immunol. 2021 Apr 15;206(8):1784-1792. doi:

10.4049/jimmunol.2000398. PMID: 33811105; PMCID: PMC8030746.

43: Silawal S, Kohl B, Shi J, Schulze-Tanzil G. Complement Regulation in Human

Tenocytes under the Influence of Anaphylatoxin C5a. Int J Mol Sci. 2021 Mar

18;22(6):3105. doi: 10.3390/ijms22063105. PMID: 33803624; PMCID: PMC8003014.

44: Ruffatti A, Tonello M, Macor P, Calligaro A, Del Ross T, Favaro M, Lotti V,

Carletto A, Hoxha A, Biasi D. Markers of complement activation in plasma during

quiescent phases in patients with catastrophic antiphospholipid syndrome. Blood.

2021 May 27;137(21):2989-2992. doi: 10.1182/blood.2020010575. PMID: 33649771.

45: Milosevits G, Mészáros T, Őrfi E, Bakos T, Garami M, Kovács G, Dézsi L,

Hamar P, Győrffy B, Szabó A, Szénási G, Szebeni J. Complement-mediated

hypersensitivity reactions to an amphotericin B-containing lipid complex

(Abelcet) in pediatric patients and anesthetized rats: Benefits of slow

infusion. Nanomedicine. 2021 Jun;34:102366. doi: 10.1016/j.nano.2021.102366.

Epub 2021 Feb 5. PMID: 33549818.

46: Yang P, Neal SE, Buehne KL, Tewkesbury GM, Klingeborn M, Yang YY, Baciu P,

Jaffe GJ. Complement-mediated release of fibroblast growth factor 2 from human

RPE cells. Exp Eye Res. 2021 Mar;204:108471. doi: 10.1016/j.exer.2021.108471.

Epub 2021 Jan 28. PMID: 33516764.

47: Mannes M, Dopler A, Zolk O, Lang SJ, Halbgebauer R, Höchsmann B, Skerra A,

Braun CK, Huber-Lang M, Schrezenmeier H, Schmidt CQ. Complement inhibition at

the level of C3 or C5: mechanistic reasons for ongoing terminal pathway

activity. Blood. 2021 Jan 28;137(4):443-455. doi: 10.1182/blood.2020005959.

PMID: 33507296.

48: Bondza S, Marosan A, Kara S, Lösing J, Peipp M, Nimmerjahn F, Buijs J, Lux

A. Complement-Dependent Activity of CD20-Specific IgG Correlates With Bivalent

Antigen Binding and C1q Binding Strength. Front Immunol. 2021 Jan 11;11:609941.

doi: 10.3389/fimmu.2020.609941. PMID: 33505398; PMCID: PMC7829346.

49: Livson S, Jarva H, Kalliala I, Lokki AI, Heikkinen-Eloranta J, Nieminen P,

Meri S. Activation of the Complement System in the Lower Genital Tract During

Pregnancy and Delivery. Front Immunol. 2021 Jan 11;11:563073. doi:

10.3389/fimmu.2020.563073. PMID: 33505390; PMCID: PMC7829332.

50: Hubens WHG, Beckers HJM, Gorgels TGMF, Webers CAB. Increased ratios of

complement factors C3a to C3 in aqueous humor and serum mark glaucoma

progression. Exp Eye Res. 2021 Mar;204:108460. doi: 10.1016/j.exer.2021.108460.

Epub 2021 Jan 23. PMID: 33493474.

51: Heesterbeek DAC, Muts RM, van Hensbergen VP, de Saint Aulaire P, Wennekes T,

Bardoel BW, van Sorge NM, Rooijakkers SHM. Outer membrane permeabilization by

the membrane attack complex sensitizes Gram-negative bacteria to antimicrobial

proteins in serum and phagocytes. PLoS Pathog. 2021 Jan 22;17(1):e1009227. doi:

10.1371/journal.ppat.1009227. PMID: 33481964; PMCID: PMC7886145.

52: Milling S. From functions to mechanisms of the prototypic complement C5

antibody BB5.1. Immunology. 2020 Oct;161(2):81-82. doi: 10.1111/imm.13261. PMID:

33460089; PMCID: PMC7496775.

53: Song L, Ge T, Li Z, Sun J, Li G, Sun Y, Fang L, Ma YJ, Garred P. Artesunate:

A natural product-based immunomodulator involved in human complement. Biomed

Pharmacother. 2021 Apr;136:111234. doi: 10.1016/j.biopha.2021.111234. Epub 2021

Jan 19. PMID: 33454596.

54: Valenti L, Griffini S, Lamorte G, Grovetti E, Uceda Renteria SC, Malvestiti

F, Scudeller L, Bandera A, Peyvandi F, Prati D, Meroni P, Cugno M. Chromosome 3

cluster rs11385942 variant links complement activation with severe COVID-19. J

Autoimmun. 2021 Feb;117:102595. doi: 10.1016/j.jaut.2021.102595. Epub 2021 Jan

9. Erratum in: J Autoimmun. 2021 Jun;120:102646. PMID: 33453462; PMCID:

PMC7796659.

55: Mommert S, Doenni L, Szudybill P, Zoeller C, Beyer FH, Werfel T. C3a and Its

Receptor C3aR Are Detectable in Normal Human Epidermal Keratinocytes and Are

Differentially Regulated via TLR3 and LL37. J Innate Immun. 2021;13(3):164-178.

doi: 10.1159/000512547. Epub 2021 Jan 14. PMID: 33445177; PMCID: PMC8138155.

56: Zelek WM, Morgan BP. Monoclonal Antibodies Capable of Inhibiting Complement

Downstream of C5 in Multiple Species. Front Immunol. 2020 Dec 10;11:612402. doi:

10.3389/fimmu.2020.612402. PMID: 33424866; PMCID: PMC7793867.

57: Xu X, Zhang C, Denton DT, O'Connell D, Drolet DW, Geisbrecht BV. Inhibition

of the Complement Alternative Pathway by Chemically Modified DNA Aptamers That

Bind with Picomolar Affinity to Factor B. J Immunol. 2021 Feb 15;206(4):861-873.

doi: 10.4049/jimmunol.2001260. Epub 2021 Jan 8. PMID: 33419768; PMCID:

PMC7851746.

58: Ozen A, Kasap N, Vujkovic-Cvijin I, Apps R, Cheung F, Karakoc-Aydiner E,

Akkelle B, Sari S, Tutar E, Ozcay F, Uygun DK, Islek A, Akgun G, Selcuk M, Sezer

OB, Zhang Y, Kutluk G, Topal E, Sayar E, Celikel C, Houwen RHJ, Bingol A, Ogulur

I, Eltan SB, Snow AL, Lake C, Fantoni G, Alba C, Sellers B, Chauvin SD, Dalgard

CL, Harari O, Ni YG, Wang MD, Devalaraja-Narashimha K, Subramanian P, Ergelen R,

Artan R, Guner SN, Dalgic B, Tsang J, Belkaid Y, Ertem D, Baris S, Lenardo MJ.

Broadly effective metabolic and immune recovery with C5 inhibition in CHAPLE

disease. Nat Immunol. 2021 Feb;22(2):128-139. doi: 10.1038/s41590-020-00830-z.

Epub 2021 Jan 4. PMID: 33398182; PMCID: PMC7856263.

59: Detsika MG, Goudevenou K, Geurts AM, Gakiopoulou H, Grapsa E, Lianos EA.

Generation of a novel decay accelerating factor (DAF) knock-out rat model using

clustered regularly-interspaced short palindromic repeats, (CRISPR)/associated

protein 9 (Cas9), genome editing. Transgenic Res. 2021 Feb;30(1):11-21. doi:

10.1007/s11248-020-00222-x. Epub 2021 Jan 2. PMID: 33387103.

60: Efstathiou NE, Moustafa GA, Maidana DE, Konstantinou EK, Notomi S, Barbisan

PRT, Georgakopoulos CD, Miller JW, Vavvas DG. Acadesine suppresses TNF-α induced

complement component 3 (C3), in retinal pigment epithelial (RPE) cells. PLoS

One. 2020 Dec 23;15(12):e0244307. doi: 10.1371/journal.pone.0244307. PMID:

33362238; PMCID: PMC7757886.

61: Wymann S, Dai Y, Nair AG, Cao H, Powers GA, Schnell A, Martin-Roussety G,

Leong D, Simmonds J, Lieu KG, de Souza MJ, Mischnik M, Taylor S, Ow SY, Spycher

M, Butcher RE, Pearse M, Zuercher AW, Baz Morelli A, Panousis C, Wilson MJ, Rowe

T, Hardy MP. A novel soluble complement receptor 1 fragment with enhanced

therapeutic potential. J Biol Chem. 2021 Jan-Jun;296:100200. doi:

10.1074/jbc.RA120.016127. Epub 2020 Dec 23. PMID: 33334893; PMCID: PMC7948397.

62: Willrich MAV, Ladwig PM, Martinez MA, Sridharan MR, Go RS, Murray DL;

Complement Alternative Pathway Thrombotic Microangiopathy (CAP-TMA) Disease

Oriented Group at the Mayo Clinic. Monitoring Ravulizumab effect on complement

assays. J Immunol Methods. 2021 Mar;490:112944. doi: 10.1016/j.jim.2020.112944.

Epub 2020 Dec 13. PMID: 33321132.

63: Devalaraja-Narashimha K, Meagher K, Luo Y, Huang C, Kaplan T, Muthuswamy A,

Halasz G, Casanova S, O'Brien J, Peyser Boiarsky R, McWhirter J, Gartner H, Bai

Y, MacDonnell S, Liu C, Hu Y, Latuszek A, Wei Y, Prasad S, Huang T, Yancopoulos

G, Murphy A, Olson W, Zambrowicz B, Macdonald L, Morton LG. Humanized C3 Mouse:

A Novel Accelerated Model of C3 Glomerulopathy. J Am Soc Nephrol. 2021

Jan;32(1):99-114. doi: 10.1681/ASN.2020050698. Epub 2020 Dec 7. PMID: 33288630;

PMCID: PMC7894673.

64: Li L, Wei T, Liu S, Wang C, Zhao M, Feng Y, Ma L, Lu Y, Fu P, Liu J.

Complement C5 activation promotes type 2 diabetic kidney disease via activating

STAT3 pathway and disrupting the gut-kidney axis. J Cell Mol Med. 2021

Jan;25(2):960-974. doi: 10.1111/jcmm.16157. Epub 2020 Dec 6. PMID: 33280239;

PMCID: PMC7812276.

65: Martinez APG, Abreu PAE, de Arruda Vasconcellos S, Ho PL, Ferreira VP, Saggu

G, Barbosa AS, Isaac L. The Role of Properdin in Killing of Non-Pathogenic

<i>Leptospira biflexa</i>. Front Immunol. 2020 Nov 10;11:572562. doi:

10.3389/fimmu.2020.572562. PMID: 33240263; PMCID: PMC7683387.

66: Koenig A, Mezaache S, Callemeyn J, Barba T, Mathias V, Sicard A, Charreau B,

Rabeyrin M, Dijoud F, Picard C, Meas-Yedid V, Olivo-Marin JC, Morelon E, Naesens

M, Dubois V, Thaunat O. Missing Self-Induced Activation of NK Cells Combines

with Non-Complement-Fixing Donor-Specific Antibodies to Accelerate Kidney

Transplant Loss in Chronic Antibody-Mediated Rejection. J Am Soc Nephrol. 2021

Feb;32(2):479-494. doi: 10.1681/ASN.2020040433. Epub 2020 Nov 25. PMID:

33239394; PMCID: PMC8054908.

67: Malekshahi Z, Schiela B, Bernklau S, Banki Z, Würzner R, Stoiber H.

Interference of the Zika Virus E-Protein With the Membrane Attack Complex of the

Complement System. Front Immunol. 2020 Oct 28;11:569549. doi:

10.3389/fimmu.2020.569549. PMID: 33193347; PMCID: PMC7655927.

68: Zheng JM, Wang SS, Tian X, Che DJ. Sustained activation of C3aR in a human

podocyte line impairs the morphological maturation of the cells. Mol Med Rep.

2020 Dec;22(6):5326-5338. doi: 10.3892/mmr.2020.11626. Epub 2020 Oct 22. PMID:

33174024; PMCID: PMC7646996.

69: Yuan X, Yu J, Gerber G, Chaturvedi S, Cole M, Chen H, Metjian A, Sperati CJ,

Braunstein EM, Brodsky RA. Ex vivo assays to detect complement activation in

complementopathies. Clin Immunol. 2020 Dec;221:108616. doi:

10.1016/j.clim.2020.108616. Epub 2020 Oct 24. PMID: 33148511; PMCID: PMC8609776.

70: de Miranda Santos IKF, de Barros Cardoso CR. Commentary on "Complement C3 vs

C5 inhibition in severe COVID-19: Early clinical findings reveal differential

biological efficacy" by D.C. Mastellos et al. Clin Immunol. 2021 Jan;222:108618.

doi: 10.1016/j.clim.2020.108618. Epub 2020 Oct 27. PMID: 33127564; PMCID:

PMC7588791.

71: de Nooijer AH, Grondman I, Janssen NAF, Netea MG, Willems L, van de Veerdonk

FL, Giamarellos-Bourboulis EJ, Toonen EJM, Joosten LAB; RCI-COVID-19 study

group. Complement Activation in the Disease Course of Coronavirus Disease 2019

and Its Effects on Clinical Outcomes. J Infect Dis. 2021 Feb 3;223(2):214-224.

doi: 10.1093/infdis/jiaa646. PMID: 33038254; PMCID: PMC7797765.

72: Tille A, Lehnert T, Zipfel PF, Figge MT. Quantification of Factor H Mediated

Self vs. Non-self Discrimination by Mathematical Modeling. Front Immunol. 2020

Sep 2;11:1911. doi: 10.3389/fimmu.2020.01911. PMID: 33013842; PMCID: PMC7493836.

73: Mastellos DC, Pires da Silva BGP, Fonseca BAL, Fonseca NP, Auxiliadora-

Martins M, Mastaglio S, Ruggeri A, Sironi M, Radermacher P, Chrysanthopoulou A,

Skendros P, Ritis K, Manfra I, Iacobelli S, Huber-Lang M, Nilsson B, Yancopoulou

D, Connolly ES, Garlanda C, Ciceri F, Risitano AM, Calado RT, Lambris JD.

Complement C3 vs C5 inhibition in severe COVID-19: Early clinical findings

reveal differential biological efficacy. Clin Immunol. 2020 Nov;220:108598. doi:

10.1016/j.clim.2020.108598. Epub 2020 Sep 19. PMID: 32961333; PMCID: PMC7501834.

74: Troldborg A, Halkjær L, Pedersen H, Hansen A, Loft AG, Lindegaard H,

Stengaard-Pedersen K, Graversen JH, Palarasah Y, Thiel S. Complement activation

in human autoimmune diseases and mouse models; employing a sandwich immunoassay

specific for C3dg. J Immunol Methods. 2020 Nov;486:112866. doi:

10.1016/j.jim.2020.112866. Epub 2020 Sep 15. PMID: 32941885.

75: Pedersen H, Jensen RK, Jensen JMB, Fox R, Pedersen DV, Olesen HG, Hansen AG,

Christiansen D, Mazarakis SMM, Lojek N, Hansen P, Gadeberg TAF, Zarantonello A,

Laursen NS, Mollnes TE, Johnson MB, Stevens B, Thiel S, Andersen GR. A

Complement C3-Specific Nanobody for Modulation of the Alternative Cascade

Identifies the C-Terminal Domain of C3b as Functional in C5 Convertase Activity.

J Immunol. 2020 Oct 15;205(8):2287-2300. doi: 10.4049/jimmunol.2000752. Epub

2020 Sep 16. PMID: 32938727.

76: Teixeira GQ, Yong Z, Goncalves RM, Kuhn A, Riegger J, Brisby H, Barreto

Henriksson H, Ruf M, Nerlich A, Mauer UM, Ignatius A, Brenner RE, Neidlinger-

Wilke C. Terminal complement complex formation is associated with intervertebral

disc degeneration. Eur Spine J. 2021 Jan;30(1):217-226. doi:

10.1007/s00586-020-06592-4. Epub 2020 Sep 16. PMID: 32936402.

77: Mühlig AK, Keir LS, Abt JC, Heidelbach HS, Horton R, Welsh GI, Meyer-

Schwesinger C, Licht C, Coward RJ, Fester L, Saleem MA, Oh J. Podocytes Produce

and Secrete Functional Complement C3 and Complement Factor H. Front Immunol.

2020 Aug 14;11:1833. doi: 10.3389/fimmu.2020.01833. PMID: 32922395; PMCID:

PMC7457071.

78: Zhang J, Song L, Pedersen DV, Li A, Lambris JD, Andersen GR, Mollnes TE, Ma

YJ, Garred P. Soluble collectin-12 mediates C3-independent docking of properdin

that activates the alternative pathway of complement. Elife. 2020 Sep

10;9:e60908. doi: 10.7554/eLife.60908. PMID: 32909942; PMCID: PMC7511233.

79: Yu J, Yuan X, Chen H, Chaturvedi S, Braunstein EM, Brodsky RA. Direct

activation of the alternative complement pathway by SARS-CoV-2 spike proteins is

blocked by factor D inhibition. Blood. 2020 Oct 29;136(18):2080-2089. doi:

10.1182/blood.2020008248. PMID: 32877502; PMCID: PMC7596849.

80: Haque A, Cortes C, Alam MN, Sreedhar M, Ferreira VP, Pangburn MK.

Characterization of Binding Properties of Individual Functional Sites of Human

Complement Factor H. Front Immunol. 2020 Aug 4;11:1728. doi:

10.3389/fimmu.2020.01728. PMID: 32849614; PMCID: PMC7417313.

81: Lammerts RGM, Talsma DT, Dam WA, Daha MR, Seelen MAJ, Berger SP, van den

Born J. Properdin Pattern Recognition on Proximal Tubular Cells Is Heparan

Sulfate/Syndecan-1 but Not C3b Dependent and Can Be Blocked by Tick Protein

Salp20. Front Immunol. 2020 Aug 7;11:1643. doi: 10.3389/fimmu.2020.01643. PMID:

32849563; PMCID: PMC7426487.

82: Dekkers G, Brouwer MC, Jeremiasse J, Kamp A, Biggs RM, van Mierlo G, Lauder

S, Katti S, Kuijpers TW, Rispens T, Jongerius I. Unraveling the Effect of a

Potentiating Anti-Factor H Antibody on Atypical Hemolytic Uremic Syndrome-

Associated Factor H Variants. J Immunol. 2020 Oct 1;205(7):1778-1786. doi:

10.4049/jimmunol.2000368. Epub 2020 Aug 26. PMID: 32848031.

83: Chen JY, Galwankar NS, Emch HN, Menon SS, Cortes C, Thurman JM, Merrill SA,

Brodsky RA, Ferreira VP. Properdin Is a Key Player in Lysis of Red Blood Cells

and Complement Activation on Endothelial Cells in Hemolytic Anemias Caused by

Complement Dysregulation. Front Immunol. 2020 Jul 22;11:1460. doi:

10.3389/fimmu.2020.01460. PMID: 32793201; PMCID: PMC7387411.

84: Stepniewska J, Dolegowska B, Golembiewska E, Marchelek-Mysliwiec M, Domanski

M, Ciechanowski K, Zair L. The activation of complement system in different

types of renal replacement therapy. J Physiol Pharmacol. 2020 Apr;71(2). doi:

10.26402/jpp.2020.2.12. Epub 2020 Aug 8. PMID: 32776910.

85: Laurence J, Mulvey JJ, Seshadri M, Racanelli A, Harp J, Schenck EJ, Zappetti

D, Horn EM, Magro CM. Anti-complement C5 therapy with eculizumab in three cases

of critical COVID-19. Clin Immunol. 2020 Oct;219:108555. doi:

10.1016/j.clim.2020.108555. Epub 2020 Aug 6. PMID: 32771488; PMCID: PMC7410014.

86: Zarantonello A, Presumey J, Simoni L, Yalcin E, Fox R, Hansen A, Olesen HG,

Thiel S, Johnson MB, Stevens B, Laursen NS, Carroll MC, Andersen GR. An

Ultrahigh-Affinity Complement C4b-Specific Nanobody Inhibits In Vivo Assembly of

the Classical Pathway Proconvertase. J Immunol. 2020 Sep 15;205(6):1678-1694.

doi: 10.4049/jimmunol.2000528. Epub 2020 Aug 7. PMID: 32769120.

87: Skendros P, Mitsios A, Chrysanthopoulou A, Mastellos DC, Metallidis S,

Rafailidis P, Ntinopoulou M, Sertaridou E, Tsironidou V, Tsigalou C, Tektonidou

M, Konstantinidis T, Papagoras C, Mitroulis I, Germanidis G, Lambris JD, Ritis

K. Complement and tissue factor-enriched neutrophil extracellular traps are key

drivers in COVID-19 immunothrombosis. J Clin Invest. 2020 Nov

2;130(11):6151-6157. doi: 10.1172/JCI141374. PMID: 32759504; PMCID: PMC7598040.

88: Angeletti A, Cantarelli C, Petrosyan A, Andrighetto S, Budge K, D'Agati VD,

Hartzell S, Malvi D, Donadei C, Thurman JM, Galešić-Ljubanović D, He JC, Xiao W,

Campbell KN, Wong J, Fischman C, Manrique J, Zaza G, Fiaccadori E, La Manna G,

Fribourg M, Leventhal J, Da Sacco S, Perin L, Heeger PS, Cravedi P. Loss of

decay-accelerating factor triggers podocyte injury and glomerulosclerosis. J Exp

Med. 2020 Sep 7;217(9):e20191699. doi: 10.1084/jem.20191699. PMID: 32717081;

PMCID: PMC7478737.

89: Jackson WD, Gulino A, Fossati-Jimack L, Castro Seoane R, Tian K, Best K,

Köhl J, Belmonte B, Strid J, Botto M. C3 Drives Inflammatory Skin Carcinogenesis

Independently of C5. J Invest Dermatol. 2021 Feb;141(2):404-414.e6. doi:

10.1016/j.jid.2020.06.025. Epub 2020 Jul 16. PMID: 32682912; PMCID: PMC8150327.

90: Nogueras-Ortiz CJ, Mahairaki V, Delgado-Peraza F, Das D, Avgerinos K, Eren

E, Hentschel M, Goetzl EJ, Mattson MP, Kapogiannis D. Astrocyte- and Neuron-

Derived Extracellular Vesicles from Alzheimer's Disease Patients Effect

Complement-Mediated Neurotoxicity. Cells. 2020 Jul 4;9(7):1618. doi:

10.3390/cells9071618. PMID: 32635578; PMCID: PMC7407141.

91: Tachi A, Moriyama Y, Tsuda H, Miki R, Ushida T, Miura M, Ito Y, Imai K,

Nakano-Kobayashi T, Hayakawa M, Kikkawa F, Kotani T. A proteome signature of

umbilical cord serum associated with congenital diaphragmatic hernia. Nagoya J

Med Sci. 2020 May;82(2):345-354. doi: 10.18999/nagjms.82.2.345. PMID: 32581413;

PMCID: PMC7276398.

92: Wong SSW, Daniel I, Gangneux JP, Jayapal JM, Guegan H, Dellière S, Lalitha

P, Shende R, Madan T, Bayry J, Guijarro JI, Kuppamuthu D, Aimanianda V.

Differential Interactions of Serum and Bronchoalveolar Lavage Fluid Complement

Proteins with Conidia of Airborne Fungal Pathogen Aspergillus fumigatus. Infect

Immun. 2020 Aug 19;88(9):e00212-20. doi: 10.1128/IAI.00212-20. PMID: 32571987;

PMCID: PMC7440762.

93: Tao J, Song D, Liu XL, Yu F, Zhao MH. Circulating anti-C3b IgG in lupus

nephritis: A large cohort study. Clin Immunol. 2020 Aug;217:108514. doi:

10.1016/j.clim.2020.108514. Epub 2020 Jun 18. PMID: 32565324.

94: Wang Y, Nanda V, Direnzo D, Ye J, Xiao S, Kojima Y, Howe KL, Jarr KU, Flores

AM, Tsantilas P, Tsao N, Rao A, Newman AAC, Eberhard AV, Priest JR, Ruusalepp A,

Pasterkamp G, Maegdefessel L, Miller CL, Lind L, Koplev S, Björkegren JLM, Owens

GK, Ingelsson E, Weissman IL, Leeper NJ. Clonally expanding smooth muscle cells

promote atherosclerosis by escaping efferocytosis and activating the complement

cascade. Proc Natl Acad Sci U S A. 2020 Jul 7;117(27):15818-15826. doi:

10.1073/pnas.2006348117. Epub 2020 Jun 15. PMID: 32541024; PMCID: PMC7354942.

95: Parry J, Hwang J, Stahel CF, Henderson C, Nadeau J, Stacey S, Beauchamp K,

Moore EE, Stahel PF. Soluble terminal complement activation fragment sC5b-9: a

new serum biomarker for traumatic brain injury? Eur J Trauma Emerg Surg. 2021

Oct;47(5):1491-1497. doi: 10.1007/s00068-020-01407-z. Epub 2020 May 25. PMID:

32451568.

96: Halkjær L, Troldborg A, Pedersen H, Jensen L, Hansen AG, Hansen TK, Bjerre

M, Østergaard JA, Thiel S. Complement Receptor 2 Based Immunoassay Measuring

Activation of the Complement System at C3-Level in Plasma Samples From Mice and

Humans. Front Immunol. 2020 May 5;11:774. doi: 10.3389/fimmu.2020.00774. PMID:

32431705; PMCID: PMC7214740.

97: Dheir H, Sipahi S, Yaylaci S, Köroğlu M, Erdem AF, Karabay O. Is there

relationship between SARS-CoV-2 and the complement C3 and C4? Turk J Med Sci.

2020 Jun 23;50(4):687-688. doi: 10.3906/sag-2004-336. PMID: 32421281; PMCID:

PMC7379471.

98: Cugno M, Meroni PL, Gualtierotti R, Griffini S, Grovetti E, Torri A,

Panigada M, Aliberti S, Blasi F, Tedesco F, Peyvandi F. Complement activation in

patients with COVID-19: A novel therapeutic target. J Allergy Clin Immunol. 2020

Jul;146(1):215-217. doi: 10.1016/j.jaci.2020.05.006. Epub 2020 May 14. PMID:

32417135; PMCID: PMC7224678.

99: Kim H, Kim T, Kim M, Lee HY, Kim Y, Kang MS, Kim J. Activation of the

alternative complement pathway predicts renal outcome in patients with lupus

nephritis. Lupus. 2020 Jul;29(8):862-871. doi: 10.1177/0961203320925165. Epub

2020 May 14. PMID: 32408850.

100: Lin K, Zhang L, Kong M, Yang M, Chen Y, Poptic E, Hoffner M, Xu J, Tam C,

Lin F. Development of an anti-human complement C6 monoclonal antibody that

inhibits the assembly of membrane attack complexes. Blood Adv. 2020 May

12;4(9):2049-2057. doi: 10.1182/bloodadvances.2020001690. PMID: 32396613; PMCID:

PMC7218433.

101: Latuszek A, Liu Y, Olsen O, Foster R, Cao M, Lovric I, Yuan M, Liu N, Chen

H, Zhang Q, Xiao H, Springer C, Ehrlich G, Kamat V, Rafique A, Hu Y, Krueger P,

Huang T, Poueymirou W, Babb R, Rosconi MP, Retter MW, Chen G, Morton L,

Zambrowicz B, Cao J, Romano C, Olson WC. Inhibition of complement pathway

activation with Pozelimab, a fully human antibody to complement component C5.

PLoS One. 2020 May 8;15(5):e0231892. doi: 10.1371/journal.pone.0231892. PMID:

32384086; PMCID: PMC7209288.

102: Zhang Y, Keenan A, Dai DF, May KS, Anderson EE, Lindorfer MA, Henrich JB,

Pitcher GR, Taylor RP, Smith RJ. C3(H2O) prevents rescue of complement-mediated

C3 glomerulopathy in Cfh-/- Cfd-/- mice. JCI Insight. 2020 May 7;5(9):e135758.

doi: 10.1172/jci.insight.135758. PMID: 32376801; PMCID: PMC7253029.

103: Pedersen H, Jensen RK, Hansen AG, Gadeberg TAF, Thiel S, Laursen NS,

Andersen GR. A C3-specific nanobody that blocks all three activation pathways in

the human and murine complement system. J Biol Chem. 2020 Jun

26;295(26):8746-8758. doi: 10.1074/jbc.RA119.012339. Epub 2020 May 6. PMID:

32376685; PMCID: PMC7324514.

104: Arriens C, Alexander RV, Narain S, Saxena A, Collins CE, Wallace DJ,

Massarotti E, Conklin J, Kalunian KC, Putterman C, Ramsey-Goldman R, Buyon JP,

Askanase A, Furie RA, James JA, Bello GA, Manzi S, Ahearn J, O'Malley T,

Weinstein A, Dervieux T. Cell-bound complement activation products associate

with lupus severity in SLE. Lupus Sci Med. 2020 Apr;7(1):e000377. doi:

10.1136/lupus-2019-000377. PMID: 32371480; PMCID: PMC7228655.

105: Mastaglio S, Ruggeri A, Risitano AM, Angelillo P, Yancopoulou D, Mastellos

DC, Huber-Lang M, Piemontese S, Assanelli A, Garlanda C, Lambris JD, Ciceri F.

The first case of COVID-19 treated with the complement C3 inhibitor AMY-101.

Clin Immunol. 2020 Jun;215:108450. doi: 10.1016/j.clim.2020.108450. Epub 2020

Apr 29. PMID: 32360516; PMCID: PMC7189192.

106: Martínez-López D, Roldan-Montero R, García-Marqués F, Nuñez E, Jorge I,

Camafeita E, Minguez P, Rodriguez de Cordoba S, López-Melgar B, Lara-Pezzi E,

Fernández-Ortiz A, Ibáñez B, Valdivielso JM, Fuster V, Michel JB, Blanco-Colio

LM, Vázquez J, Martin-Ventura JL. Complement C5 Protein as a Marker of

Subclinical Atherosclerosis. J Am Coll Cardiol. 2020 Apr 28;75(16):1926-1941.

doi: 10.1016/j.jacc.2020.02.058. PMID: 32327104.

107: Borras C, Delaunay K, Slaoui Y, Abache T, Jorieux S, Naud MC, Sanharawi ME,

Gelize E, Lassiaz P, An N, Kowalczuk L, Ayassami C, Moulin A, Behar-Cohen F,

Mascarelli F, Dinet V. Mechanisms of FH Protection Against Neovascular AMD.

Front Immunol. 2020 Apr 3;11:443. doi: 10.3389/fimmu.2020.00443. PMID: 32318056;

PMCID: PMC7146894.

108: Shivshankar P, Li YD, Mueller-Ortiz SL, Wetsel RA. In response to

complement anaphylatoxin peptides C3a and C5a, human vascular endothelial cells

migrate and mediate the activation of B-cells and polarization of T-cells. FASEB

J. 2020 Jun;34(6):7540-7560. doi: 10.1096/fj.201902397R. Epub 2020 Apr 17. PMID:

32301538.

109: Itami H, Hara S, Samejima K, Tsushima H, Morimoto K, Okamoto K, Kosugi T,

Kawano T, Fujiki K, Kitada H, Hatakeyama K, Tsuruya K, Ohbayashi C. Complement

activation is associated with crescent formation in IgA nephropathy. Virchows

Arch. 2020 Oct;477(4):565-572. doi: 10.1007/s00428-020-02800-0. Epub 2020 Apr

16. PMID: 32300880.

110: Fromell K, Adler A, Åman A, Manivel VA, Huang S, Dührkop C, Sandholm K,

Ekdahl KN, Nilsson B. Assessment of the Role of C3(H<sub>2</sub>O) in the

Alternative Pathway. Front Immunol. 2020 Mar 31;11:530. doi:

10.3389/fimmu.2020.00530. PMID: 32296436; PMCID: PMC7136553.

111: Sharma S, Bhatnagar R, Gaur D. <i>Bacillus anthracis</i> Poly-γ-D-Glutamate

Capsule Inhibits Opsonic Phagocytosis by Impeding Complement Activation. Front

Immunol. 2020 Mar 31;11:462. doi: 10.3389/fimmu.2020.00462. PMID: 32296419;

PMCID: PMC7138205.

112: Denzinger M, Held M, Daigeler A, Krajewski S, Link A. Complement activation

at the interface of wound dressings and blood does not influence keratinocyte

migration/proliferation in vitro. Wound Repair Regen. 2020 Jul;28(4):573-575.

doi: 10.1111/wrr.12817. Epub 2020 Apr 30. PMID: 32281172.

113: Skopelja-Gardner S, Colonna L, Hermanson P, Sun X, Tanaka L, Tai J, Nguyen

Y, Snyder JM, Alpers CE, Hudkins KL, Salant DJ, Peng Y, Elkon KB. Complement

Deficiencies Result in Surrogate Pathways of Complement Activation in Novel

Polygenic Lupus-like Models of Kidney Injury. J Immunol. 2020 May

15;204(10):2627-2640. doi: 10.4049/jimmunol.1901473. Epub 2020 Apr 1. PMID:

32238460; PMCID: PMC7365257.

114: van den Heuvel LP, van de Kar NCAJ, Duineveld C, Sarlea A, van der Velden

TJAM, Liebrand WTB, van Kraaij S, Schjalm C, Bouwmeester R, Wetzels JFM, Mollnes

TE, Volokhina EB. The complement component C5 is not responsible for the

alternative pathway activity in rabbit erythrocyte hemolytic assays during

eculizumab treatment. Cell Mol Immunol. 2020 Jun;17(6):653-655. doi:

10.1038/s41423-020-0406-y. Epub 2020 Mar 24. PMID: 32210393; PMCID: PMC7264279.

115: Heesterbeek TJ, Lechanteur YTE, Lorés-Motta L, Schick T, Daha MR, Altay L,

Liakopoulos S, Smailhodzic D, den Hollander AI, Hoyng CB, de Jong EK, Klevering

BJ. Complement Activation Levels Are Related to Disease Stage in AMD. Invest

Ophthalmol Vis Sci. 2020 Mar 9;61(3):18. doi: 10.1167/iovs.61.3.18. PMID:

32176267; PMCID: PMC7401663.

116: Grinde D, Øverland T, Lima K, Schjalm C, Mollnes TE, Abrahamsen TG.

Complement Activation in 22q11.2 Deletion Syndrome. J Clin Immunol. 2020

Apr;40(3):515-523. doi: 10.1007/s10875-020-00766-x. Epub 2020 Mar 9. PMID:

32152940; PMCID: PMC7142058.

117: Huang C, Fisher KP, Hammer SS, Busik JV. Extracellular Vesicle-Induced

Classical Complement Activation Leads to Retinal Endothelial Cell Damage via MAC

Deposition. Int J Mol Sci. 2020 Mar 1;21(5):1693. doi: 10.3390/ijms21051693.

PMID: 32121610; PMCID: PMC7084203.

118: Shahulhameed S, Vishwakarma S, Chhablani J, Tyagi M, Pappuru RR, Jakati S,

Chakrabarti S, Kaur I. A Systematic Investigation on Complement Pathway

Activation in Diabetic Retinopathy. Front Immunol. 2020 Feb 11;11:154. doi:

10.3389/fimmu.2020.00154. PMID: 32117292; PMCID: PMC7026189.

119: Lackner I, Weber B, Baur M, Fois G, Gebhard F, Pfeifer R, Cinelli P,

Halvachizadeh S, Lipiski M, Cesarovic N, Schrezenmeier H, Huber-Lang M, Pape HC,

Kalbitz M. Complement Activation and Organ Damage After Trauma-Differential

Immune Response Based on Surgical Treatment Strategy. Front Immunol. 2020 Jan

31;11:64. doi: 10.3389/fimmu.2020.00064. PMID: 32117238; PMCID: PMC7025487.

120: Kristensen MK, Hansen MB, Madsen MB, Hansen CB, Pilely K, Hyldegaard O,

Garred P. Complement Activation Is Associated With Mortality in Patients With

Necrotizing Soft-Tissue Infections-A Prospective Observational Study. Front

Immunol. 2020 Jan 31;11:17. doi: 10.3389/fimmu.2020.00017. PMID: 32082310;

PMCID: PMC7006023.

121: Sohn YK, Son S, Choi Y, Hwang DE, Seo HD, Lee JJ, Kim HS. Effective

inhibition of C3a-mediated pro-inflammatory response by a human C3a-specific

protein binder. Biotechnol Bioeng. 2020 Jun;117(6):1904-1908. doi:

10.1002/bit.27309. Epub 2020 Mar 4. PMID: 32068245.

122: Weinstein A. Cell-Bound Complement Activation Products Are Superior to

Serum Complement C3 and C4 Levels to Detect Complement Activation in Systemic

Lupus Erythematosus: Comment on the Article by Aringer et al. Arthritis

Rheumatol. 2020 May;72(5):860. doi: 10.1002/art.41227. Epub 2020 Mar 18. PMID:

32067409.

123: Bakshi S, Cunningham F, Nichols EM, Biedzka-Sarek M, Neisen J, Petit-Frere

S, Bessant C, Bansal L, Peletier LA, Zamuner S, van der Graaf PH. Mathematical

Modelling of Alternative Pathway of Complement System. Bull Math Biol. 2020 Feb

15;82(2):33. doi: 10.1007/s11538-020-00708-z. Erratum in: Bull Math Biol. 2021

Dec 18;84(1):18. PMID: 32062771; PMCID: PMC7024062.

124: Carrara C, Podestà MA, Abbate M, Rizzo P, Piras R, Alberti M, Daina E,

Ruggenenti P, Remuzzi G; on behalf of the EAGLE Study Group. Morphofunctional

Effects of C5 Convertase Blockade in Immune Complex-Mediated

Membranoproliferative Glomerulonephritis: Report of Two Cases with Evidence of

Terminal Complement Activation. Nephron. 2020;144(4):195-203. doi:

10.1159/000505403. Epub 2020 Feb 12. PMID: 32050203.

125: Chauvet S, Berthaud R, Devriese M, Mignotet M, Vieira Martins P, Robe-

Rybkine T, Miteva MA, Gyulkhandanyan A, Ryckewaert A, Louillet F, Merieau E,

Mestrallet G, Rousset-Rouvière C, Thervet E, Hogan J, Ulinski T, Villoutreix BO,

Roumenina L, Boyer O, Frémeaux-Bacchi V. Anti-Factor B Antibodies and Acute

Postinfectious GN in Children. J Am Soc Nephrol. 2020 Apr;31(4):829-840. doi:

10.1681/ASN.2019080851. Epub 2020 Feb 7. PMID: 32034108; PMCID: PMC7191928.

126: Sikorski PM, Commodaro AG, Grigg ME. <i>Toxoplasma gondii</i> Recruits

Factor H and C4b-Binding Protein to Mediate Resistance to Serum Killing and

Promote Parasite Persistence <i>in vivo</i>. Front Immunol. 2020 Jan 17;10:3105.

doi: 10.3389/fimmu.2019.03105. PMID: 32010145; PMCID: PMC6979546.

127: Gulleroglu K, Baskin E, Ozdemir H, Moray G, Haberal M. Recurrence and

Outcomes of Complement-Related Renal Disease After Pediatric Renal

Transplantation. Exp Clin Transplant. 2020 Jan;18(Suppl 1):82-83. doi:

10.6002/ect.TOND-TDTD2019.P28. PMID: 32008503.

128: Nag J, Mukesh RK, Suma SM, Kunnakkadan U, Kumar NA, Johnson JB. A Factor

I-Like Activity Associated with Chikungunya Virus Contributes to Its Resistance

to the Human Complement System. J Virol. 2020 Mar 17;94(7):e02062-19. doi:

10.1128/JVI.02062-19. PMID: 31941783; PMCID: PMC7081913.

129: Dumont C, Mérouani A, Ducruet T, Benoit G, Clermont MJ, Lapeyraque AL, Phan

V, Patey N. Clinical relevance of membrane attack complex deposition in children

with IgA nephropathy and Henoch-Schönlein purpura. Pediatr Nephrol. 2020

May;35(5):843-850. doi: 10.1007/s00467-019-04445-x. Epub 2020 Jan 13. PMID:

31932958.

130: Abe T, Kubo K, Izumoto S, Shimazu S, Goan A, Tanaka T, Koroki T, Saito K,

Kawana R, Ochiai H. Complement Activation in Human Sepsis is Related to Sepsis-

Induced Disseminated Intravascular Coagulation. Shock. 2020 Aug;54(2):198-204.

doi: 10.1097/SHK.0000000000001504. PMID: 31917735.

131: Carlsson H, Sandholm K, Haddish HW, Brudin L, Ekdahl KN, Tjernberg I.

Complement activation in individuals with previous subclinical Lyme borreliosis

and patients with previous Lyme neuroborreliosis. Eur J Clin Microbiol Infect

Dis. 2020 May;39(5):855-862. doi: 10.1007/s10096-019-03807-5. Epub 2019 Dec 31.

PMID: 31893341; PMCID: PMC7182544.

132: Reichhardt MP, Johnson S, Tang T, Morgan T, Tebeka N, Popitsch N, Deme JC,

Jore MM, Lea SM. An inhibitor of complement C5 provides structural insights into

activation. Proc Natl Acad Sci U S A. 2020 Jan 7;117(1):362-370. doi:

10.1073/pnas.1909973116. Epub 2019 Dec 23. PMID: 31871188; PMCID: PMC6955305.

133: Simmons KT, Mazzilli JL, Mueller-Ortiz SL, Domozhirov AY, Garcia CA,

Zsigmond EM, Wetsel RA. Complement Receptor 1 (CR1/CD35)-expressing retinal

pigment epithelial cells as a potential therapy for age-related macular

degeneration. Mol Immunol. 2020 Feb;118:91-98. doi:

10.1016/j.molimm.2019.11.007. Epub 2019 Dec 26. PMID: 31862673.

134: Gupta P, Tripathy AS. Alternative pathway of complement activation has a

beneficial role against Chandipura virus infection. Med Microbiol Immunol. 2020

Apr;209(2):109-124. doi: 10.1007/s00430-019-00648-z. Epub 2019 Nov 28. PMID:

31781935; PMCID: PMC7223837.

135: Sartain S, Shubert S, Wu MF, Wang T, Martinez C. The alternative complement

pathway activation product Ba as a marker for transplant-associated thrombotic

microangiopathy. Pediatr Blood Cancer. 2020 Mar;67(3):e28070. doi:

10.1002/pbc.28070. Epub 2019 Nov 27. PMID: 31774252.

136: Abdel-Latif M. Identification of the complement 9-binding protein in

Setaria equina excretory-secretory products. Parasite Immunol. 2020

Feb;42(2):e12686. doi: 10.1111/pim.12686. Epub 2019 Dec 2. PMID: 31745990.

137: Lammerts RGM, Eisenga MF, Alyami M, Daha MR, Seelen MA, Pol RA, van den

Born J, Sanders JS, Bakker SJL, Berger SP. Urinary Properdin and sC5b-9 Are

Independently Associated With Increased Risk for Graft Failure in Renal

Transplant Recipients. Front Immunol. 2019 Oct 24;10:2511. doi:

10.3389/fimmu.2019.02511. PMID: 31736953; PMCID: PMC6830301.

138: Makou E, Bailey RG, Johnston H, Parkin JD, Hulme AN, Hähner G, Barlow PN.

Combining SPR with atomic-force microscopy enables single-molecule insights into

activation and suppression of the complement cascade. J Biol Chem. 2019 Dec

27;294(52):20148-20163. doi: 10.1074/jbc.RA119.010913. Epub 2019 Nov 12. PMID:

31719147; PMCID: PMC6937562.

139: Wang Y, Su Y, Lai W, Huang X, Chu K, Brown J, Hong G. Salidroside Restores

an Anti-inflammatory Endothelial Phenotype by Selectively Inhibiting Endothelial

Complement After Oxidative Stress. Inflammation. 2020 Feb;43(1):310-325. doi:

10.1007/s10753-019-01121-y. PMID: 31701353.

140: Palomo M, Blasco M, Molina P, Lozano M, Praga M, Torramade-Moix S,

Martinez-Sanchez J, Cid J, Escolar G, Carreras E, Paules C, Crispi F, Quintana

LF, Poch E, Rodas L, Goma E, Morelle J, Espinosa M, Morales E, Avila A, Cabello

V, Ariceta G, Chocron S, Manrique J, Barros X, Martin N, Huerta A, Fraga-

Rodriguez GM, Cao M, Martin M, Romera AM, Moreso F, Manonelles A, Gratacos E,

Pereira A, Campistol JM, Diaz-Ricart M. Complement Activation and Thrombotic

Microangiopathies. Clin J Am Soc Nephrol. 2019 Dec 6;14(12):1719-1732. doi:

10.2215/CJN.05830519. Epub 2019 Nov 6. PMID: 31694864; PMCID: PMC6895490.

141: Tjernberg AR, Woksepp H, Sandholm K, Johansson M, Dahle C, Ludvigsson JF,

Bonnedahl J, Nilsson P, Ekdahl KN. Celiac disease and complement activation in

response to Streptococcus pneumoniae. Eur J Pediatr. 2020 Jan;179(1):133-140.

doi: 10.1007/s00431-019-03490-w. Epub 2019 Nov 5. PMID: 31691001; PMCID:

PMC6942560.

142: Chisholm CF, Behnke W, Pokhilchuk Y, Frazer-Abel AA, Randolph TW.

Subvisible Particles in IVIg Formulations Activate Complement in Human Serum. J

Pharm Sci. 2020 Jan;109(1):558-565. doi: 10.1016/j.xphs.2019.10.041. Epub 2019

Oct 28. PMID: 31672401; PMCID: PMC6948146.

143: Zilberman-Itskovich S, Abu-Hamad R, Stark M, Efrati S. Effect of anti-C5

antibody on recuperation from ischemia/reperfusion-induced acute kidney injury.

Ren Fail. 2019 Nov;41(1):967-975. doi: 10.1080/0886022X.2019.1677248. PMID:

31662004; PMCID: PMC6830203.

144: Omori T, Oguchi Y, Machida T, Kato Y, Ishida Y, Ojima A, Itagaki K,

Shintake H, Tomita R, Kasai A, Sugano Y, Ogasawara M, Sekine H, Sekiryu T.

Evidence for Activation of Lectin and Classical Pathway Complement Components in

Aqueous Humor of Neovascular Age-Related Macular Degeneration. Ophthalmic Res.

2020;63(3):252-258. doi: 10.1159/000503258. Epub 2019 Oct 23. PMID: 31645047.

145: Hester CG, Frank MM. Complement activation by IgG containing immune

complexes regulates the interaction of C1q with its ligands. Mol Immunol. 2019

Dec;116:117-130. doi: 10.1016/j.molimm.2019.10.004. Epub 2019 Oct 18. PMID:

31634815.

146: Tabacco S, Giannini A, Garufi C, Botta A, Salvi S, Del Sordo G, Benedetti

Panici P, Lanzone A, De Carolis S. Complementemia in pregnancies with

antiphospholipid syndrome. Lupus. 2019 Nov;28(13):1503-1509. doi:

10.1177/0961203319882507. Epub 2019 Oct 17. PMID: 31623520.

147: Aykut B, Pushalkar S, Chen R, Li Q, Abengozar R, Kim JI, Shadaloey SA, Wu

D, Preiss P, Verma N, Guo Y, Saxena A, Vardhan M, Diskin B, Wang W, Leinwand J,

Kurz E, Kochen Rossi JA, Hundeyin M, Zambrinis C, Li X, Saxena D, Miller G. The

fungal mycobiome promotes pancreatic oncogenesis via activation of MBL. Nature.

2019 Oct;574(7777):264-267. doi: 10.1038/s41586-019-1608-2. Epub 2019 Oct 2.

PMID: 31578522; PMCID: PMC6858566.

148: Battin C, De Sousa Linhares A, Paster W, Isenman DE, Wahrmann M, Leitner J,

Zlabinger GJ, Steinberger P, Hofer J. Neuropilin-1 Acts as a Receptor for

Complement Split Products. Front Immunol. 2019 Sep 13;10:2209. doi:

10.3389/fimmu.2019.02209. PMID: 31572401; PMCID: PMC6753332.

149: van den Bos RM, Pearce NM, Granneman J, Brondijk THC, Gros P. Insights Into

Enhanced Complement Activation by Structures of Properdin and Its Complex With

the C-Terminal Domain of C3b. Front Immunol. 2019 Sep 4;10:2097. doi:

10.3389/fimmu.2019.02097. PMID: 31552043; PMCID: PMC6736995.

150: Frid MG, McKeon BA, Thurman JM, Maron BA, Li M, Zhang H, Kumar S, Sullivan

T, Laskowsky J, Fini MA, Hu S, Tuder RM, Gandjeva A, Wilkins MR, Rhodes CJ,

Ghataorhe P, Leopold JA, Wang RS, Holers VM, Stenmark KR. Immunoglobulin-driven

Complement Activation Regulates Proinflammatory Remodeling in Pulmonary

Hypertension. Am J Respir Crit Care Med. 2020 Jan 15;201(2):224-239. doi:

10.1164/rccm.201903-0591OC. PMID: 31545648; PMCID: PMC6961733.

151: Caravaca-Fontan F, Praga M. Complement inhibitors are useful in secondary

hemolytic uremic syndromes. Kidney Int. 2019 Oct;96(4):826-829. doi:

10.1016/j.kint.2019.07.006. PMID: 31543153.

152: Akk A, Springer LE, Yang L, Hamilton-Burdess S, Lambris JD, Yan H, Hu Y, Wu

X, Hourcade DE, Miller MJ, Pham CTN. Complement activation on neutrophils

initiates endothelial adhesion and extravasation. Mol Immunol. 2019

Oct;114:629-642. doi: 10.1016/j.molimm.2019.09.011. Epub 2019 Sep 19. PMID:

31542608; PMCID: PMC6815348.

153: DeCordova S, Abdelgany A, Murugaiah V, Pathan AA, Nayak A, Walker T,

Shastri A, Alrokayan SH, Khan HA, Singh SK, De Pennington N, Sim RB, Kishore U.

Secretion of functionally active complement factor H related protein 5 (FHR5) by

primary tumour cells derived from Glioblastoma Multiforme patients.

Immunobiology. 2019 Sep;224(5):625-631. doi: 10.1016/j.imbio.2019.07.006. Epub

2019 Aug 5. PMID: 31519376.

154: Faria B, Gaya da Costa M, Poppelaars F, Franssen CFM, Pestana M, Berger SP,

Daha MR, Gaillard CAJM, Seelen MA. Administration of Intravenous Iron

Formulations Induces Complement Activation <i>in-vivo</i>. Front Immunol. 2019

Aug 21;10:1885. doi: 10.3389/fimmu.2019.01885. PMID: 31497011; PMCID:

PMC6712170.

155: Scambi C, Ugolini S, Tonello M, Bortolami O, De Franceschi L, Castagna A,

Lotti V, Corbella M, Raffaelli R, Caramaschi P, Mattia E, Biasi D, Ruffatti A.

Complement activation in the plasma and placentas of women with different

subsets of antiphospholipid syndrome. Am J Reprod Immunol. 2019

Dec;82(6):e13185. doi: 10.1111/aji.13185. Epub 2019 Sep 16. PMID: 31479579.

156: Balestracci A, Meni Bataglia L, Toledo I, Beaudoin L, Alvarado C. C3 levels

and acute outcomes in Shiga toxin-related hemolytic uremic syndrome. Pediatr

Nephrol. 2020 Feb;35(2):331-339. doi: 10.1007/s00467-019-04334-3. Epub 2019 Sep

2. Erratum in: Pediatr Nephrol. 2019 Oct 30;: PMID: 31475299.

157: Jensen TS, Opstrup KV, Christiansen G, Rasmussen PV, Thomsen ME, Justesen

DL, Schønheyder HC, Lausen M, Birkelund S. Complement mediated Klebsiella

pneumoniae capsule changes. Microbes Infect. 2020 Jan-Feb;22(1):19-30. doi:

10.1016/j.micinf.2019.08.003. Epub 2019 Aug 29. PMID: 31473336.

158: Pérez-Alós L, Bayarri-Olmos R, Skjoedt MO, Garred P. Combining MAP-1:CD35

or MAP-1:CD55 fusion proteins with pattern-recognition molecules as novel

targeted modulators of the complement cascade. FASEB J. 2019

Nov;33(11):12723-12734. doi: 10.1096/fj.201901643R. Epub 2019 Aug 30. PMID:

31469600; PMCID: PMC6902692.

159: Ramsey-Goldman R, Alexander RV, Massarotti EM, Wallace DJ, Narain S,

Arriens C, Collins CE, Saxena A, Putterman C, Kalunian KC, O'Malley T, Dervieux

T, Weinstein A. Complement Activation in Patients With Probable Systemic Lupus

Erythematosus and Ability to Predict Progression to American College of

Rheumatology-Classified Systemic Lupus Erythematosus. Arthritis Rheumatol. 2020

Jan;72(1):78-88. doi: 10.1002/art.41093. Epub 2019 Nov 25. PMID: 31469249;

PMCID: PMC6972605.

160: Schein TN, Blackburn TE, Heath SL, Barnum SR. Plasma levels of soluble

membrane attack complex are elevated despite viral suppression in HIV patients

with poor immune reconstitution. Clin Exp Immunol. 2019 Dec;198(3):359-366. doi:

10.1111/cei.13366. Epub 2019 Sep 8. PMID: 31461782; PMCID: PMC6857077.

161: Silberreis K, Niesler N, Rades N, Haag R, Dernedde J. Sulfated Dendritic

Polyglycerol Is a Potent Complement Inhibitor. Biomacromolecules. 2019 Oct

14;20(10):3809-3818. doi: 10.1021/acs.biomac.9b00889. Epub 2019 Sep 9. PMID:

31461260.

162: Balola AHA, Mayer B, Bartolmäs T, Salama A. Sublytic Terminal Complement

Components Induce Eryptosis in Autoimmune Haemolytic Anaemia Related to IgM

Autoantibodies. Cell Physiol Biochem. 2019;53(3):453-464. doi:

10.33594/000000150. PMID: 31448885.

163: Cruz JW, Damko E, Modi B, Tu N, Meagher K, Voronina V, Gartner H, Ehrlich

G, Rafique A, Babb R, Aneja P, Potocky TB, D' Orvilliers A, Coppi A, E SY, Qiu

H, Williams CM, Bennett BL, Chen G, Macdonald L, Olson W, Lin JC, Stahl N,

Murphy AJ, Kyratsous CA, Prasad BC. A novel bispecific antibody platform to

direct complement activity for efficient lysis of target cells. Sci Rep. 2019

Aug 19;9(1):12031. doi: 10.1038/s41598-019-48461-1. PMID: 31427700; PMCID:

PMC6700171.

164: Thomas AM, Gerogianni A, McAdam MB, Fløisand Y, Lau C, Espevik T, Nilsson

PH, Mollnes TE, Barratt-Due A. Complement Component C5 and TLR Molecule CD14

Mediate Heme-Induced Thromboinflammation in Human Blood. J Immunol. 2019 Sep

15;203(6):1571-1578. doi: 10.4049/jimmunol.1900047. Epub 2019 Aug 14. PMID:

31413105; PMCID: PMC6731449.

165: Zhang MF, Huang J, Zhang YM, Qu Z, Wang X, Wang F, Meng LQ, Cheng XY, Cui

Z, Liu G, Zhao MH. Complement activation products in the circulation and urine

of primary membranous nephropathy. BMC Nephrol. 2019 Aug 9;20(1):313. doi:

10.1186/s12882-019-1509-5. PMID: 31399080; PMCID: PMC6688252.

166: Miyake K, Okumi M, Kakuta Y, Unagami K, Furusawa M, Ishida H, Tanabe K.

Prognostic value of C3d-fixing, preformed donor-specific antibodies in

crossmatch-positive living kidney transplantation. Transpl Immunol. 2019

Dec;57:101230. doi: 10.1016/j.trim.2019.101230. Epub 2019 Aug 6. PMID: 31398461.

167: Vercauteren KOA, Lambrecht S, Delanghe J. Preanalytical classical and

alternative complement pathway activity loss. Biochem Med (Zagreb). 2019 Oct

15;29(3):030701. doi: 10.11613/BM.2019.030701. Epub 2019 Aug 5. PMID: 31379459;

PMCID: PMC6610672.

168: Seguin-Devaux C, Plesseria JM, Verschueren C, Masquelier C, Iserentant G,

Fullana M, Józsi M, Cohen JHM, Dervillez X. FHR4-based immunoconjugates direct

complement-dependent cytotoxicity and phagocytosis towards HER2-positive cancer

cells. Mol Oncol. 2019 Dec;13(12):2531-2553. doi: 10.1002/1878-0261.12554. Epub

2019 Sep 30. PMID: 31365168; PMCID: PMC6887587.

169: Cassol CA, Brodsky SV, Satoskar AA, Blissett AR, Cataland S, Nadasdy T.

Eculizumab deposits in vessel walls in thrombotic microangiopathy. Kidney Int.

2019 Sep;96(3):761-768. doi: 10.1016/j.kint.2019.05.008. Epub 2019 May 24. PMID:

31345584.

170: Hokstad I, Deyab G, Wang Fagerland M, Lyberg T, Hjeltnes G, Førre Ø,

Agewall S, Mollnes TE, Hollan I. Tumor necrosis factor inhibitors are associated

with reduced complement activation in spondylarthropathies: An observational

study. PLoS One. 2019 Jul 23;14(7):e0220079. doi: 10.1371/journal.pone.0220079.

PMID: 31335881; PMCID: PMC6650069.

171: Kunnakkadan U, Nag J, Kumar NA, Mukesh RK, Suma SM, Johnson JB. Complement-

Mediated Neutralization of a Potent Neurotropic Human Pathogen, Chandipura

Virus, Is Dependent on C1q. J Virol. 2019 Sep 12;93(19):e00994-19. doi:

10.1128/JVI.00994-19. PMID: 31315998; PMCID: PMC6744236.

172: Mandel-Brehm C, Retallack H, Knudsen GM, Yamana A, Hajj-Ali RA, Calabrese

LH, Tihan T, Sample HA, Zorn KC, Gorman MP, Madan Cohen J, Sreih AG, Marcus JF,

Josephson SA, Douglas VC, Gelfand JM, Wilson MR, DeRisi JL. Exploratory

proteomic analysis implicates the alternative complement cascade in primary CNS

vasculitis. Neurology. 2019 Jul 30;93(5):e433-e444. doi:

10.1212/WNL.0000000000007850. Epub 2019 Jul 3. PMID: 31270218; PMCID:

PMC6693432.

173: Gavriilaki E, Chrysanthopoulou A, Sakellari I, Batsis I, Mallouri D,

Touloumenidou T, Papalexandri A, Mitsios A, Arampatzioglou A, Ritis K, Brodsky

RA, Mitroulis I, Anagnostopoulos A. Linking Complement Activation, Coagulation,

and Neutrophils in Transplant-Associated Thrombotic Microangiopathy. Thromb

Haemost. 2019 Sep;119(9):1433-1440. doi: 10.1055/s-0039-1692721. Epub 2019 Jul

2. PMID: 31266080.

174: Michels MAHM, van de Kar NCAJ, van den Bos RM, van der Velden TJAM, van

Kraaij SAW, Sarlea SA, Gracchi V, Oosterveld MJS, Volokhina EB, van den Heuvel

LPWJ. Novel Assays to Distinguish Between Properdin-Dependent and Properdin-

Independent C3 Nephritic Factors Provide Insight Into Properdin-Inhibiting

Therapy. Front Immunol. 2019 Jun 17;10:1350. doi: 10.3389/fimmu.2019.01350.

PMID: 31263464; PMCID: PMC6590259.

175: Kawakita C, Kinomura M, Otaka N, Kitagawa M, Sugiyama H, Kusano N, Mizuno

M, Wada J. HIV-associated Immune Complex Kidney Disease with C3-dominant

Deposition Induced by HIV Infection after Treatment of IgA Nephropathy. Intern

Med. 2019 Oct 15;58(20):3001-3007. doi: 10.2169/internalmedicine.2439-18. Epub

2019 Jun 27. PMID: 31243235; PMCID: PMC6859386.

176: Freire PC, Muñoz CH, Derhaschnig U, Schoergenhofer C, Firbas C, Parry GC,

Panicker S, Gilbert JC, Stingl G, Jilma B, Heil PM. Specific Inhibition of the

Classical Complement Pathway Prevents C3 Deposition along the Dermal-Epidermal

Junction in Bullous Pemphigoid. J Invest Dermatol. 2019

Dec;139(12):2417-2424.e2. doi: 10.1016/j.jid.2019.04.025. Epub 2019 Jun 20.

PMID: 31229501.

177: Snell JR, Monticello CR, Her C, Ross EL, Frazer-Abel AA, Carpenter JF,

Randolph TW. DEHP Nanodroplets Leached From Polyvinyl Chloride IV Bags Promote

Aggregation of IVIG and Activate Complement in Human Serum. J Pharm Sci. 2020

Jan;109(1):429-442. doi: 10.1016/j.xphs.2019.06.015. Epub 2019 Jun 21. PMID:

31229435; PMCID: PMC6951798.

178: García L, Pena CE, Maldonado RÁ, Costi C, Mamberti M, Martins E, García MA.

Increased renal damage in hypocomplementemic patients with ANCA-associated

vasculitis: retrospective cohort study. Clin Rheumatol. 2019

Oct;38(10):2819-2824. doi: 10.1007/s10067-019-04636-9. Epub 2019 Jun 20. PMID:

31222573.

179: Ohlsson S, Holm L, Hansson C, Ohlsson SM, Gunnarsson L, Pettersson Å,

Skattum L. Neutrophils from ANCA-associated vasculitis patients show an

increased capacity to activate the complement system via the alternative pathway

after ANCA stimulation. PLoS One. 2019 Jun 19;14(6):e0218272. doi:

10.1371/journal.pone.0218272. PMID: 31216309; PMCID: PMC6583988.

180: Silverman SM, Ma W, Wang X, Zhao L, Wong WT. C3- and CR3-dependent

microglial clearance protects photoreceptors in retinitis pigmentosa. J Exp Med.

2019 Aug 5;216(8):1925-1943. doi: 10.1084/jem.20190009. Epub 2019 Jun 17. PMID:

31209071; PMCID: PMC6683998.

181: Lynch AM, Mandava N, Patnaik JL, Frazer-Abel AA, Wagner BD, Palestine AG,

Mathias MT, Siringo FS, Cathcart JN, Holers VM. Systemic activation of the

complement system in patients with advanced age-related macular degeneration.

Eur J Ophthalmol. 2020 Sep;30(5):1061-1068. doi: 10.1177/1120672119857896. Epub

2019 Jun 17. PMID: 31203676.

182: Baas I, Delvasto-Nuñez L, Ligthart P, Brouwer C, Folman C, Reis ES, Ricklin

D, Lambris JD, Wouters D, de Haas M, Jongerius I, Zeerleder SS. Complement C3

inhibition by compstatin Cp40 prevents intra- and extravascular hemolysis of red

blood cells. Haematologica. 2020 Jan 31;105(2):e57-e60. doi:

10.3324/haematol.2019.216028. PMID: 31171642; PMCID: PMC7012486.

183: Roumenina LT, Daugan MV, Noé R, Petitprez F, Vano YA, Sanchez-Salas R,

Becht E, Meilleroux J, Clec'h BL, Giraldo NA, Merle NS, Sun CM, Verkarre V,

Validire P, Selves J, Lacroix L, Delfour O, Vandenberghe I, Thuilliez C, Keddani

S, Sakhi IB, Barret E, Ferré P, Corvaïa N, Passioukov A, Chetaille E, Botto M,

de Reynies A, Oudard SM, Mejean A, Cathelineau X, Sautès-Fridman C, Fridman WH.

Tumor Cells Hijack Macrophage-Produced Complement C1q to Promote Tumor Growth.

Cancer Immunol Res. 2019 Jul;7(7):1091-1105. doi: 10.1158/2326-6066.CIR-18-0891.

Epub 2019 Jun 4. PMID: 31164356.

184: Hulsart-Billström G, Janson O, Engqvist H, Welch K, Hong J.

Thromboinflammation as bioactivity assessment of

H<sub>2</sub>O<sub>2</sub>-alkali modified titanium surfaces. J Mater Sci Mater

Med. 2019 May 24;30(6):66. doi: 10.1007/s10856-019-6248-4. PMID: 31127371;

PMCID: PMC6534515.

185: Gutjahr A, Heck F, Emtenani S, Hammers AK, Hundt JE, Muck P, Siegel DL,

Schmidt E, Stanley JR, Zillikens D, Hammers CM. Bullous pemphigoid autoantibody-

mediated complement fixation is abolished by the low-molecular-weight heparin

tinzaparin sodium. Br J Dermatol. 2019 Sep;181(3):593-594. doi:

10.1111/bjd.18156. Epub 2019 Jun 5. PMID: 31124130.

186: Greenshields AL, Liwski RS. The ABCs (DRDQDPs) of the prozone effect in

single antigen bead HLA antibody testing: Lessons from our highly sensitized

patients. Hum Immunol. 2019 Jul;80(7):478-486. doi:

10.1016/j.humimm.2019.04.019. Epub 2019 May 10. PMID: 31080010.

187: Ali YM, Sim RB, Schwaeble W, Shaaban MI. Enterococcus faecalis Escapes

Complement-Mediated Killing via Recruitment of Complement Factor H. J Infect

Dis. 2019 Aug 9;220(6):1061-1070. doi: 10.1093/infdis/jiz226. PMID: 31058287.

188: Zhu W, Wang Z, Hu S, Gong Y, Liu Y, Song H, Ding X, Fu Y, Yan Y. Human

C5-specific single-chain variable fragment ameliorates brain injury in a model

of NMOSD. Neurol Neuroimmunol Neuroinflamm. 2019 Apr 4;6(3):e561. doi:

10.1212/NXI.0000000000000561. PMID: 31044149; PMCID: PMC6467685.

189: Greenshields A, Bray RA, Gebel HM, Liwski RS. Getting on target:

Development of the novel, prozone-resistant, dual antibody rapid test (DART) for

the LABScreen single antigen bead (SAB) assay. Hum Immunol. 2019

Jul;80(7):468-477. doi: 10.1016/j.humimm.2019.04.017. Epub 2019 Apr 28. PMID:

31040048.

190: Lonati PA, Scavone M, Gerosa M, Borghi MO, Pregnolato F, Curreli D, Podda

G, Femia EA, Barcellini W, Cattaneo M, Tedesco F, Meroni PL. Blood Cell-Bound

C4d as a Marker of Complement Activation in Patients With the Antiphospholipid

Syndrome. Front Immunol. 2019 Apr 12;10:773. doi: 10.3389/fimmu.2019.00773.

PMID: 31031764; PMCID: PMC6474283.

191: Elvington M, Liszewski MK, Liszewski AR, Kulkarni HS, Hachem RR,

Mohanakumar T, Kim AHJ, Atkinson JP. Development and Optimization of an ELISA to

Quantitate C3(H <sub>2</sub> O) as a Marker of Human Disease. Front Immunol.

2019 Apr 4;10:703. doi: 10.3389/fimmu.2019.00703. PMID: 31019515; PMCID:

PMC6458276.

192: Satyam A, Andreo K, Lapchak PH, Dalle Lucca JJ, Davis RB, Tsokos MG,

Shapiro NI, Tsokos GC. Complement Deposition on the Surface of RBC After Trauma

Serves a Biomarker of Moderate Trauma Severity: A Prospective Study. Shock. 2020

Jan;53(1):16-23. doi: 10.1097/SHK.0000000000001348. PMID: 30998651; PMCID:

PMC6790152.

193: Wang Y, Miao Y, Gong K, Cheng X, Chen Y, Zhao MH. Plasma Complement Protein

C3a Level Was Associated with Abdominal Aortic Calcification in Patients on

Hemodialysis. J Cardiovasc Transl Res. 2019 Oct;12(5):496-505. doi:

10.1007/s12265-019-09885-2. Epub 2019 Apr 15. PMID: 30989586.

194: Chua JS, Zandbergen M, Wolterbeek R, Baelde HJ, van Es LA, de Fijter JW,

Bruijn JA, Bajema IM. Complement-mediated microangiopathy in IgA nephropathy and

IgA vasculitis with nephritis. Mod Pathol. 2019 Jul;32(8):1147-1157. doi:

10.1038/s41379-019-0259-z. Epub 2019 Apr 1. PMID: 30936425.

195: Høiland II, Liang RA, Braekkan SK, Pettersen K, Ludviksen JK, Latysheva N,

Snir O, Ueland T, Hindberg K, Mollnes TE, Hansen JB. Complement activation

assessed by the plasma terminal complement complex and future risk of venous

thromboembolism. J Thromb Haemost. 2019 Jun;17(6):934-943. doi:

10.1111/jth.14438. Epub 2019 May 13. PMID: 30920726.

196: Ueda Y, Miwa T, Ito D, Kim H, Sato S, Gullipalli D, Zhou L, Golla M, Song

D, Dunaief JL, Palmer MB, Song WC. Differential contribution of C5aR and C5b-9

pathways to renal thrombic microangiopathy and macrovascular thrombosis in mice

carrying an atypical hemolytic syndrome-related factor H mutation. Kidney Int.

2019 Jul;96(1):67-79. doi: 10.1016/j.kint.2019.01.009. Epub 2019 Feb 27. PMID:

30910380.

197: Goutaudier V, Perrochia H, Mucha S, Bonnet M, Delmas S, Garo F, Garrigue V,

Lepreux S, Pernin V, Serre JE, Szwarc I, Merville P, Ramounau-Pigot A, René C,

Visentin J, Morgan BP, Frémeaux-Bacchi V, Mourad G, Couzi L, Le Quintrec M. C5b9

Deposition in Glomerular Capillaries Is Associated With Poor Kidney Allograft

Survival in Antibody-Mediated Rejection. Front Immunol. 2019 Mar 8;10:235. doi:

10.3389/fimmu.2019.00235. PMID: 30906289; PMCID: PMC6418012.

198: Mansur S, Othman MHD, Ismail AF, Kadir SHSA, Goh PS, Hasbullah H, Ng BC,

Abdullah MS, Kamal F, Abidin MNZ, Lusiana RA. Synthesis and characterisation of

composite sulphonated polyurethane/polyethersulphone membrane for blood

purification application. Mater Sci Eng C Mater Biol Appl. 2019 Jun;99:491-504.

doi: 10.1016/j.msec.2019.01.092. Epub 2019 Jan 23. PMID: 30889724.

199: Galbusera M, Noris M, Gastoldi S, Bresin E, Mele C, Breno M, Cuccarolo P,

Alberti M, Valoti E, Piras R, Donadelli R, Vivarelli M, Murer L, Pecoraro C,

Ferrari E, Perna A, Benigni A, Portalupi V, Remuzzi G. An Ex Vivo Test of

Complement Activation on Endothelium for Individualized Eculizumab Therapy in

Hemolytic Uremic Syndrome. Am J Kidney Dis. 2019 Jul;74(1):56-72. doi:

10.1053/j.ajkd.2018.11.012. Epub 2019 Mar 7. PMID: 30851964.

200: Merle NS, Paule R, Leon J, Daugan M, Robe-Rybkine T, Poillerat V, Torset C,

Frémeaux-Bacchi V, Dimitrov JD, Roumenina LT. P-selectin drives complement

attack on endothelium during intravascular hemolysis in TLR-4/heme-dependent

manner. Proc Natl Acad Sci U S A. 2019 Mar 26;116(13):6280-6285. doi:

10.1073/pnas.1814797116. Epub 2019 Mar 8. PMID: 30850533; PMCID: PMC6442544.

201: Jia M, Zhu L, Zhai YL, Chen P, Xu BY, Guo WY, Shi SF, Liu LJ, Lv JC, Zhang

H. Variation in complement factor H affects complement activation in

immunoglobulin A vasculitis with nephritis. Nephrology (Carlton). 2020

Jan;25(1):40-47. doi: 10.1111/nep.13580. Epub 2019 May 5. PMID: 30838755.

202: Rohrer B, Frazer-Abel A, Leonard A, Ratnapriya R, Ward T, Pietraszkiewicz

A, O'Quinn E, Adams K, Swaroop A, Wolf BJ. Association of age-related macular

degeneration with complement activation products, smoking, and single nucleotide

polymorphisms in South Carolinians of European and African descent. Mol Vis.

2019 Feb 8;25:79-92. PMID: 30820144; PMCID: PMC6377374.

203: Pouw RB, Brouwer MC, de Gast M, van Beek AE, van den Heuvel LP, Schmidt CQ,

van der Ende A, Sánchez-Corral P, Kuijpers TW, Wouters D. Potentiation of

complement regulator factor H protects human endothelial cells from complement

attack in aHUS sera. Blood Adv. 2019 Feb 26;3(4):621-632. doi:

10.1182/bloodadvances.2018025692. PMID: 30804016; PMCID: PMC6391659.

204: Yin C, Ackermann S, Ma Z, Mohanta SK, Zhang C, Li Y, Nietzsche S,

Westermann M, Peng L, Hu D, Bontha SV, Srikakulapu P, Beer M, Megens RTA,

Steffens S, Hildner M, Halder LD, Eckstein HH, Pelisek J, Herms J, Roeber S,

Arzberger T, Borodovsky A, Habenicht L, Binder CJ, Weber C, Zipfel PF, Skerka C,

Habenicht AJR. ApoE attenuates unresolvable inflammation by complex formation

with activated C1q. Nat Med. 2019 Mar;25(3):496-506. doi:

10.1038/s41591-018-0336-8. Epub 2019 Jan 28. Erratum in: Nat Med. 2019 Feb 4;:

PMID: 30692699; PMCID: PMC6420126.

205: Raymond W, Eilertsen G, Nossent J. Hypocomplementemia as a Risk Factor for

Organ Damage Accrual in Patients with Systemic Lupus Erythematosus. J Immunol

Res. 2018 Dec 30;2018:8051972. doi: 10.1155/2018/8051972. PMID: 30687766; PMCID:

PMC6330819.

206: Wilson HR, Medjeral-Thomas NR, Gilmore AC, Trivedi P, Seyb K, Farzaneh-Far

R, Gunnarsson I, Zickert A, Cairns TD, Lightstone L, Cook HT, Pickering MC.

Glomerular membrane attack complex is not a reliable marker of ongoing C5

activation in lupus nephritis. Kidney Int. 2019 Mar;95(3):655-665. doi:

10.1016/j.kint.2018.09.027. Epub 2019 Jan 14. PMID: 30655025; PMCID: PMC6389546.

207: Heesterbeek DA, Bardoel BW, Parsons ES, Bennett I, Ruyken M, Doorduijn DJ,

Gorham RD Jr, Berends ET, Pyne AL, Hoogenboom BW, Rooijakkers SH. Bacterial

killing by complement requires membrane attack complex formation via surface-

bound C5 convertases. EMBO J. 2019 Feb 15;38(4):e99852. doi:

10.15252/embj.201899852. Epub 2019 Jan 14. PMID: 30643019; PMCID: PMC6376327.

208: May O, Merle NS, Grunenwald A, Gnemmi V, Leon J, Payet C, Robe-Rybkine T,

Paule R, Delguste F, Satchell SC, Mathieson PW, Hazzan M, Boulanger E, Dimitrov

JD, Fremeaux-Bacchi V, Frimat M, Roumenina LT. Heme Drives Susceptibility of

Glomerular Endothelium to Complement Overactivation Due to Inefficient

Upregulation of Heme Oxygenase-1. Front Immunol. 2018 Dec 20;9:3008. doi:

10.3389/fimmu.2018.03008. PMID: 30619356; PMCID: PMC6306430.

209: Michelis R, Tadmor T, Barhoum M, Shehadeh M, Shvidel L, Aviv A, Stemer G,

Dally N, Rahimi-Levene N, Yuklea M, Braester A. A C5a-Immunoglobulin complex in

chronic lymphocytic leukemia patients is associated with decreased complement

activity. PLoS One. 2019 Jan 2;14(1):e0209024. doi:

10.1371/journal.pone.0209024. PMID: 30601845; PMCID: PMC6314568.

210: Dalili N, Behnam B, Vali F, Parvin M, Torbati P, Rasaii N, Samadian F,

Ahmadpoor P. C3 Glomerulonephritis With Multiple Mutations in Complement Factor

H. Iran J Kidney Dis. 2018 Nov;12(6):376-381. PMID: 30595568.

211: Sampei Z, Haraya K, Tachibana T, Fukuzawa T, Shida-Kawazoe M, Gan SW,

Shimizu Y, Ruike Y, Feng S, Kuramochi T, Muraoka M, Kitazawa T, Kawabe Y, Igawa

T, Hattori K, Nezu J. Antibody engineering to generate SKY59, a long-acting

anti-C5 recycling antibody. PLoS One. 2018 Dec 28;13(12):e0209509. doi:

10.1371/journal.pone.0209509. PMID: 30592762; PMCID: PMC6310256.

212: Mutti M, Ramoni K, Nagy G, Nagy E, Szijártó V. A New Tool for Complement

Research: <i>In vitro</i> Reconstituted Human Classical Complement Pathway.

Front Immunol. 2018 Dec 4;9:2770. doi: 10.3389/fimmu.2018.02770. PMID: 30564230;

PMCID: PMC6288441.

213: Madden I, Roumenina LT, Langlois-Meurinne H, Guichoux J, Llanas B,

Frémeaux-Bacchi V, Harambat J, Godron-Dubrasquet A. Hemolytic uremic syndrome

associated with Bordetella pertussis infection in a 2-month-old infant carrying

a pathogenic variant in complement factor H. Pediatr Nephrol. 2019

Mar;34(3):533-537. doi: 10.1007/s00467-018-4174-1. Epub 2018 Dec 17. PMID:

30560448.

214: Rajan P, Mishra PKK, Joshi P. Defining the complement C3 binding site and

the antigenic region of Haemonchus contortus GAPDH. Parasite Immunol. 2019

Feb;41(2):e12611. doi: 10.1111/pim.12611. PMID: 30548600.

215: Chiorean RM, Baican A, Mustafa MB, Lischka A, Leucuta DC, Feldrihan V,

Hertl M, Sitaru C. Complement-Activating Capacity of Autoantibodies Correlates

With Disease Activity in Bullous Pemphigoid Patients. Front Immunol. 2018 Nov

20;9:2687. doi: 10.3389/fimmu.2018.02687. PMID: 30524436; PMCID: PMC6257046.

216: Zha H, Wang X, Zhu Y, Chen D, Han X, Yang F, Gao J, Hu C, Shu C, Feng Y,

Tan Y, Zhang J, Li Y, Wan YY, Guo B, Zhu B. Intracellular Activation of

Complement C3 Leads to PD-L1 Antibody Treatment Resistance by Modulating Tumor-

Associated Macrophages. Cancer Immunol Res. 2019 Feb;7(2):193-207. doi:

10.1158/2326-6066.CIR-18-0272. Epub 2018 Dec 4. PMID: 30514794.

217: Umnyakova ES, Gorbunov NP, Zhakhov AV, Krenev IA, Ovchinnikova TV,

Kokryakov VN, Berlov MN. Modulation of Human Complement System by Antimicrobial

Peptide Arenicin-1 from <i>Arenicola marina</i>. Mar Drugs. 2018 Dec

1;16(12):480. doi: 10.3390/md16120480. PMID: 30513754; PMCID: PMC6315390.

218: Reiss T, Rosa TFA, Blaesius K, Bobbert RP, Zipfel PF, Skerka C, Pradel G.

Cutting Edge: FHR-1 Binding Impairs Factor H-Mediated Complement Evasion by the

Malaria Parasite <i>Plasmodium falciparum</i>. J Immunol. 2018 Dec

15;201(12):3497-3502. doi: 10.4049/jimmunol.1800662. Epub 2018 Nov 19. PMID:

30455399.

219: Hu C, Li L, Ding P, Li L, Ge X, Zheng L, Wang X, Wang J, Zhang W, Wang N,

Gu H, Zhong F, Xu M, Rong R, Zhu T, Hu W. Complement Inhibitor CRIg/FH

Ameliorates Renal Ischemia Reperfusion Injury via Activation of PI3K/AKT

Signaling. J Immunol. 2018 Dec 15;201(12):3717-3730. doi:

10.4049/jimmunol.1800987. Epub 2018 Nov 14. PMID: 30429287; PMCID: PMC6287101.

220: Burwick RM, Velásquez JA, Valencia CM, Gutiérrez-Marín J, Edna-Estrada F,

Silva JL, Trujillo-Otálvaro J, Vargas-Rodríguez J, Bernal Y, Quintero A, Rincón

M, Tolosa JE. Terminal Complement Activation in Preeclampsia. Obstet Gynecol.

2018 Dec;132(6):1477-1485. doi: 10.1097/AOG.0000000000002980. PMID: 30399106.

221: Zhang J, Ye J, Ren Y, Zuo J, Dai W, He Y, Tan M, Song W, Yuan Y.

Intracellular activation of complement C3 in Paneth cells improves repair of

intestinal epithelia during acute injury. Immunotherapy. 2018

Nov;10(15):1325-1336. doi: 10.2217/imt-2018-0122. Epub 2018 Nov 1. PMID:

30381988.

222: Rodríguez E, Gimeno J, Arias-Cabrales C, Barrios C, Redondo-Pachón D, Soler

MJ, Crespo M, Sierra-Ochoa A, Riera M, Pascual J. Membrane Attack Complex and

Factor H in Humans with Acute Kidney Injury. Kidney Blood Press Res.

2018;43(5):1655-1665. doi: 10.1159/000494680. Epub 2018 Oct 31. PMID: 30380547.

223: Saez D, Dushime R, Wu H, Ramos Cordova LB, Shukla K, Brown-Harding H,

Furdui CM, Tsang AW. Sulforaphane promotes chlamydial infection by suppressing

mitochondrial protein oxidation and activation of complement C3. Protein Sci.

2019 Jan;28(1):216-227. doi: 10.1002/pro.3536. PMID: 30367535; PMCID:

PMC6296177.

224: Mészáros T, Kozma GT, Shimizu T, Miyahara K, Turjeman K, Ishida T,

Barenholz Y, Urbanics R, Szebeni J. Involvement of complement activation in the

pulmonary vasoactivity of polystyrene nanoparticles in pigs: unique surface

properties underlying alternative pathway activation and instant opsonization.

Int J Nanomedicine. 2018 Oct 11;13:6345-6357. doi: 10.2147/IJN.S161369. PMID:

30349254; PMCID: PMC6187999.

225: Chauvet S, Roumenina LT, Aucouturier P, Marinozzi MC, Dragon-Durey MA,

Karras A, Delmas Y, Le Quintrec M, Guerrot D, Jourde-Chiche N, Ribes D, Ronco P,

Bridoux F, Fremeaux-Bacchi V. Both Monoclonal and Polyclonal Immunoglobulin

Contingents Mediate Complement Activation in Monoclonal Gammopathy Associated-C3

Glomerulopathy. Front Immunol. 2018 Oct 2;9:2260. doi: 10.3389/fimmu.2018.02260.

PMID: 30333829; PMCID: PMC6175995.

226: O'Flynn J, Kotimaa J, Faber-Krol R, Koekkoek K, Klar-Mohamad N, Koudijs A,

Schwaeble WJ, Stover C, Daha MR, van Kooten C. Properdin binds independent of

complement activation in an in vivo model of anti-glomerular basement membrane

disease. Kidney Int. 2018 Dec;94(6):1141-1150. doi: 10.1016/j.kint.2018.06.030.

Epub 2018 Oct 12. PMID: 30322716.

227: Khandelwal S, Ravi J, Rauova L, Johnson A, Lee GM, Gilner JB, Gunti S,

Notkins AL, Kuchibhatla M, Frank M, Poncz M, Cines DB, Arepally GM. Polyreactive

IgM initiates complement activation by PF4/heparin complexes through the

classical pathway. Blood. 2018 Dec 6;132(23):2431-2440. doi:

10.1182/blood-2018-03-834598. Epub 2018 Oct 11. PMID: 30309891; PMCID:

PMC6284214.

228: Poppelaars F, Gaya da Costa M, Faria B, Berger SP, Assa S, Daha MR, Medina

Pestana JO, van Son WJ, Franssen CFM, Seelen MA. Intradialytic Complement

Activation Precedes the Development of Cardiovascular Events in Hemodialysis

Patients. Front Immunol. 2018 Sep 13;9:2070. doi: 10.3389/fimmu.2018.02070.

PMID: 30271407; PMCID: PMC6146103.

229: Krilis M, Qi M, Qi J, Wong JWH, Guymer R, Liew G, Hunyor AP, Madigan M,

McCluskey P, Weaver J, Krilis SA, Giannakopoulos B. Dual roles of different

redox forms of complement factor H in protecting against age related macular

degeneration. Free Radic Biol Med. 2018 Dec;129:237-246. doi:

10.1016/j.freeradbiomed.2018.09.034. Epub 2018 Sep 22. PMID: 30253188.

230: Karnisova L, Hradsky O, Blahova K, Fencl F, Dolezel Z, Zaoral T, Zieg J.

Complement activation is associated with more severe course of diarrhea-

associated hemolytic uremic syndrome, a preliminary study. Eur J Pediatr. 2018

Dec;177(12):1837-1844. doi: 10.1007/s00431-018-3255-2. Epub 2018 Sep 24. PMID:

30251107.

231: Zhao Y, Luo C, Chen J, Sun Y, Pu D, Lv A, Zhu S, Wu J, Wang M, Zhou J, Liao

Z, Zhao K, Xiao Q. High glucose-induced complement component 3 up-regulation via

RAGE-p38MAPK-NF-κB signalling in astrocytes: In vivo and in vitro studies. J

Cell Mol Med. 2018 Dec;22(12):6087-6098. doi: 10.1111/jcmm.13884. Epub 2018 Sep

24. PMID: 30246940; PMCID: PMC6237571.

232: Nakano D, Nishiyama A. A novel role of renin inhibitor in the complement

cascade. Kidney Int. 2018 Oct;94(4):650-652. doi: 10.1016/j.kint.2018.05.025.

PMID: 30243307.

233: Zheng JM, Jiang ZH, Chen DJ, Wang SS, Zhao WJ, Li LJ. Pathological

significance of urinary complement activation in diabetic nephropathy: A full

view from the development of the disease. J Diabetes Investig. 2019

May;10(3):738-744. doi: 10.1111/jdi.12934. Epub 2018 Oct 13. PMID: 30239170;

PMCID: PMC6497774.

234: Holle J, Berenberg-Goßler L, Wu K, Beringer O, Kropp F, Müller D, Thumfart

J. Outcome of membranoproliferative glomerulonephritis and C3-glomerulopathy in

children and adolescents. Pediatr Nephrol. 2018 Dec;33(12):2289-2298. doi:

10.1007/s00467-018-4034-z. Epub 2018 Sep 20. PMID: 30238151.

235: Wang B, Xu H, Li J, Gao HM, Xing YH, Lin Z, Li HJ, Wang YQ, Cao SH.

Complement depletion with cobra venom factor alleviates acute hepatic injury

induced by ischemia‑reperfusion. Mol Med Rep. 2018 Nov;18(5):4523-4529. doi:

10.3892/mmr.2018.9484. Epub 2018 Sep 14. PMID: 30221740; PMCID: PMC6172365.

236: Guo WY, Liu QZ, Zhu L, Li ZY, Meng SJ, Shi SF, Liu LJ, Lv JC, Hou P, Zhang

H. Coding and Noncoding Variants in CFH Act Synergistically for Complement

Activation in Immunoglobulin A Nephropathy. Am J Med Sci. 2018

Aug;356(2):114-120. doi: 10.1016/j.amjms.2018.04.006. Epub 2018 Apr 11. PMID:

30219152.

237: Rossi O, Coward C, Goh YS, Claassens JWC, MacLennan CA, Verbeek SJ,

Mastroeni P. The essential role of complement in antibody-mediated resistance to

Salmonella. Immunology. 2019 Jan;156(1):69-73. doi: 10.1111/imm.13000. Epub 2018

Oct 10. PMID: 30179254; PMCID: PMC6283648.

238: Michels MAHM, Volokhina EB, van de Kar NCAJ, van den Heuvel LPWJ. The role

of properdin in complement-mediated renal diseases: a new player in complement-

inhibiting therapy? Pediatr Nephrol. 2019 Aug;34(8):1349-1367. doi:

10.1007/s00467-018-4042-z. Epub 2018 Aug 23. PMID: 30141176; PMCID: PMC6579773.

239: Meinshausen AK, Märtens N, Berth A, Färber J, Awiszus F, Macor P, Lohmann

CH, Bertrand J. The terminal complement pathway is activated in septic but not

in aseptic shoulder revision arthroplasties. J Shoulder Elbow Surg. 2018

Oct;27(10):1837-1844. doi: 10.1016/j.jse.2018.06.037. Epub 2018 Aug 20. PMID:

30139682.

240: Li Y, Yang Z, Chavko M, Liu B, Aderemi OA, Simovic MO, Dubick MA, Cancio

LC. Complement inhibition ameliorates blast-induced acute lung injury in rats:

Potential role of complement in intracellular HMGB1-mediated inflammation. PLoS

One. 2018 Aug 22;13(8):e0202594. doi: 10.1371/journal.pone.0202594. PMID:

30133517; PMCID: PMC6105023.

241: Mohlin FC, Gros P, Mercier E, Gris JR, Blom AM. Analysis of C3 Gene

Variants in Patients With Idiopathic Recurrent Spontaneous Pregnancy Loss. Front

Immunol. 2018 Aug 7;9:1813. doi: 10.3389/fimmu.2018.01813. PMID: 30131807;

PMCID: PMC6090058.

242: Banadakoppa M, Balakrishnan M, Yallampalli C. Upregulation and release of

soluble fms-like tyrosine kinase receptor 1 mediated by complement activation in

human syncytiotrophoblast cells. Am J Reprod Immunol. 2018 Nov;80(5):e13033.

doi: 10.1111/aji.13033. Epub 2018 Aug 12. PMID: 30099798; PMCID: PMC6202180.

243: Farrokhi Yekta R, Arefi Oskouie A, Rezaei Tavirani M, Mohajeri-Tehrani MR,

Soroush AR. Decreased apolipoprotein A4 and increased complement component 3 as

potential markers for papillary thyroid carcinoma: A proteomic study. Int J Biol

Markers. 2018 Nov;33(4):455-462. doi: 10.1177/1724600818787752. Epub 2018 Jul

30. PMID: 30058426.

244: Bartoszek D, Mazanowska O, Kościelska-Kasprzak K, Kamińska D, Lepiesza A,

Chudoba P, Myszka M, Żabińska M, Klinger M. Functional Activity of the

Complement System in Deceased Donors in Relation to Kidney Allograft Outcome.

Transplant Proc. 2018 Jul-Aug;50(6):1697-1700. doi:

10.1016/j.transproceed.2018.02.157. Epub 2018 Mar 15. PMID: 30056884.

245: Wang R, He D, Zhao L, Liang S, Liang D, Xu F, Zhang M, Zhu X, Chen H, Xie

H, Zeng C, Tang Z, Liu Z. Role of complement system in patients with biopsy-

proven immunoglobulin G4-related kidney disease. Hum Pathol. 2018

Nov;81:220-228. doi: 10.1016/j.humpath.2018.07.008. Epub 2018 Jul 18. PMID:

30031099.

246: Jalal D, Renner B, Laskowski J, Stites E, Cooper J, Valente K, You Z,

Perrenoud L, Le Quintrec M, Muhamed I, Christians U, Klawitter J, Lindorfer MA,

Taylor RP, Holers VM, Thurman JM. Endothelial Microparticles and Systemic

Complement Activation in Patients With Chronic Kidney Disease. J Am Heart Assoc.

2018 Jul 13;7(14):e007818. doi: 10.1161/JAHA.117.007818. PMID: 30006493; PMCID:

PMC6064828.

247: Khandelwal S, Johnson AM, Liu J, Keire D, Sommers C, Ravi J, Lee GM,

Lambris JD, Reis ES, Arepally GM. Novel Immunoassay for Complement Activation by

PF4/Heparin Complexes. Thromb Haemost. 2018 Aug;118(8):1484-1487. doi:

10.1055/s-0038-1660858. Epub 2018 Jun 30. PMID: 29960275; PMCID: PMC6288012.

248: Fernandez-Godino R, Pierce EA. C3a triggers formation of sub-retinal

pigment epithelium deposits via the ubiquitin proteasome pathway. Sci Rep. 2018

Jun 26;8(1):9679. doi: 10.1038/s41598-018-28143-0. PMID: 29946065; PMCID:

PMC6018664.

249: Willrich MAV, Andreguetto BD, Sridharan M, Fervenza FC, Tostrud LJ, Ladwig

PM, Rivard AM, Hetrick MD, Olson RN, Bryant SC, Snyder MR, Murray DL. The impact

of eculizumab on routine complement assays. J Immunol Methods. 2018

Sep;460:63-71. doi: 10.1016/j.jim.2018.06.010. Epub 2018 Jun 20. PMID: 29935209.

250: Daha MR, Seelen M. Novel Approaches to Control of the Alternative

Complement Pathway for the Treatment of C3 Glomerulopathies. J Am Soc Nephrol.

2018 Aug;29(8):2032-2033. doi: 10.1681/ASN.2018050554. Epub 2018 Jun 19. PMID:

29921719; PMCID: PMC6065089.

251: Alawieh A, Andersen M, Adkins DL, Tomlinson S. Acute Complement Inhibition

Potentiates Neurorehabilitation and Enhances tPA-Mediated Neuroprotection. J

Neurosci. 2018 Jul 18;38(29):6527-6545. doi: 10.1523/JNEUROSCI.0111-18.2018.

Epub 2018 Jun 19. PMID: 29921716; PMCID: PMC6052238.

252: Zhang Q, Hickey M, Drogalis-Kim D, Zheng Y, Gjertson D, Cadeiras M, Khuu T,

Baas AS, Depasquale EC, Halnon NJ, Perens G, Alejos J, Cruz D, Ali N, Shemin R,

Kwon M, Fishbein MC, Ardehali A, Deng M, Reed EF. Understanding the Correlation

Between DSA, Complement Activation, and Antibody-Mediated Rejection in Heart

Transplant Recipients. Transplantation. 2018 Oct;102(10):e431-e438. doi:

10.1097/TP.0000000000002333. PMID: 29916988; PMCID: PMC6153056.

253: Budkowska M, Ostrycharz E, Wojtowicz A, Marcinowska Z, Woźniak J, Ratajczak

MZ, Dołęgowska B. A Circadian Rhythm in both Complement Cascade (ComC)

Activation and Sphingosine-1-Phosphate (S1P) Levels in Human Peripheral Blood

Supports a Role for the ComC-S1P Axis in Circadian Changes in the Number of Stem

Cells Circulating in Peripheral Blood. Stem Cell Rev Rep. 2018

Oct;14(5):677-685. doi: 10.1007/s12015-018-9836-7. PMID: 29911288; PMCID:

PMC6132735.

254: Bavia L, Lidani KCF, Andrade FA, Sobrinho MIAH, Nisihara RM, de Messias-

Reason IJ. Complement activation in acute myocardial infarction: An early marker

of inflammation and tissue injury? Immunol Lett. 2018 Aug;200:18-25. doi:

10.1016/j.imlet.2018.06.006. Epub 2018 Jun 18. PMID: 29908956.

255: Ma H, Liu C, Shi B, Zhang Z, Feng R, Guo M, Lu L, Shi S, Gao X, Chen W, Sun

L. Mesenchymal Stem Cells Control Complement C5 Activation by Factor H in Lupus

Nephritis. EBioMedicine. 2018 Jun;32:21-30. doi: 10.1016/j.ebiom.2018.05.034.

Epub 2018 Jun 7. PMID: 29885865; PMCID: PMC6020800.

256: Békássy ZD, Kristoffersson AC, Rebetz J, Tati R, Olin AI, Karpman D.

Aliskiren inhibits renin-mediated complement activation. Kidney Int. 2018

Oct;94(4):689-700. doi: 10.1016/j.kint.2018.04.004. Epub 2018 Jun 5. PMID:

29884545.

257: Umehara H, Kawano M. Response to: 'Serum complement factor C5a in

IgG4-related disease' by Fukui <i>et al</i>. Ann Rheum Dis. 2019 Jul;78(7):e66.

doi: 10.1136/annrheumdis-2018-213729. Epub 2018 Jun 6. PMID: 29875098.

258: Deng S, Xu T, Fang Q, Yu L, Zhu J, Chen L, Liu J, Zhou R. The Surface-

Exposed Protein SntA Contributes to Complement Evasion in Zoonotic

<i>Streptococcus suis</i>. Front Immunol. 2018 May 16;9:1063. doi:

10.3389/fimmu.2018.01063. PMID: 29868022; PMCID: PMC5964162.

259: Timmermans SAMEG, Abdul-Hamid MA, Potjewijd J, Theunissen ROMFIH,

Damoiseaux JGMC, Reutelingsperger CP, van Paassen P; Limburg Renal Registry.

C5b9 Formation on Endothelial Cells Reflects Complement Defects among Patients

with Renal Thrombotic Microangiopathy and Severe Hypertension. J Am Soc Nephrol.

2018 Aug;29(8):2234-2243. doi: 10.1681/ASN.2018020184. Epub 2018 Jun 1. PMID:

29858281; PMCID: PMC6065094.

260: Sauter RJ, Sauter M, Reis ES, Emschermann FN, Nording H, Ebenhöch S, Kraft

P, Münzer P, Mauler M, Rheinlaender J, Madlung J, Edlich F, Schäffer TE, Meuth

SG, Duerschmied D, Geisler T, Borst O, Gawaz M, Kleinschnitz C, Lambris JD,

Langer HF. Functional Relevance of the Anaphylatoxin Receptor C3aR for Platelet

Function and Arterial Thrombus Formation Marks an Intersection Point Between

Innate Immunity and Thrombosis. Circulation. 2018 Oct 16;138(16):1720-1735. doi:

10.1161/CIRCULATIONAHA.118.034600. Erratum in: Circulation. 2019 Jan

15;139(3):e8. Dürschmied, Daniel [corrected to Duerschmied, Daniel]. PMID:

29802205; PMCID: PMC6202244.

261: Zhu L, Guo WY, Shi SF, Liu LJ, Lv JC, Medjeral-Thomas NR, Lomax-Browne HJ,

Pickering MC, Zhang H. Circulating complement factor H-related protein 5 levels

contribute to development and progression of IgA nephropathy. Kidney Int. 2018

Jul;94(1):150-158. doi: 10.1016/j.kint.2018.02.023. Epub 2018 May 11. PMID:

29759419.

262: Tavano R, Gabrielli L, Lubian E, Fedeli C, Visentin S, Polverino De Laureto

P, Arrigoni G, Geffner-Smith A, Chen F, Simberg D, Morgese G, Benetti EM, Wu L,

Moghimi SM, Mancin F, Papini E. C1q-Mediated Complement Activation and C3

Opsonization Trigger Recognition of Stealth Poly(2-methyl-2-oxazoline)-Coated

Silica Nanoparticles by Human Phagocytes. ACS Nano. 2018 Jun 26;12(6):5834-5847.

doi: 10.1021/acsnano.8b01806. Epub 2018 May 23. PMID: 29750504; PMCID:

PMC6251765.

263: Katschke KJ Jr, Xi H, Cox C, Truong T, Malato Y, Lee WP, McKenzie B, Arceo

R, Tao J, Rangell L, Reichelt M, Diehl L, Elstrott J, Weimer RM, van Lookeren

Campagne M. Classical and alternative complement activation on photoreceptor

outer segments drives monocyte-dependent retinal atrophy. Sci Rep. 2018 May

9;8(1):7348. doi: 10.1038/s41598-018-25557-8. Erratum in: Sci Rep. 2018 Aug

24;8(1):13055. PMID: 29743491; PMCID: PMC5943270.

264: Ravindran A, Fervenza FC, Smith RJH, Sethi S. C3 glomerulopathy associated

with monoclonal Ig is a distinct subtype. Kidney Int. 2018 Jul;94(1):178-186.

doi: 10.1016/j.kint.2018.01.037. Epub 2018 May 3. Erratum in: Kidney Int. 2018

Nov;94(5):1025. PMID: 29729982; PMCID: PMC7735221.

265: Fernandez-Godino R. Alterations in Extracellular Matrix/Bruch's Membrane

Can Cause the Activation of the Alternative Complement Pathway via Tick-Over.

Adv Exp Med Biol. 2018;1074:29-35. doi: 10.1007/978-3-319-75402-4_4. PMID:

29721924.

266: Jiang Y, Zhao G, Song N, Li P, Chen Y, Guo Y, Li J, Du L, Jiang S, Guo R,

Sun S, Zhou Y. Blockade of the C5a-C5aR axis alleviates lung damage in

hDPP4-transgenic mice infected with MERS-CoV. Emerg Microbes Infect. 2018 Apr

24;7(1):77. doi: 10.1038/s41426-018-0063-8. PMID: 29691378; PMCID: PMC5915580.

267: Zheng JM, Ren XG, Jiang ZH, Chen DJ, Zhao WJ, Li LJ. Lectin-induced renal

local complement activation is involved in tubular interstitial injury in

diabetic nephropathy. Clin Chim Acta. 2018 Jul;482:65-73. doi:

10.1016/j.cca.2018.03.033. Epub 2018 Mar 28. PMID: 29604259.

268: Percheron L, Gramada R, Tellier S, Salomon R, Harambat J, Llanas B, Fila M,

Allain-Launay E, Lapeyraque AL, Leroy V, Adra AL, Bérard E, Bourdat-Michel G,

Chehade H, Eckart P, Merieau E, Piètrement C, Sellier-Leclerc AL, Frémeaux-

Bacchi V, Dimeglio C, Garnier A. Eculizumab treatment in severe pediatric STEC-

HUS: a multicenter retrospective study. Pediatr Nephrol. 2018

Aug;33(8):1385-1394. doi: 10.1007/s00467-018-3903-9. Epub 2018 Mar 23. PMID:

29572749.

269: Sartain SE, Turner NA, Moake JL. Brain microvascular endothelial cells

exhibit lower activation of the alternative complement pathway than glomerular

microvascular endothelial cells. J Biol Chem. 2018 May 11;293(19):7195-7208.

doi: 10.1074/jbc.RA118.002639. Epub 2018 Mar 19. PMID: 29555686; PMCID:

PMC5949983.

270: Abu-Humaidan AH, Elvén M, Sonesson A, Garred P, Sørensen OE. Persistent

Intracellular <i>Staphylococcus aureus</i> in Keratinocytes Lead to Activation

of the Complement System with Subsequent Reduction in the Intracellular

Bacterial Load. Front Immunol. 2018 Mar 1;9:396. doi: 10.3389/fimmu.2018.00396.

PMID: 29545804; PMCID: PMC5837974.

271: Roux A, Thomas KA, Sage E, Suberbielle-Boissel C, Beaumont-Azuar L, Parquin

F, Le Guen M, Harre N, Hamid AM, Reed EF. Donor-specific HLA antibody-mediated

complement activation is a significant indicator of antibody-mediated rejection

and poor long-term graft outcome during lung transplantation: a single center

cohort study. Transpl Int. 2018 Jul;31(7):761-772. doi: 10.1111/tri.13149. Epub

2018 Apr 22. PMID: 29537702; PMCID: PMC5995657.

272: Gorbushin AM. Immune repertoire in the transcriptome of Littorina littorea

reveals new trends in lophotrochozoan proto-complement evolution. Dev Comp

Immunol. 2018 Jul;84:250-263. doi: 10.1016/j.dci.2018.02.018. Epub 2018 Feb 28.

PMID: 29501422.

273: Osborne AJ, Breno M, Borsa NG, Bu F, Frémeaux-Bacchi V, Gale DP, van den

Heuvel LP, Kavanagh D, Noris M, Pinto S, Rallapalli PM, Remuzzi G, Rodríguez de

Cordoba S, Ruiz A, Smith RJH, Vieira-Martins P, Volokhina E, Wilson V, Goodship

THJ, Perkins SJ. Statistical Validation of Rare Complement Variants Provides

Insights into the Molecular Basis of Atypical Hemolytic Uremic Syndrome and C3

Glomerulopathy. J Immunol. 2018 Apr 1;200(7):2464-2478. doi:

10.4049/jimmunol.1701695. Epub 2018 Mar 2. PMID: 29500241; PMCID: PMC6324840.

274: Jensen RK, Pihl R, Gadeberg TAF, Jensen JK, Andersen KR, Thiel S, Laursen

NS, Andersen GR. A potent complement factor C3-specific nanobody inhibiting

multiple functions in the alternative pathway of human and murine complement. J

Biol Chem. 2018 Apr 27;293(17):6269-6281. doi: 10.1074/jbc.RA117.001179. Epub

2018 Mar 1. PMID: 29497000; PMCID: PMC5925797.

275: Thomas AM, Schjalm C, Nilsson PH, Lindenskov PHH, Rørtveit R, Solberg R,

Saugstad OD, Berglund MM, Strömberg P, Lau C, Espevik T, Jansen JH, Castellheim

A, Mollnes TE, Barratt-Due A. Combined Inhibition of C5 and CD14 Attenuates

Systemic Inflammation in a Piglet Model of Meconium Aspiration Syndrome.

Neonatology. 2018;113(4):322-330. doi: 10.1159/000486542. Epub 2018 Feb 27.

PMID: 29486477; PMCID: PMC6008878.

276: Rudnick RB, Chen Q, Stea ED, Hartmann A, Papac-Milicevic N, Person F,

Wiesener M, Binder CJ, Wiech T, Skerka C, Zipfel PF. FHR5 Binds to Laminins,

Uses Separate C3b and Surface-Binding Sites, and Activates Complement on

Malondialdehyde-Acetaldehyde Surfaces. J Immunol. 2018 Apr 1;200(7):2280-2290.

doi: 10.4049/jimmunol.1701641. Epub 2018 Feb 26. PMID: 29483359.

277: Espinosa-Figueroa JL, Cano-Megías M, Martínez-Miguel P, Velo-Plaza M.

Recurrent postinfectious glomerulonephritis: an unusual evolution compatible

with C3 glomerulopathy. BMJ Case Rep. 2018 Feb 23;2018:bcr2017222979. doi:

10.1136/bcr-2017-222979. PMID: 29477994; PMCID: PMC5847972.

278: Paréj K, Kocsis A, Enyingi C, Dani R, Oroszlán G, Beinrohr L, Dobó J,

Závodszky P, Pál G, Gál P. Cutting Edge: A New Player in the Alternative

Complement Pathway, MASP-1 Is Essential for LPS-Induced, but Not for Zymosan-

Induced, Alternative Pathway Activation. J Immunol. 2018 Apr 1;200(7):2247-2252.

doi: 10.4049/jimmunol.1701421. Epub 2018 Feb 23. PMID: 29475986.

279: Skjeflo EW, Christiansen D, Fure H, Ludviksen JK, Woodruff TM, Espevik T,

Nielsen EW, Brekke OL, Mollnes TE. Staphylococcus aureus-induced complement

activation promotes tissue factor-mediated coagulation. J Thromb Haemost. 2018

May;16(5):905-918. doi: 10.1111/jth.13979. Epub 2018 Mar 23. PMID: 29437288.

280: De Blasio D, Fumagalli S, Orsini F, Neglia L, Perego C, Ortolano F, Zanier

ER, Picetti E, Locatelli M, Stocchetti N, Longhi L, Garred P, De Simoni MG.

Human brain trauma severity is associated with lectin complement pathway

activation. J Cereb Blood Flow Metab. 2019 May;39(5):794-807. doi:

10.1177/0271678X18758881. Epub 2018 Feb 9. PMID: 29425056; PMCID: PMC6501516.

281: Kanni T, Zenker O, Habel M, Riedemann N, Giamarellos-Bourboulis EJ.

Complement activation in hidradenitis suppurativa: a new pathway of

pathogenesis? Br J Dermatol. 2018 Aug;179(2):413-419. doi: 10.1111/bjd.16428.

Epub 2018 May 10. PMID: 29405257.

282: Lorés-Motta L, Paun CC, Corominas J, Pauper M, Geerlings MJ, Altay L,

Schick T, Daha MR, Fauser S, Hoyng CB, den Hollander AI, de Jong EK. Genome-Wide

Association Study Reveals Variants in CFH and CFHR4 Associated with Systemic

Complement Activation: Implications in Age-Related Macular Degeneration.

Ophthalmology. 2018 Jul;125(7):1064-1074. doi: 10.1016/j.ophtha.2017.12.023.

Epub 2018 Feb 2. PMID: 29398083.

283: Page MJ, Bester J, Pretorius E. The inflammatory effects of TNF-α and

complement component 3 on coagulation. Sci Rep. 2018 Jan 29;8(1):1812. doi:

10.1038/s41598-018-20220-8. PMID: 29379088; PMCID: PMC5789054.

284: Kim MY, Guerra MM, Kaplowitz E, Laskin CA, Petri M, Branch DW, Lockshin MD,

Sammaritano LR, Merrill JT, Porter TF, Sawitzke A, Lynch AM, Buyon JP, Salmon

JE. Complement activation predicts adverse pregnancy outcome in patients with

systemic lupus erythematosus and/or antiphospholipid antibodies. Ann Rheum Dis.

2018 Apr;77(4):549-555. doi: 10.1136/annrheumdis-2017-212224. Epub 2018 Jan 25.

PMID: 29371202; PMCID: PMC6037302.

285: Ren W, Liu Y, Wang X, Piao C, Ma Y, Qiu S, Jia L, Chen B, Wang Y, Jiang W,

Zheng S, Liu C, Dai N, Lan F, Zhang H, Song WC, Du J. The Complement

C3a<i>-</i>C3aR Axis Promotes Development of Thoracic Aortic Dissection via

Regulation of MMP2 Expression. J Immunol. 2018 Mar 1;200(5):1829-1838. doi:

10.4049/jimmunol.1601386. Epub 2018 Jan 24. PMID: 29367209.

286: Biesecker SG, Nicastro LK, Wilson RP, Tükel Ç. The Functional Amyloid Curli

Protects Escherichia coli against Complement-Mediated Bactericidal Activity.

Biomolecules. 2018 Jan 24;8(1):5. doi: 10.3390/biom8010005. PMID: 29364839;

PMCID: PMC5871974.

287: Zhu X, Zhang J, Wang Q, Fu H, Chang Y, Kong Y, Lv M, Xu L, Liu K, Huang X,

Zhang X. Diminished expression of β2-GPI is associated with a reduced ability to

mitigate complement activation in anti-GPIIb/IIIa-mediated immune

thrombocytopenia. Ann Hematol. 2018 Apr;97(4):641-654. doi:

10.1007/s00277-017-3215-3. Epub 2017 Dec 29. PMID: 29350259.

288: Bomback AS, Santoriello D, Avasare RS, Regunathan-Shenk R, Canetta PA, Ahn

W, Radhakrishnan J, Marasa M, Rosenstiel PE, Herlitz LC, Markowitz GS, D'Agati

VD, Appel GB. C3 glomerulonephritis and dense deposit disease share a similar

disease course in a large United States cohort of patients with C3

glomerulopathy. Kidney Int. 2018 Apr;93(4):977-985. doi:

10.1016/j.kint.2017.10.022. Epub 2018 Jan 6. PMID: 29310824.

289: Ray TD, Mekasha S, Liang Y, Lu B, Ram S, Ingalls RR. Species-specific

differences in regulation of macrophage inflammation by the C3a-C3a receptor

axis. Innate Immun. 2018 Jan;24(1):66-78. doi: 10.1177/1753425917747044. Epub

2018 Jan 3. PMID: 29297237; PMCID: PMC6818254.

290: Irmscher S, Döring N, Halder LD, Jo EAH, Kopka I, Dunker C, Jacobsen ID,

Luo S, Slevogt H, Lorkowski S, Beyersdorf N, Zipfel PF, Skerka C. Kallikrein

Cleaves C3 and Activates Complement. J Innate Immun. 2018;10(2):94-105. doi:

10.1159/000484257. Epub 2017 Dec 14. PMID: 29237166; PMCID: PMC6757171.

291: Zhang C, Wang C, Li Y, Miwa T, Liu C, Cui W, Song WC, Du J. Complement C3a

signaling facilitates skeletal muscle regeneration by regulating monocyte

function and trafficking. Nat Commun. 2017 Dec 12;8(1):2078. doi:

10.1038/s41467-017-01526-z. PMID: 29233958; PMCID: PMC5727192.

292: Laule CF, Wing CR, Odean EJ, Wilcox JA, Gilbert JS, Regal JF. Effect of

nicotine on placental ischemia-induced complement activation and hypertension in

the rat. J Immunotoxicol. 2017 Dec;14(1):235-240. doi:

10.1080/1547691X.2017.1394934. PMID: 29185370; PMCID: PMC6298215.

293: Siljan WW, Holter JC, Nymo SH, Husebye E, Ueland T, Aukrust P, Mollnes TE,

Heggelund L. Cytokine responses, microbial aetiology and short-term outcome in

community-acquired pneumonia. Eur J Clin Invest. 2018 Jan;48(1):e12865. doi:

10.1111/eci.12865. Epub 2017 Dec 7. PMID: 29171871; PMCID: PMC5767742.

294: Paredes RM, Reyna S, Vernon P, Tadaki DK, Dallelucca JJ, Sheppard F.

Generation of complement molecular complex C5b-9 (C5b-9) in response to poly-

traumatic hemorrhagic shock and evaluation of C5 cleavage inhibitors in non-

human primates. Int Immunopharmacol. 2018 Jan;54:221-225. doi:

10.1016/j.intimp.2017.10.033. Epub 2017 Nov 20. PMID: 29156357.

295: Sagar A, Dai W, Minot M, LeCover R, Varner JD. Reduced order modeling and

analysis of the human complement system. PLoS One. 2017 Nov 20;12(11):e0187373.

doi: 10.1371/journal.pone.0187373. PMID: 29155837; PMCID: PMC5695804.

296: Tang Y, Li H, Li J, Liu Y, Li Y, Zhou J, Zhou J, Lu X, Zhao W, Hou J, Wang

XY, Chen Z, Zuo D. Macrophage scavenger receptor 1 contributes to pathogenesis

of fulminant hepatitis via neutrophil-mediated complement activation. J Hepatol.

2018 Apr;68(4):733-743. doi: 10.1016/j.jhep.2017.11.010. Epub 2017 Nov 14. PMID:

29154963; PMCID: PMC5951742.

297: Rayes J, Ing M, Delignat S, Peyron I, Gilardin L, Vogel CW, Fritzinger DC,

Frémeaux-Bacchi V, Kaveri SV, Roumenina LT, Lacroix-Desmazes S. Complement C3 is

a novel modulator of the anti-factor VIII immune response. Haematologica. 2018

Feb;103(2):351-360. doi: 10.3324/haematol.2017.165720. Epub 2017 Nov 16. PMID:

29146705; PMCID: PMC5792280.

298: Kwak JW, Laskowski J, Li HY, McSharry MV, Sippel TR, Bullock BL, Johnson

AM, Poczobutt JM, Neuwelt AJ, Malkoski SP, Weiser-Evans MC, Lambris JD, Clambey

ET, Thurman JM, Nemenoff RA. Complement Activation via a C3a Receptor Pathway

Alters CD4<sup>+</sup> T Lymphocytes and Mediates Lung Cancer Progression.

Cancer Res. 2018 Jan 1;78(1):143-156. doi: 10.1158/0008-5472.CAN-17-0240. Epub

2017 Nov 8. PMID: 29118090; PMCID: PMC5810934.

299: Fanelli G, Gonzalez-Cordero A, Gardner PJ, Peng Q, Fernando M, Kloc M,

Farrar CA, Naeem A, Garred P, Ali RR, Sacks SH. Human stem cell-derived retinal

epithelial cells activate complement via collectin 11 in response to stress. Sci

Rep. 2017 Nov 7;7(1):14625. doi: 10.1038/s41598-017-15212-z. PMID: 29116192;

PMCID: PMC5677091.

300: Fernandez-Godino R, Bujakowska KM, Pierce EA. Changes in extracellular

matrix cause RPE cells to make basal deposits and activate the alternative

complement pathway. Hum Mol Genet. 2018 Jan 1;27(1):147-159. doi:

10.1093/hmg/ddx392. PMID: 29095988; PMCID: PMC6251553.

301: Benasutti H, Wang G, Vu VP, Scheinman R, Groman E, Saba L, Simberg D.

Variability of Complement Response toward Preclinical and Clinical Nanocarriers

in the General Population. Bioconjug Chem. 2017 Nov 15;28(11):2747-2755. doi:

10.1021/acs.bioconjchem.7b00496. Epub 2017 Nov 1. PMID: 29090582; PMCID:

PMC6231230.

302: Trendelenburg M, Stallone F, Pershyna K, Eisenhut T, Twerenbold R, Wildi K,

Dubler D, Schirmbeck L, Puelacher C, Rubini Gimenez M, Sabti Z, Osswald L,

Breidthardt T, Müller C. Complement activation products in acute heart failure:

Potential role in pathophysiology, responses to treatment and impacts on long-

term survival. Eur Heart J Acute Cardiovasc Care. 2018 Jun;7(4):348-357. doi:

10.1177/2048872617694674. Epub 2017 Feb 1. PMID: 29064269.

303: Urbanová V, Hajdušek O, Šíma R, Franta Z, Hönig-Mondeková H, Grunclová L,

Bartošová-Sojková P, Jalovecká M, Kopáček P. IrC2/Bf - A yeast and Borrelia

responsive component of the complement system from the hard tick Ixodes ricinus.

Dev Comp Immunol. 2018 Feb;79:86-94. doi: 10.1016/j.dci.2017.10.012. Epub 2017

Oct 20. PMID: 29061482.

304: Bartosova M, Schaefer B, Bermejo JL, Tarantino S, Lasitschka F, Macher-

Goeppinger S, Sinn P, Warady BA, Zaloszyc A, Parapatics K, Májek P, Bennett KL,

Oh J, Aufricht C, Schaefer F, Kratochwill K, Schmitt CP. Complement Activation

in Peritoneal Dialysis-Induced Arteriolopathy. J Am Soc Nephrol. 2018

Jan;29(1):268-282. doi: 10.1681/ASN.2017040436. Epub 2017 Oct 18. PMID:

29046343; PMCID: PMC5748916.

305: Neun BW, Ilinskaya AN, Dobrovolskaia MA. Analysis of Complement Activation

by Nanoparticles. Methods Mol Biol. 2018;1682:149-160. doi:

10.1007/978-1-4939-7352-1_13. PMID: 29039100.

306: Iatropoulos P, Daina E, Curreri M, Piras R, Valoti E, Mele C, Bresin E,

Gamba S, Alberti M, Breno M, Perna A, Bettoni S, Sabadini E, Murer L, Vivarelli

M, Noris M, Remuzzi G; Registry of Membranoproliferative Glomerulonephritis/C3

Glomerulopathy; Nastasi. Cluster Analysis Identifies Distinct Pathogenetic

Patterns in C3 Glomerulopathies/Immune Complex-Mediated Membranoproliferative

GN. J Am Soc Nephrol. 2018 Jan;29(1):283-294. doi: 10.1681/ASN.2017030258. Epub

2017 Oct 13. PMID: 29030465; PMCID: PMC5748907.

307: Merinero HM, García SP, García-Fernández J, Arjona E, Tortajada A,

Rodríguez de Córdoba S. Complete functional characterization of disease-

associated genetic variants in the complement factor H gene. Kidney Int. 2018

Feb;93(2):470-481. doi: 10.1016/j.kint.2017.07.015. Epub 2017 Sep 21. PMID:

28941939.

308: Thurman JM, Laskowski J. Complement factor H-related proteins in IgA

nephropathy-sometimes a gentle nudge does the trick. Kidney Int. 2017

Oct;92(4):790-793. doi: 10.1016/j.kint.2017.05.025. PMID: 28938951.

309: Pawlak A, Rybka J, Dudek B, Krzyżewska E, Rybka W, Kędziora A, Klausa E,

Bugla-Płoskońska G. Salmonella O48 Serum Resistance is Connected with the

Elongation of the Lipopolysaccharide O-Antigen Containing Sialic Acid. Int J Mol

Sci. 2017 Sep 21;18(10):2022. doi: 10.3390/ijms18102022. PMID: 28934165; PMCID:

PMC5666704.

310: Bujko K, Rzeszotek S, Hoehlig K, Yan J, Vater A, Ratajczak MZ. Signaling of

the Complement Cleavage Product Anaphylatoxin C5a Through C5aR (CD88)

Contributes to Pharmacological Hematopoietic Stem Cell Mobilization. Stem Cell

Rev Rep. 2017 Dec;13(6):793-800. doi: 10.1007/s12015-017-9769-6. PMID: 28918528;

PMCID: PMC5730632.

311: Lachmann PJ, Lay E, Seilly DJ. Experimental confirmation of the C3 tickover

hypothesis by studies with an Ab (S77) that inhibits tickover in whole serum.

FASEB J. 2018 Jan;32(1):123-129. doi: 10.1096/fj.201700734. Epub 2017 Aug 29.

PMID: 28855277.

312: Wu L, Uldahl KB, Chen F, Benasutti H, Logvinski D, Vu V, Banda NK, Peng X,

Simberg D, Moghimi SM. Interaction of extremophilic archaeal viruses with human

and mouse complement system and viral biodistribution in mice. Mol Immunol. 2017

Oct;90:273-279. doi: 10.1016/j.molimm.2017.08.009. Epub 2017 Aug 30. PMID:

28846925; PMCID: PMC5661882.

313: Nissilä E, Korpela K, Lokki AI, Paakkanen R, Jokiranta S, de Vos WM, Lokki

ML, Kolho KL, Meri S. C4B gene influences intestinal microbiota through

complement activation in patients with paediatric-onset inflammatory bowel

disease. Clin Exp Immunol. 2017 Dec;190(3):394-405. doi: 10.1111/cei.13040. Epub

2017 Sep 25. PMID: 28832994; PMCID: PMC5680072.

314: Qi J, Wang J, Chen J, Su J, Tang Y, Wu X, Ma X, Chen F, Ruan C, Zheng XL,

Wu D, Han Y. Plasma levels of complement activation fragments C3b and sC5b-9

significantly increased in patients with thrombotic microangiopathy after

allogeneic stem cell transplantation. Ann Hematol. 2017 Nov;96(11):1849-1855.

doi: 10.1007/s00277-017-3092-9. Epub 2017 Aug 11. PMID: 28801815; PMCID:

PMC6225065.

315: Cui J, Wan J, You D, Zou Z, Chen Y, Li Z, Lian Q. Interstitial complement

C3 activation and macrophage infiltration in patients with hypertensive

nephropathy . Clin Nephrol. 2017 Dec;88(12):328-337. doi: 10.5414/CN109154.

PMID: 28793954.

316: Francian A, Mann K, Kullberg M. Complement C3-dependent uptake of targeted

liposomes into human macrophages, B cells, dendritic cells, neutrophils, and

MDSCs. Int J Nanomedicine. 2017 Jul 19;12:5149-5161. doi: 10.2147/IJN.S138787.

PMID: 28790822; PMCID: PMC5529385.

317: Xu R, Lin F, Bao C, Wang FS. Mechanism of C5a-induced immunologic

derangement in sepsis. Cell Mol Immunol. 2017 Sep;14(9):792-793. doi:

10.1038/cmi.2017.68. Epub 2017 Aug 7. PMID: 28782758; PMCID: PMC5596250.

318: DiScipio RG, Schraufstatter IU. Magnetic bead based assays for complement

component C5. J Immunol Methods. 2017 Nov;450:50-57. doi:

10.1016/j.jim.2017.07.010. Epub 2017 Jul 27. PMID: 28757372.

319: Conti G, De Vivo D, Vitale A, Fede C, Santoro D. Dense deposit disease in a

child with febrile sore throat. Saudi J Kidney Dis Transpl. 2017 Jul-

Aug;28(4):925-928. PMID: 28748900.

320: Ding Y, Zhao W, Zhang T, Qiang H, Lu J, Su X, Wen S, Xu F, Zhang M, Zhang

H, Zeng C, Liu Z, Chen H. A haplotype in CFH family genes confers high risk of

rare glomerular nephropathies. Sci Rep. 2017 Jul 20;7(1):6004. doi:

10.1038/s41598-017-05173-8. PMID: 28729648; PMCID: PMC5519609.

321: Togarsimalemath SK, Sethi SK, Duggal R, Le Quintrec M, Jha P, Daniel R,

Gonnet F, Bansal S, Roumenina LT, Fremeaux-Bacchi V, Kher V, Dragon-Durey MA. A

novel CFHR1-CFHR5 hybrid leads to a familial dominant C3 glomerulopathy. Kidney

Int. 2017 Oct;92(4):876-887. doi: 10.1016/j.kint.2017.04.025. Epub 2017 Jul 18.

PMID: 28729035.

322: Rodríguez-Sanz A, Sánchez-Villanueva R, Domínguez-Ortega J, Fiandor AM,

Ruiz MP, Trocoli F, Díaz-Tejeiro R, Cadenillas C, González E, Martínez V, López-

Trascasa M, Quirce S, Selgas R, Bellón T. Mechanisms Involved in

Hypersensitivity Reactions to Polysulfone Hemodialysis Membranes. Artif Organs.

2017 Nov;41(11):E285-E295. doi: 10.1111/aor.12954. Epub 2017 Jul 19. PMID:

28722144.

323: Marinozzi MC, Chauvet S, Le Quintrec M, Mignotet M, Petitprez F, Legendre

C, Cailliez M, Deschenes G, Fischbach M, Karras A, Nobili F, Pietrement C,

Dragon-Durey MA, Fakhouri F, Roumenina LT, Fremeaux-Bacchi V. C5 nephritic

factors drive the biological phenotype of C3 glomerulopathies. Kidney Int. 2017

Nov;92(5):1232-1241. doi: 10.1016/j.kint.2017.04.017. Epub 2017 Jul 14. PMID:

28712854.

324: Suffritti C, Tobaldini E, Schiavon R, Strada S, Maggioni L, Mehta S,

Sandrone G, Toschi-Dias E, Cicardi M, Montano N. Complement and contact system

activation in acute congestive heart failure patients. Clin Exp Immunol. 2017

Nov;190(2):251-257. doi: 10.1111/cei.13011. Epub 2017 Aug 4. PMID: 28707730;

PMCID: PMC5629426.

325: Grosso G, Vikerfors A, Woodhams B, Adam M, Bremme K, Holmström M, Ågren A,

Eelde A, Bruzelius M, Svenungsson E, Antovic A. Thrombin activatable

fibrinolysis inhibitor (TAFI) - A possible link between coagulation and

complement activation in the antiphospholipid syndrome (APS). Thromb Res. 2017

Oct;158:168-173. doi: 10.1016/j.thromres.2017.06.028. Epub 2017 Jun 24. PMID:

28669410.

326: Kurolap A, Eshach-Adiv O, Hershkovitz T, Paperna T, Mory A, Oz-Levi D,

Zohar Y, Mandel H, Chezar J, Azoulay D, Peleg S, Half EE, Yahalom V, Finkel L,

Weissbrod O, Geiger D, Tabib A, Shaoul R, Magen D, Bonstein L, Mevorach D, Baris

HN. Loss of CD55 in Eculizumab-Responsive Protein-Losing Enteropathy. N Engl J

Med. 2017 Jul 6;377(1):87-89. doi: 10.1056/NEJMc1707173. Epub 2017 Jun 28. PMID:

28657861.

327: Bettoni S, Galbusera M, Gastoldi S, Donadelli R, Tentori C, Spartà G,

Bresin E, Mele C, Alberti M, Tortajada A, Yebenes H, Remuzzi G, Noris M.

Interaction between Multimeric von Willebrand Factor and Complement: A Fresh

Look to the Pathophysiology of Microvascular Thrombosis. J Immunol. 2017 Aug

1;199(3):1021-1040. doi: 10.4049/jimmunol.1601121. Epub 2017 Jun 26. PMID:

28652401.

328: Puissant-Lubrano B, Puissochet S, Congy-Jolivet N, Chauveau D, Decramer S,

Garnier A, Huart A, Kamar N, Ribes D, Blancher A. Alternative complement pathway

hemolytic assays reveal incomplete complement blockade in patients treated with

eculizumab. Clin Immunol. 2017 Oct;183:1-7. doi: 10.1016/j.clim.2017.06.007.

Epub 2017 Jun 21. PMID: 28647502.

329: Liu J, Xie J, Zhang X, Tong J, Hao X, Ren H, Wang W, Chen N. Serum C3 and

Renal Outcome in Patients with Primary Focal Segmental Glomerulosclerosis. Sci

Rep. 2017 Jun 22;7(1):4095. doi: 10.1038/s41598-017-03344-1. PMID: 28642464;

PMCID: PMC5481381.

330: Valenzuela NM, Thomas KA, Mulder A, Parry GC, Panicker S, Reed EF.

Complement-Mediated Enhancement of Monocyte Adhesion to Endothelial Cells by HLA

Antibodies, and Blockade by a Specific Inhibitor of the Classical Complement

Cascade, TNT003. Transplantation. 2017 Jul;101(7):1559-1572. doi:

10.1097/TP.0000000000001486. PMID: 28640789; PMCID: PMC5482566.

331: Abdel-Latif M, Abdel-Moneim AA, El-Hefnawy MH, Khalil RG. Comparative and

correlative assessments of cytokine, complement and antibody patterns in

paediatric type 1 diabetes. Clin Exp Immunol. 2017 Oct;190(1):110-121. doi:

10.1111/cei.13001. Epub 2017 Jul 13. PMID: 28640379; PMCID: PMC5588772.

332: Kerr H, Wong E, Makou E, Yang Y, Marchbank K, Kavanagh D, Richards A,

Herbert AP, Barlow PN. Disease-linked mutations in factor H reveal pivotal role

of cofactor activity in self-surface-selective regulation of complement

activation. J Biol Chem. 2017 Aug 11;292(32):13345-13360. doi:

10.1074/jbc.M117.795088. Epub 2017 Jun 21. PMID: 28637873; PMCID: PMC5555194.

333: Sereflican B, Bugdayci G. Components of the alternative complement pathway

in patients with psoriasis. Acta Dermatovenerol Alp Pannonica Adriat. 2017

Jun;26(2):37-40. doi: 10.15570/actaapa.2017.11. PMID: 28632884.

334: Sica M, Rondelli T, Ricci P, De Angioletti M, Risitano AM, Notaro R.

Eculizumab treatment: stochastic occurrence of C3 binding to individual PNH

erythrocytes. J Hematol Oncol. 2017 Jun 19;10(1):126. doi:

10.1186/s13045-017-0496-x. PMID: 28629435; PMCID: PMC5477256.

335: Lorthiois E, Anderson K, Vulpetti A, Rogel O, Cumin F, Ostermann N,

Steinbacher S, Mac Sweeney A, Delgado O, Liao SM, Randl S, Rüdisser S, Dussauge

S, Fettis K, Kieffer L, de Erkenez A, Yang L, Hartwieg C, Argikar UA, La Bonte

LR, Newton R, Kansara V, Flohr S, Hommel U, Jaffee B, Maibaum J. Discovery of

Highly Potent and Selective Small-Molecule Reversible Factor D Inhibitors

Demonstrating Alternative Complement Pathway Inhibition in Vivo. J Med Chem.

2017 Jul 13;60(13):5717-5735. doi: 10.1021/acs.jmedchem.7b00425. Epub 2017 Jun

30. PMID: 28621538.

336: Nilsson PH, Thomas AM, Bergseth G, Gustavsen A, Volokhina EB, van den

Heuvel LP, Barratt-Due A, Mollnes TE. Eculizumab-C5 complexes express a C5a

neoepitope in vivo: Consequences for interpretation of patient complement

analyses. Mol Immunol. 2017 Sep;89:111-114. doi: 10.1016/j.molimm.2017.05.021.

Epub 2017 Jun 10. PMID: 28610663.

337: Lood C, Arve S, Ledbetter J, Elkon KB. TLR7/8 activation in neutrophils

impairs immune complex phagocytosis through shedding of FcgRIIA. J Exp Med. 2017

Jul 3;214(7):2103-2119. doi: 10.1084/jem.20161512. Epub 2017 Jun 12. PMID:

28606989; PMCID: PMC5502427.

338: Natoli R, Fernando N, Jiao H, Racic T, Madigan M, Barnett NL, Chu-Tan JA,

Valter K, Provis J, Rutar M. Retinal Macrophages Synthesize C3 and Activate

Complement in AMD and in Models of Focal Retinal Degeneration. Invest Ophthalmol

Vis Sci. 2017 Jun 1;58(7):2977-2990. doi: 10.1167/iovs.17-21672. PMID: 28605809.

339: Wyatt SK, Witt T, Barbaro NM, Cohen-Gadol AA, Brewster AL. Enhanced

classical complement pathway activation and altered phagocytosis signaling

molecules in human epilepsy. Exp Neurol. 2017 Sep;295:184-193. doi:

10.1016/j.expneurol.2017.06.009. Epub 2017 Jun 7. PMID: 28601603.

340: Ravindran A, Fervenza FC, Smith RJH, Sethi S. C3 glomerulonephritis with a

severe crescentic phenotype. Pediatr Nephrol. 2017 Sep;32(9):1625-1633. doi:

10.1007/s00467-017-3702-8. Epub 2017 Jun 7. PMID: 28593446.

341: Wolf-Grosse S, Rokstad AM, Ali S, Lambris JD, Mollnes TE, Nilsen AM,

Stenvik J. Iron oxide nanoparticles induce cytokine secretion in a complement-

dependent manner in a human whole blood model. Int J Nanomedicine. 2017 May

23;12:3927-3940. doi: 10.2147/IJN.S136453. PMID: 28579778; PMCID: PMC5449102.

342: Csincsi ÁI, Szabó Z, Bánlaki Z, Uzonyi B, Cserhalmi M, Kárpáti É, Tortajada

A, Caesar JJE, Prohászka Z, Jokiranta TS, Lea SM, Rodríguez de Córdoba S, Józsi

M. FHR-1 Binds to C-Reactive Protein and Enhances Rather than Inhibits

Complement Activation. J Immunol. 2017 Jul 1;199(1):292-303. doi:

10.4049/jimmunol.1600483. Epub 2017 May 22. PMID: 28533443.

343: Bahia El Idrissi N, Iyer AM, Ramaglia V, Rosa PS, Soares CT, Baas F, Das

PK. In Situ complement activation and T-cell immunity in leprosy spectrum: An

immunohistological study on leprosy lesional skin. PLoS One. 2017 May

15;12(5):e0177815. doi: 10.1371/journal.pone.0177815. PMID: 28505186; PMCID:

PMC5432188.

344: Denk S, Neher MD, Messerer DAC, Wiegner R, Nilsson B, Rittirsch D, Nilsson-

Ekdahl K, Weckbach S, Ignatius A, Kalbitz M, Gebhard F, Weiss ME, Vogt J,

Radermacher P, Köhl J, Lambris JD, Huber-Lang MS. Complement C5a Functions as a

Master Switch for the pH Balance in Neutrophils Exerting Fundamental

Immunometabolic Effects. J Immunol. 2017 Jun 15;198(12):4846-4854. doi:

10.4049/jimmunol.1700393. Epub 2017 May 10. PMID: 28490576.

345: Wang Z, Guo W, Liu Y, Gong Y, Ding X, Shi K, Thome R, Zhang GX, Shi FD, Yan

Y. Low expression of complement inhibitory protein CD59 contributes to humoral

autoimmunity against astrocytes. Brain Behav Immun. 2017 Oct;65:173-182. doi:

10.1016/j.bbi.2017.04.023. Epub 2017 May 2. PMID: 28476558.

346: Codsi E, Garovic VD, Gonzalez-Suarez ML, Milic N, Borowski KS, Rose CH,

Davies NP, Kashani KB, Lieske JC, White WM. Longitudinal characterization of

renal proximal tubular markers in normotensive and preeclamptic pregnancies. Am

J Physiol Regul Integr Comp Physiol. 2017 May 1;312(5):R773-R778. doi:

10.1152/ajpregu.00509.2016. Epub 2017 Apr 24. PMID: 28438765.

347: Mastellos DC, Reis ES, Ricklin D, Smith RJ, Lambris JD. Complement

C3-Targeted Therapy: Replacing Long-Held Assertions with Evidence-Based

Discovery. Trends Immunol. 2017 Jun;38(6):383-394. doi:

10.1016/j.it.2017.03.003. Epub 2017 Apr 14. PMID: 28416449; PMCID: PMC5447467.

348: Ulusoy C, Çavuş F, Yılmaz V, Tüzün E. Immunization with Recombinantly

Expressed LRP4 Induces Experimental Autoimmune Myasthenia Gravis in C57BL/6

Mice. Immunol Invest. 2017 Jul;46(5):490-499. doi:

10.1080/08820139.2017.1299754. Epub 2017 Apr 4. PMID: 28375749.

349: Rosain J, Hong E, Fieschi C, Martins PV, El Sissy C, Deghmane AE, Ouachée

M, Thomas C, Launay D, de Pontual L, Suarez F, Moshous D, Picard C, Taha MK,

Frémeaux-Bacchi V. Strains Responsible for Invasive Meningococcal Disease in

Patients With Terminal Complement Pathway Deficiencies. J Infect Dis. 2017 Apr

15;215(8):1331-1338. doi: 10.1093/infdis/jix143. PMID: 28368462.

350: Seignez A, Joly AL, Chaumonnot K, Hazoumé A, Sanka M, Marcion G, Boudesco

C, Hammann A, Seigneuric R, Jégo G, Ducoroy P, Delarue P, Senet P, Castilla-

Llorente C, Solary E, Durey MA, Rubio MT, Hermine O, Kohli E, Garrido C. Serum

Gp96 is a chaperone of complement-C3 during graft-versus-host disease. JCI

Insight. 2017 Mar 23;2(6):e90531. doi: 10.1172/jci.insight.90531. PMID:

28352659; PMCID: PMC5358489.

351: Garcia BL, Skaff DA, Chatterjee A, Hanning A, Walker JK, Wyckoff GJ,

Geisbrecht BV. Identification of C3b-Binding Small-Molecule Complement

Inhibitors Using Cheminformatics. J Immunol. 2017 May 1;198(9):3705-3718. doi:

10.4049/jimmunol.1601932. Epub 2017 Mar 15. PMID: 28298523; PMCID: PMC5417336.

352: Zhang Y, Yan X, Zhao T, Xu Q, Peng Q, Hu R, Quan S, Zhou Y, Xing G.

Targeting C3a/C5a receptors inhibits human mesangial cell proliferation and

alleviates immunoglobulin A nephropathy in mice. Clin Exp Immunol. 2017

Jul;189(1):60-70. doi: 10.1111/cei.12961. Epub 2017 Apr 10. PMID: 28295247;

PMCID: PMC5461107.

353: Quach QH, Kah JC. Non-specific adsorption of complement proteins affects

complement activation pathways of gold nanomaterials. Nanotoxicology. 2017

Apr;11(3):382-394. doi: 10.1080/17435390.2017.1306131. Epub 2017 Apr 3. PMID:

28287003.

354: Qiao P, Dang E, Cao T, Fang H, Zhang J, Qiao H, Wang G. Dysregulation of

mCD46 and sCD46 contribute to the pathogenesis of bullous pemphigoid. Sci Rep.

2017 Mar 10;7(1):145. doi: 10.1038/s41598-017-00235-3. PMID: 28273946; PMCID:

PMC5428046.

355: Song D, Guo WY, Wang FM, Li YZ, Song Y, Yu F, Zhao MH. Complement

Alternative Pathway׳s Activation in Patients With Lupus Nephritis. Am J Med Sci.

2017 Mar;353(3):247-257. doi: 10.1016/j.amjms.2017.01.005. Epub 2017 Jan 13.

PMID: 28262211.

356: Lebreton C, Bacchetta J, Dijoud F, Bessenay L, Fremeaux-Bacchi V, Sellier-

Leclerc AL. C3 glomerulopathy and eculizumab: a report on four paediatric cases.

Pediatr Nephrol. 2017 Jun;32(6):1023-1028. doi: 10.1007/s00467-017-3619-2. Epub

2017 Feb 24. PMID: 28236143.

357: Pandey MK, Burrow TA, Rani R, Martin LJ, Witte D, Setchell KD, Mckay MA,

Magnusen AF, Zhang W, Liou B, Köhl J, Grabowski GA. Complement drives

glucosylceramide accumulation and tissue inflammation in Gaucher disease.

Nature. 2017 Mar 2;543(7643):108-112. doi: 10.1038/nature21368. Epub 2017 Feb

22. PMID: 28225753.

358: Subramaniam S, Jurk K, Hobohm L, Jäckel S, Saffarzadeh M, Schwierczek K,

Wenzel P, Langer F, Reinhardt C, Ruf W. Distinct contributions of complement

factors to platelet activation and fibrin formation in venous thrombus

development. Blood. 2017 Apr 20;129(16):2291-2302. doi:

10.1182/blood-2016-11-749879. Epub 2017 Feb 21. PMID: 28223279; PMCID:

PMC5399485.

359: Guo DD, Hu B, Tang HY, Sun YY, Liu B, Tian QM, Bi HS. Proteomic Profiling

Analysis Reveals a Link between Experimental Autoimmune Uveitis and Complement

Activation in Rats. Scand J Immunol. 2017 May;85(5):331-342. doi:

10.1111/sji.12539. PMID: 28199731.

360: Claisse G, Absi L, Cognasse F, Alamartine E, Mariat C, Maillard N.

Relationship between Mean Fluorescence Intensity and C1q/C3d-fixing capacities

of anti-HLA antibodies. Hum Immunol. 2017 Apr;78(4):336-341. doi:

10.1016/j.humimm.2017.02.003. Epub 2017 Feb 9. PMID: 28189573.

361: Yaseen S, Demopulos G, Dudler T, Yabuki M, Wood CL, Cummings WJ, Tjoelker

LW, Fujita T, Sacks S, Garred P, Andrew P, Sim RB, Lachmann PJ, Wallis R, Lynch

N, Schwaeble WJ. Lectin pathway effector enzyme mannan-binding lectin-associated

serine protease-2 can activate native complement C3 in absence of C4 and/or C2.

FASEB J. 2017 May;31(5):2210-2219. doi: 10.1096/fj.201601306R. Epub 2017 Feb 10.

PMID: 28188176.

362: Timmermans SAMEG, Abdul-Hamid MA, Vanderlocht J, Damoiseaux JGMC,

Reutelingsperger CP, van Paassen P; Limburg Renal Registry. Patients with

hypertension-associated thrombotic microangiopathy may present with complement

abnormalities. Kidney Int. 2017 Jun;91(6):1420-1425. doi:

10.1016/j.kint.2016.12.009. Epub 2017 Feb 7. PMID: 28187980.

363: van der Maten E, de Jonge MI, de Groot R, van der Flier M, Langereis JD. A

versatile assay to determine bacterial and host factors contributing to

opsonophagocytotic killing in hirudin-anticoagulated whole blood. Sci Rep. 2017

Feb 8;7:42137. doi: 10.1038/srep42137. PMID: 28176849; PMCID: PMC5296863.

364: Rasmussen KJ, Skjoedt MO, Vitved L, Skjoedt K, Palarasah Y. A novel

antihuman C3d monoclonal antibody with specificity to the C3d complement split

product. J Immunol Methods. 2017 May;444:51-55. doi: 10.1016/j.jim.2017.02.002.

Epub 2017 Feb 4. PMID: 28174050.

365: Dixon KO, O'Flynn J, Klar-Mohamad N, Daha MR, van Kooten C. Properdin and

factor H production by human dendritic cells modulates their T-cell stimulatory

capacity and is regulated by IFN-γ. Eur J Immunol. 2017 Mar;47(3):470-480. doi:

10.1002/eji.201646703. PMID: 28105653; PMCID: PMC5363362.

366: Subías Hidalgo M, Yébenes H, Rodríguez-Gallego C, Martín-Ambrosio A,

Domínguez M, Tortajada A, Rodríguez de Córdoba S, Llorca O. Functional and

structural characterization of four mouse monoclonal antibodies to complement C3

with potential therapeutic and diagnostic applications. Eur J Immunol. 2017

Mar;47(3):504-515. doi: 10.1002/eji.201646758. Epub 2017 Feb 6. PMID: 28083930.

367: Holt BA, Bellavia MC, Potter D, White D, Stowell SR, Sulchek T. Fc

microparticles can modulate the physical extent and magnitude of complement

activity. Biomater Sci. 2017 Feb 28;5(3):463-474. doi: 10.1039/c6bm00608f. PMID:

28067347; PMCID: PMC5330945.

368: Harder MJ, Kuhn N, Schrezenmeier H, Höchsmann B, von Zabern I, Weinstock C,

Simmet T, Ricklin D, Lambris JD, Skerra A, Anliker M, Schmidt CQ. Incomplete

inhibition by eculizumab: mechanistic evidence for residual C5 activity during

strong complement activation. Blood. 2017 Feb 23;129(8):970-980. doi:

10.1182/blood-2016-08-732800. Epub 2016 Dec 27. PMID: 28028023; PMCID:

PMC5324716.

369: Chen F, Wang G, Griffin JI, Brenneman B, Banda NK, Holers VM, Backos DS, Wu

L, Moghimi SM, Simberg D. Complement proteins bind to nanoparticle protein

corona and undergo dynamic exchange in vivo. Nat Nanotechnol. 2017

May;12(4):387-393. doi: 10.1038/nnano.2016.269. Epub 2016 Dec 19. PMID:

27992410; PMCID: PMC5617637.

370: Goodship TH, Cook HT, Fakhouri F, Fervenza FC, Frémeaux-Bacchi V, Kavanagh

D, Nester CM, Noris M, Pickering MC, Rodríguez de Córdoba S, Roumenina LT, Sethi

S, Smith RJ; Conference Participants. Atypical hemolytic uremic syndrome and C3

glomerulopathy: conclusions from a "Kidney Disease: Improving Global Outcomes"

(KDIGO) Controversies Conference. Kidney Int. 2017 Mar;91(3):539-551. doi:

10.1016/j.kint.2016.10.005. Epub 2016 Dec 16. PMID: 27989322.

371: Giacomassi C, Buang N, Ling GS, Crawford G, Cook HT, Scott D, Dazzi F,

Strid J, Botto M. Complement C3 Exacerbates Imiquimod-Induced Skin Inflammation

and Psoriasiform Dermatitis. J Invest Dermatol. 2017 Mar;137(3):760-763. doi:

10.1016/j.jid.2016.11.011. Epub 2016 Nov 19. PMID: 27876407; PMCID: PMC5319416.

372: Verghese PS, Reed RC, Lihong B, Matas AJ, Kim Y. The clinical implications

of the unique glomerular complement deposition pattern in transplant

glomerulopathy. J Nephrol. 2018 Feb;31(1):157-164. doi:

10.1007/s40620-016-0365-7. Epub 2016 Nov 15. PMID: 27848227.

373: Yuan X, Gavriilaki E, Thanassi JA, Yang G, Baines AC, Podos SD, Huang Y,

Huang M, Brodsky RA. Small-molecule factor D inhibitors selectively block the

alternative pathway of complement in paroxysmal nocturnal hemoglobinuria and

atypical hemolytic uremic syndrome. Haematologica. 2017 Mar;102(3):466-475. doi:

10.3324/haematol.2016.153312. Epub 2016 Nov 3. PMID: 27810992; PMCID:

PMC5394948.

374: Hilhorst M, van Paassen P, van Rie H, Bijnens N, Heerings-Rewinkel P, van

Breda Vriesman P, Cohen Tervaert JW; Limburg Renal Registry. Complement in ANCA-

associated glomerulonephritis. Nephrol Dial Transplant. 2017 Aug

1;32(8):1302-1313. doi: 10.1093/ndt/gfv288. PMID: 26275893.

375: Wehling C, Amon O, Bommer M, Hoppe B, Kentouche K, Schalk G, Weimer R,

Wiesener M, Hohenstein B, TÃ¶nshoff B, BÃ¼scher R, Fehrenbach H, GÃ¶k Ã–N,

Kirschfink M. Monitoring of complement activation biomarkers and eculizumab in

complement-mediated renal disorders. Clin Exp Immunol. 2017 Feb;187(2):304-315.

doi: 10.1111/cei.12890. Epub 2016 Nov 25. PMID: 27784126; PMCID: PMC5217898.

376: SubÃ­as Hidalgo M, Martin Merinero H, LÃ³pez A, Anter J, GarcÃ­a SP, AtaÃºlfo

Gonzalez-FernÃ¡ndez F, ForÃ©s R, Lopez-Trascasa M, Villegas A, Ojeda E, RodrÃ­guez

de CÃ³rdoba S. Extravascular hemolysis and complement consumption in Paroxysmal

Nocturnal Hemoglobinuria patients undergoing eculizumab treatment.

Immunobiology. 2017 Feb;222(2):363-371. doi: 10.1016/j.imbio.2016.09.002. Epub

2016 Sep 13. PMID: 27644115.

**Supplementary table 1.** Overview of studies that assessed terminal pathway components *in-vivo* in the past 5 years (2017-2022).

| Author | PMID | Aim of the study | Type of study | Groups analyzed | TP markers assessed | Sample matrix | Correct use of serum/plasma | Technique used for analysis |
| --- | --- | --- | --- | --- | --- | --- | --- | --- |
| Huber S | 34960645 | Investigate systemic and local complement activation and NETosis in COVID-19 patients that underwent mechanical ventilation | Disease monitoring | COVID19 vs healthy controls | C5a, TCC | serum | No | ELISA |
| Witczak BJ | 34759922 | Investigate whether early posttransplant complement system activation is associated with decreased long-term kidney graft and overall survival in kidney transplant recipients | Treatment | Kidney transplant recipients | TCC | EDTA-plasma | Yes | ELISA |
| Liu M | 34671343 | Investigate complement proteins as early-pregnancy predictors and potential diagnostic marker of preeclampsia | Disease monitoring | Healthy and preeclampsia pregnant women before delivery | C3a, C5a | EDTA-plasma | Yes | ELISA |
| Cugno M | 34592707 | Evaluate the involvement of the complement system in a patient with vaccine-induced thrombotic thrombocytopenia (VITT) that occurred after ChAdOx1 nCov-19 (AstraZeneca)vaccination | Treatment | Case report COVID19 patient | C3, TCC | Plasma not further specified | ? | Mass spectrometry |
| Kim DM | 34587481 | Investigate mechanisms of fatal viral pneumonia in COVID-19 | Disease monitoring | COVID19 patients | C3a, C5a | Plasma, BALF, Sputum not further specified | ? | ELISA |
| Pache F | 34464830 | Investigate complement C3 and C4 plasma concentrations in patients with clinically stable AQP4-IgG+ NMOSD (neuromyelitis optica spectrum disorder), MOGAD (myelin oligodendrocyte glycoprotein antibody-associated disease), early multiple sclerosis and in healthy controls. | Disease monitoring | MOGAD, MS and HC | C3 | EDTA-plasma | Yes | Immunoturbidimetry assay |
| Michels MAHM | 34456924 | Investigated the CP convertase activity in patients with C3G and IC-MPGN (immune complex-mediated membranoproliferative glomerulonephritis) | Disease monitoring | C3G, IC-MPGN patients, HC | C5, TCC | EDTA-plasma | Yes | ELISA |
| Nilsson PH | 34380648 | Characterize novel human whole blood ex vivo model based on the GPRP peptide for anticoagulation and examine its utility to assess the effect of thrombin on complement activation in human whole blood | Techical study | GPRP- and lepirudin-anticoagulated plasma | C3, C5, C5a, TCC | GPRP- and lepirudin-anticoagulated plasma | Yes | ELISA |
| Prens LM | 34252397 | Evaluate systemic complement activation in the plasma of patients with hidradenitis suppurativa (HS) | Disease monitoring | HS patients and HC | C3, C5a, TCC | EDTA-plasma | Yes | ELISA, Radial immunodiffusion technique |
| Pollack S | 34248927 | Investigate the molecularcbasis of early-onset aHUS, associated with an unusual finding of a novel homozygous activating deletion in C3 | Disease monitoring | aHUS case report *vs* HC | C3, TCC | Plasma not further specified | ? | Not specified |
| Chiu YL | 34177889 | Investigate alternative pathway activation and disease activity in IgA nephropathy (IgAN) | Disease monitoring | IgAN and HC | C5a | EDTA-plasma | Yes | ELISA |
| Dhooge PPA | 34170959 | Assess systemic complement activation in Stargardt disease (STGD1) patients and its association with disease severity | Disease monitoring | STGD1 patients and controls | C3 | EDTA-plasma | Yes | ELISA |
| Barragán AF | 33852227 | Description case report C3 Glomerulonephritis patient | Disease monitoring | Case report C3G | C3 | Not specified | ? | Not specified |
| Sinkovits G | 33841446 | Investigate whether complement activation is related to the severity and mortality of COVID-19 | Disease monitoring | COVID19 severity groups | C3a, TCC | EDTA-plasma | Yes | ELISA |
| Rognes IN | 33832430 | Describe the kinetics of complement activation in trauma patients from admission to 10 days after injury, and the association with trauma characteristics and outcome | Disease monitoring | Trauma patients | TCC | EDTA-plasma | Yes | ELISA |
| Ruffatti A | 33649771 | Investigate TCC and C5a anaphylatoxin during the quiescent phases of catastrophic antiphospholipid syndrome (CAPS) | Disease monitoring | CAPS patients and controls | C5a, TCC | Plasma not futher specified | ? | ELISA |
| Milosevits G | 33549818 | Study the role of infusion rate and complement activation in infusion reactions (IRs) in pediatric patients treated with Abelcet | Treatment | Pediatric patients treated with Abelcet | C3a | EDTA-plasma | Yes | ELISA |
| Hubens WHG | 33493474 | Investigate changes in complement protein concentrations patients with progressive primary open angle glaucoma (POAG) | Disease monitoring | POAG patients and controls | C3, C3a | Serum | No | ELISA |
| Valenti L | 33453462 | Examine whether chromosome 3p21.31 and the ABO variants are linked to the activation of the complement cascade in COVID-19 patients. | Disease monitoring | COVID19 severity groups | C5a, TCC | Plasma not furher specified | ? | ELISA |
| Ozen A | 33398182 | Investigated Eculizumab treatment in Complement hyperactivation, angiopathic thrombosis, and protein-losing enteropathy (CHAPLE) disease | Treatment | CHAPLE patients treated with Eculizumab | C3, C4, C5,CH5, C3a,C3b,C4a,C4b, C5a, TCC | Serum C3 C4, Plasma C3a, C4a, and C5a, TCC | No | Serum C3 and C4: turbidimetric method. C3a, C4a, C5a: flow cytometry based cytometric bead array. TCC: ELISA |
| Willrich MAV | 33321132 | Investigate if clinical lab tests traditionally used to monitor complement blockade for eculizumab are appropriate for monitoring complement blockade caused by ravulizumab. | Techical study | Residual waste serum samples unspecified | C5 | Serum | No | Nephelometric assay |
| De Nooijer | 33038254 | Assess the role of the complement system in plasma of patients with COVID-19 and its relation to the host immune response, disease severity, clinical course, and outcomes | Disease monitoring | COVID19 and Sepsis patient vs HCs | C3a, C3c, TCC | EDTA-plasma | Yes | ELISA |
| Mastellos DC | 32961333 | Compare the efficacy of the C5-targeting monoclonal antibody eculizumab with that of the compstatin-based C3-targeted drug candidate AMY-101 in small independent cohorts of severe COVID-19 patients | Treatment | COVID19 patients treated with Eculizumab vs AMY-101 | C3, C4, FB, C3a, TCC, AP50 | Plasma not furher specified: C3, C4, FB, C3dg. C3a, TCC measured in EDTA-plasma | Yes | Nephelometry and ELISA |
| Troldborg A | 32941885 | Characterization of mAb for C3dg and development of time-resolved immunoassay with specificity for C3dg that can be used to directly evaluate ongoing complement activation | Techical study | The SLE patients and controls | C3dg | EDTA-plasma | Yes | ELISA |
| Stepniewska J | 32776910 | Determine whether the type of renal replacement therapy has an effect on activation of the complement system | Treatment | patients: 30 on haemodialysis (HD), 21 on peritoneal dialysis (PD) and 28 patients with CKD stages 4 – 5 KDIGO guidelines | C3a, C5a and TCC | Plasma not further specified | ? | ELISA |
| Tachi A | 32581413 | Investigation of serum biological profiles in neonates with Congenital diaphragmatic hernia (CDH) | Disease monitoring | CDH cases and matched HCs | C5 | Umbilical cord serum samples | No | Mass spectrometry |
| Parry J | 32451568 | Investigate the levels of TCC in serum samples of traumatic brain injury (TBI) patients | Disease monitoring | TBI patients | TCC | Serum | No | ELISA |
| Halkjær L | 32431705 | Establishing a sensitive and robust assay for estimation of systemic complement activation at complement component C3 level in mouse and human plasma samples | Techical study | SLE patients | C3dg | CHES for assay validation | Yes | ELISA |
| Arriens C | 32371480 | To evaluate the association between lupus severity and cell-bound complement activation products (CB-CAPs) or low complement proteins C3 and C4 | Disease monitoring | SLE patients | C3, C4 | Serum | No | Flow cytometry and turbidimetry |
| Mastaglio S | 32360516 | Report the clinical course of a patient with severe ARDS due to COVID-19 pneumonia treated with the compstatin-based complement C3 inhibitor AMY-101 | Treatment | COVID19 patient | C3 | Plasma not furher specified | ? | Not specified |
| Martínez-López D | 32327104 | Analyze the temporal and topologically resolved protein changes takingplacein human aortas with early atherosclerosis tofind new potential diagnostic and/or therapeutic targets | Disease monitoring | Early atherosclerosis patients | C5 | Plasma not further specified | ? | ELISA |
| Denzinger M | 32281172 | Activation of the complement cascade at the interface of wound dressings | Disease monitoring | HCs | TCC | EDTA-plasma | Yes | Not specified |
| Heesterbeek TJ | 32176267 | Study the levels of complement activation in different disease stages of AMD and the influence of genetic polymorphisms in complement genes | Disease monitoring | AMD patients and HC | C3, C3d | Serum | No | Radial immunodiffusion Rocket electrophoresis |
| Grinde D | 32152940 | Evaluate the complement system in relation to clinical and immunological parameters in DiGeorge syndrome patients | Disease monitoring | DiGeorge syndrome patients | TCC | EDTA-plasma | Yes | ELISA |
| Shahulhameed S | 32117292 | Investigation of the role of the complement system in vitreous humor and serum samples from proliferative diabetic retinopathy (PDR) patients and controls | Disease monitoring | PDR patient and HC | C3, iC3b, C3 alpha chain, C3 beta chain, C3c alpha chain | Serum | No | WB; quantifying mean band intensity |
| Kristensen MK | 32082310 | Assess whether different complement factors and complement activation products were associated with poor outcome in patients with necrotizing soft-tissue infection (NSTI) | Disease monitoring | NSTI patients vs HC | C3, C4c, C3bc, TCC | EDTA-plasma | Yes | ELISA |
| Carrara C | 32050203 | Assess Eculizumab treatment in C3G patients | Treatment | C3G patients before and after Eculizumab treatment | TCC | Plasma not further specified | ? | Not specified |
| Chauvet S | 32034108 | Investigate mechanisms of complement activation in children with acute postinfectious GN and low C3 level at onset | Disease monitoring | children with acute postinfectious GN vs childern with C3G and persistent hypocomplementemia | C3, C4, TCC | EDTA-plasma | Yes | ELISA |
| Gulleroglu K | 32008503 | Evaluate complement dysregulation and disease recurrence after renal transplant | Treatment | Case report 1 aHUS and 1 C3G patient | C3 | Serum | No | Not specified |
| Abe T | 31917735 | Investigate complement activation in relation to the clinical characteristics of sepsis, including disseminated intravascular coagulation (DIC), interventions, and prognosis | Disease monitoring | Sepsis patients with and without DIC. | TCC | EDTA-plasma | Yes | ELISA |
| Sartain S | 31774252 | Investigate AP activation in Transplant-associated thrombotic microangiopathy (TA-TMA) after hematopoietic stem cell transplantation (HSCT) | Treatment | HSCT patients with TA-TMA and without TA-TMA | C3a, C5a, TCC | EDTA-plasma | Yes | ELISA |
| Tjernberg AR | 31691001 | explore whether the complement response to Streptococcus pneumoniae differed according to celiac disease (CD) status, and could serve as an explanation for the excess risk of invasive pneumococcal disease (IPD) in CD | Disease monitoring | CD vs HC | C3a, TCC | EDTA-plasma | Yes | ELISA |
| Faria B | 31497011 | Investigate the effect of intravenous iron on complement activation in-vivo, and whether this subsequently induces inflammation and/or oxidative stress | Treatment | non-dialysis vs dialysis patients | TCC | EDTA-plasma | Yes | ELISA |
| Scambi C | 31479579 | Investigate complement activation in women with antiphospholipid syndrome (APS) | Disease monitoring | APS non‐pregnant patients and pregnant APS women | C5a, TCC | Citrate-plasma | Yes | ELISA |
| Ramsey-Goldman R | 31469249 | To evaluate the frequency of cell-bound complement activation products (CB-CAPs) as a marker of complement activation in patients with suspected systemic lupus erythematosus (SLE) | Disease monitoring | SLE patients, Sjögren’s syndrome (SS) patients, patients with other rheumatic conditions | C3, C4 | Serum | No | Immunoturbidimetry assay |
| Schein TN | 31461782 | Examine plasma TCC levels in HIV patients with poor immune reconstitution | Disease monitoring | HIV-infected patients and HC | TCC | EDTA-plasma | Yes | ELISA |
| Zhang MF | 31399080 | Examine complement activation products in circulation and urine of patients with primary membranous nephropathy (pMN) | Disease monitoring | Patients with biopsy-proven pMN vs HCs | C3a, C5a, TCC | EDTA-plasma | Yes | ELISA |
| Vercauteren KOA | 31379459 | assess preanalytical stability of widely used tests to screen the complement system. | Techical study | HCs | C3d, C3c | EDTA-plasma | Yes | ELISA, nephelometric (C3d) and immunofixation after PEG precipitation |
| Hokstad I | 31335881 | Examine complement activation in spondylarthropathies (SpA), and its relationship to antirheumatic treatment, inflammatory and cardiovascular markers. | Treatment | SpA patients starting anti-TNF therapy with/without MTX | TCC | Not specified | ? | ELISA |
| Gavriilaki E | 31266080 | evaluation of markers for complement activation, neutrophil extracellular trap (NET) release, endothelial damage, and activation of coagulation cascade in the circulation of patients with transplant-associated thrombotic microangiopathy (TA-TMA) | Disease monitoring | Patients diagnosed with TA-TMA, patients diagnosed with acute and/or chronic GVHD, and control HCT recipients without GVHD or TA-TMA | TCC | EDTA-plasma | Yes | ELISA |
| García L | 31222573 | Analyze the association of hypocomplementemia with the clinical manifestations, laboratory data, renal histology, progress to renal insufficiency, and mortality of patients with AAV | Disease monitoring | AAV patients | C3 | Not specified | ? | Not specified |
| Lynch AM | 31203676 | Examine the role of systemic activation of the complement system in patients with advanced age-related macular degeneration, geographic atrophy, and neovascular age-related macular degeneration | Disease monitoring | AMD patients *vs* cataract controls | C3a, TCC | EDTA-plasma | Yes | ELISA |
| Elvington M | 31019515 | Development and Optimization of an ELISA to Quantitate C3(H2O) as a Marker of Human Disease | Techical study | Inflammatory-driven diseases vs HCs | C3(H20) | Serum and plasma | Yes | ELISA |
| Wang Y | 30989586 | Investigate the relationship between complement activation and vascular calcification in dialyzed patients | Disease monitoring | Hemodialysis patients vs HCs | C3a, C3c, C5a, and C5b-9. | Plasma not further specified | ? | ELISA |
| Høiland II | 30920726 | Investigate the association between plasma levels of TCC and future risk of incident venous thromboembolism (VTE) | Disease monitoring | VTE patiens vs HC | TCC | EDTA-plasma | Yes | ELISA |
| Mansur S | 30889724 | Study the polyethersulphone (PES) membrane blended with polyurethane (PU) for blood purification applications | Techical study | Plasma with and without PU | C3a | Citrate-plasma | Yes | ELISA |
| Raymond W | 30687766 | Monitor complement levels in patients with systemic lupus erythematosus | Disease monitoring | SLE patients | C3 | Not specified | ? | Not specified |
| Michelis R | 30601845 | Study the structure of circulating C components and evaluate the importance of C5 structural integrity for C activity in CLL patients. | Disease monitoring | CCL patients | C5a, TCC | Plasma not further specified | ? | ELISA |
| Burwick RM | 30399106 | Evaluate whether C5b-9 concentrations in blood and urine are increased in preeclampsia | Disease monitoring | Women with preeclampsia with severe features *vs* control group women who were healthy, had chronic hypertension, gestational hypertension, or preeclampsia without severe features. | TCC | EDTA-plasma | Yes | ELISA |
| Rodríguez E | 30380547 | Investigate the role of complement system in the pathogenesis of human AKI. | Disease monitoring | Patients with hospital-acquired AKI and controls | TCC | EDTA-plasma | Yes | ELISA |
| Chauvet S | 30333829 | Investigate the mechanisms of complement dysregulation in patients with C3G with monoclonal immunoglobulin (MIg-C3G) | Disease monitoring | MIg-C3G patients | C3, TCC | EDTA-plasma | Yes | C3 nephelometry, TCC ELISA |
| Karnisova L | 30251107 | analyze C3 as a predictor of clinical courses in patients with diarrhea-associated hemolytic uremic syndrome | Disease monitoring | Diarrhea-associated hemolytic uremic syndrome patients | C3 | Not specified | ? | Not specified |
| Guo WY | 30219152 | explored the combined genetic effects of coding and noncoding variants in CFH on complement activation in immunoglobulin A nephropathy (IgAN) | Disease monitoring | IgAN patients and HCs | C3 | Plasma not further specified | ? | Not specified |
| Farrokhi Yekta R | 30058426 | Identification of markers for papillary thyroid carcinoma | Disease monitoring | PTC patient vs HCs | C3 | Serum | No | Mass spectrometry & ELISA |
| Budkowska M | 29911288 | Investigate influence of circadian rhythm on complement activation | Disease monitoring | HCs | C3a, C5a, TCC | Serum | No | ELISA |
| Bavia L | 29908956 | investigate products of complement activation, C3d and soluble C5b9 (sC5b9), as potential biomarkers for myocardial injury and inflammation | Disease monitoring | Patients with acute myocardial infarction (AMI) and control patients undergoing cardiac catheterization (CC) with normal coronary arteries | C3d, TCC | EDTA-plasma | Yes | rocket immunoelectrophoresis and ELISA |
| Békássy ZD | 29884545 | Demonstrated that renin, a kidneyspecific enzyme, cleaves C3 into C3b and C3a, in a manner identical to the C3 convertase | Disease monitoring | Dense deposit disease (DDD) patients and HC | C3, C3a, C5, and C5a | Serum | No | C3 nephelometry, C3a, C5a ELISA |
| Ravindran A | 29729982 | Investigate whether monoclonal immunoglobulins inhibit the AP of complement in C3G patients | Disease monitoring | C3G patients | C3, TCC | Serum | No | TCC ELISA, C3 not specified |
| Espinosa-Figueroa JL | 29477994 | Report of a patient with two episodes of acute renal failure, bothtimes diagnosed by renal biopsy of acute endocapillary glomerulonephritis, with slow recovery after two episodes of low-serum complement C3, haematuria and proteinuria | Disease monitoring | C3G patient | C3 | Serum | No | Not specified |
| Kanni T | 29405257 | Explore complement activation in hidradenitis suppurativa (HS) | Disease monitoring | HS patients vs HCs | C5a, TCC | Heparin-plasma | Yes | ELISA |
| Lorés-Motta L | 29398083 | Study the levels of complement activation in different disease stages of AMD and the influence of genetic polymorphisms in CFH and CFHR3 | Disease monitoring | AMD patients and HCs | C3, C3d | Serum | No | Radial immunodiffusion using monospecific polyclonal rabbit antisera, and C3d was measured by rocket electrophoresis |
| Kim MY | 29371202 | Investigated whether activation of complement early in pregnancy predicts (adverse pregnancy outcomes) APOs in women with systemic lupus erythematosus (SLE) and/or antiphospholipid antibodies (aPL) | Disease monitoring | Women with systemic lupus erythematosus (SLE) | C3a, iC3b, C5a, TCC | Not specified | ? | ELISA |
| Ren W | 29367209 | Investigate the role of the complement system in (thoracic aortic dissection) TAD | Disease monitoring | TAD patients and age-matched non-TAD individuals | C3a, C4a, and C5a | Plasma not further specified | ? | Bead-assays, ELISA |
| Zhu X | 29350259 | e investigated the complement-regulatory role of β2-GPI in anti-GPIIb/IIIa-mediated f immune thrombocytopenia (ITP) | Disease monitoring | ITP patients vs control patients with non-immune thrombocytopenia | C3b | Serum | No | ELISA |
| Siljan WW | 29171871 | Examine the association of multiple cytokines and the terminal complement complex (TCC) with microbial aetiology, disease severity and shortterm outcome in (community-acquired pneumonia) CAP. | Disease monitoring | CAP patients severity groups | TCC | EDTA-plasma | Yes | ELISA |
| Trendelenburg M | 29064269 | Explore the role of complement activation the in pathophysiology, responses to treatment and impacts on long-term survival in acute heart failure (AHF) | Disease monitoring | Patients with AHF vs HCs | C3a, TCC | EDTA-plasma | Yes | ELISA |
| Latropoulos P | 29030465 | Explore the presence of distinct disease entities characterized by specific pathophysiologic mechanisms | Disease monitoring | C3 glomerulopathy (C3G) and immune complex–mediated membranoproliferative GN (IC-MPGN) patients | C3, TCC | Serum (C3) and plasma not further specified (TCC) | No | Nephelometry (C3) and ELISA (TCC) |
| Qi J | 28801815 | Investigate complement activation in in patients with thrombotic microangiopathy after allogeneic stem cell transplantation (Transplantation-associated thrombotic microangiopathy; TA-TMA) | Treatment | TA-TMA patients and patients without TA-TMA) | C3b, TCC | EDTA-plasma | Yes | ELISA |
| Togarsimalemath SK | 28729035 | Describe a novel genetic rearrangement generated from a heterozygous deletion spanning 146 Kbp involving multiple CFHR genes leading to a CFHR1-R5 hybrid protein | Disease monitoring | C3G patients | C3, TCC | EDTA-plasma | Yes | ELISA |
| Rodríguez-Sanz A | 28722144 | Investigate hypersensitivity reactions to polysulfone (PS)hemodialysis (HD) membrane | Techical study | Patients with systemic reactions to PS-based membranes | C3 | Serum | No | Not specified |
| Suffritti C | 28707730 | evaluate contact and complement systems activation in patients hospitalized for an acute episode of congestive heart failure (CHF) | Disease monitoring | CHF patients | C3, TCC | EDTA-plasma | Yes | radial immunodiffusion + ELISA |
| Grosso G | 28669410 | Investigate the levels of TAFI and TAFIa in relation to complement activation, fibrin clot permeability and fibrinolytic function in clinical and immunological subsets of antiphospholipid syndrome (APS) patients | Disease monitoring | APS patients vs HC | C5a | Citrate-plasma | Yes | ELISA |
| Puissant-Lubrano B | 28647502 | Monitoring complement inhibition in eculizumab-treated patients suffering from HUS or transplant rejection | Treatment | STEC-HUS patient before and after Eculizumab treatment | C5a, TCC | Serum | No | ELISA |
| Liu J | 28642464 | Assess the relationship between serum C3, the baseline characteristics, and the progression of focal segmental glomerulosclerosis (FSGS) | Disease monitoring | FSGS patients | C3, TCC | Serum | No | C3 not specified, TCC ELISA |
| Abdel-Latif M | 28640379 | Comparative and correlative assessments of cytokine, complement and antibody patterns in paediatric type 1 diabetes | Disease monitoring | T1D paptients infected/not infected with enteroviruses | C3d, TCC | Serum | No | ELISA |
| Sereflican B | 28632884 | Determined the levels of complement 3 (C3), acylation-stimulating protein (ASP), and adipsin in Psoriasis | Disease monitoring | Psoriasis patients and matched HC | C3 | Serum | No | Turbidimetric immunoassay |
| Nilsson PH | 28610663 | Identification and investigation of a C5a neoepitope which was exposed on C5 after binding to eculizumab *in-vivo* | Treatment | Sample pre and post eculizumab treatment | C5a | EDTA-plasma | Yes | ELISA |
| Qiao P | 28273946 | Investigate whether CD46 plays a role in Bullous pemphigoid (BP) development | Disease monitoring | BP patients *vs* HCs | C3a | Serum | No | ELISA |
| Song D | 28262211 | Assess complement activation pathways in circulation lupus nephritis patients | Disease monitoring | Patients with renal biopsyproven lupus nephritis vs SLE patients with normal renal function vs HC | C3, C3a, C5a, TCC | Plasma not further specified | ? | ELISA |
| Timmermans SAMEG | 28187980 | Evaluated the role of complement in nine consecutive patients with biopsyproven renal (thrombotic microangiopathy) TMA attributed to severe hypertension | Disease monitoring | Patients with TMA | TCC | Plasma not further specified | ? | ELISA |
| Wehling C | 27784126 | Monitoring of complement activation biomarkers and eculizumab in complement-mediated renal disorders | Treatment | Patients with aHUS, C3G and acute antibody-mediated renal graft rejection (AMR) treated with eculizumab | C3, C3d, C5a, TCC | EDTA-plasma | Yes | ELISA |
| Subías Hidalgo M | 27644115 | Tested eculizumab-treated paroxysmal nocturnal hemoglobinuria (PNH) patients for signs of hemolysis and assessed complement biomarkers | Treatment | PNH patient treated with Eculizumab | C3, TCC | Plasma not further specified | ? | C3 nephelometry, TCC ELISA |

HC=Healthy controls
